# Observing many researchers using the same data and hypothesis reveals a hidden universe of uncertainty

**DOI:** 10.1073/pnas.2203150119

**Published:** 2022-10-28

**Authors:** Nate Breznau, Eike Mark Rinke, Alexander Wuttke, Hung H. V. Nguyen, Muna Adem, Jule Adriaans, Amalia Alvarez-Benjumea, Henrik K. Andersen, Daniel Auer, Flavio Azevedo, Oke Bahnsen, Dave Balzer, Gerrit Bauer, Paul C. Bauer, Markus Baumann, Sharon Baute, Verena Benoit, Julian Bernauer, Carl Berning, Anna Berthold, Felix S. Bethke, Thomas Biegert, Katharina Blinzler, Johannes N. Blumenberg, Licia Bobzien, Andrea Bohman, Thijs Bol, Amie Bostic, Zuzanna Brzozowska, Katharina Burgdorf, Kaspar Burger, Kathrin B. Busch, Juan Carlos-Castillo, Nathan Chan, Pablo Christmann, Roxanne Connelly, Christian S. Czymara, Elena Damian, Alejandro Ecker, Achim Edelmann, Maureen A. Eger, Simon Ellerbrock, Anna Forke, Andrea Forster, Chris Gaasendam, Konstantin Gavras, Vernon Gayle, Theresa Gessler, Timo Gnambs, Amélie Godefroidt, Max Grömping, Martin Groß, Stefan Gruber, Tobias Gummer, Andreas Hadjar, Jan Paul Heisig, Sebastian Hellmeier, Stefanie Heyne, Magdalena Hirsch, Mikael Hjerm, Oshrat Hochman, Andreas Hövermann, Sophia Hunger, Christian Hunkler, Nora Huth, Zsófia S. Ignácz, Laura Jacobs, Jannes Jacobsen, Bastian Jaeger, Sebastian Jungkunz, Nils Jungmann, Mathias Kauff, Manuel Kleinert, Julia Klinger, Jan-Philipp Kolb, Marta Kołczyńska, John Kuk, Katharina Kunißen, Dafina Kurti Sinatra, Alexander Langenkamp, Philipp M. Lersch, Lea-Maria Löbel, Philipp Lutscher, Matthias Mader, Joan E. Madia, Natalia Malancu, Luis Maldonado, Helge Marahrens, Nicole Martin, Paul Martinez, Jochen Mayerl, Oscar J. Mayorga, Patricia McManus, Kyle McWagner, Cecil Meeusen, Daniel Meierrieks, Jonathan Mellon, Friedolin Merhout, Samuel Merk, Daniel Meyer, Leticia Micheli, Jonathan Mijs, Cristóbal Moya, Marcel Neunhoeffer, Daniel Nüst, Olav Nygård, Fabian Ochsenfeld, Gunnar Otte, Anna O. Pechenkina, Christopher Prosser, Louis Raes, Kevin Ralston, Miguel R. Ramos, Arne Roets, Jonathan Rogers, Guido Ropers, Robin Samuel, Gregor Sand, Ariela Schachter, Merlin Schaeffer, David Schieferdecker, Elmar Schlueter, Regine Schmidt, Katja M. Schmidt, Alexander Schmidt-Catran, Claudia Schmiedeberg, Jürgen Schneider, Martijn Schoonvelde, Julia Schulte-Cloos, Sandy Schumann, Reinhard Schunck, Jürgen Schupp, Julian Seuring, Henning Silber, Willem Sleegers, Nico Sonntag, Alexander Staudt, Nadia Steiber, Nils Steiner, Sebastian Sternberg, Dieter Stiers, Dragana Stojmenovska, Nora Storz, Erich Striessnig, Anne-Kathrin Stroppe, Janna Teltemann, Andrey Tibajev, Brian Tung, Giacomo Vagni, Jasper Van Assche, Meta van der Linden, Jolanda van der Noll, Arno Van Hootegem, Stefan Vogtenhuber, Bogdan Voicu, Fieke Wagemans, Nadja Wehl, Hannah Werner, Brenton M. Wiernik, Fabian Winter, Christof Wolf, Yuki Yamada, Nan Zhang, Conrad Ziller, Stefan Zins, Tomasz Żółtak

**Affiliations:** ^a^Research Center on Inequality and Social Policy (SOCIUM), University of Bremen, Bremen, 28359, Germany;; ^b^School of Politics and International Studies, University of Leeds, Leeds, LS2 9JT, United Kingdom;; ^c^Mannheim Centre for European Social Research, University of Mannheim, 68131 Mannheim, Germany;; ^d^Bremen International Graduate School of Social Sciences, 28359 Bremen, Germany;; ^e^Department of Sociology, Indiana University, Bloomington, IN 47405;; ^f^Socio-Economic Panel Study (SOEP), German Institute for Economic Research (DIW), 10117 Berlin, Germany;; ^g^Mechanisms of Normative Change, Max Planck Institute for Research on Collective Goods, 53113 Bonn, Germany;; ^h^Institute of Sociology, Chemnitz University of Technology, 09126 Chemnitz, Germany;; ^i^School of Social Sciences, University of Mannheim, 68159 Mannheim, Germany;; ^j^Department of Psychology, University of Cambridge, Cambridge, CB23RQ, United Kingdom;; ^k^Institute of Sociology, Johannes Gutenberg University Mainz, 55128 Mainz, Germany;; ^l^Department of Political Science, Ludwig Maximilian University, 80539 Munich, Germany;; ^m^Heidelberg University, 69117 Heidelberg, Germany;; ^n^Comparative Political Economy, University of Konstanz, 78457 Konstanz, Germany;; ^o^Faculty of Social Sciences, Economics, and Business Administration, University of Bamberg, 96052 Bamberg, Germany;; ^p^Research Department on Intrastate Conflict, Peace Research Institute Frankfurt, 60329 Frankfurt, Germany;; ^q^Department of Social Policy, London School of Economics and Political Science, London, WC2A 2AE, United Kingdom;; ^r^Survey Data Curation, Leibniz Institute for the Social Sciences (GESIS), 50667 Cologne, Germany;; ^s^Jacques Delors Centre, Hertie School, 10117 Berlin, Germany;; ^t^Department of Sociology, Umeå University, 90187 Umeå, Sweden;; ^u^Department of Sociology, The University of Texas Rio Grande Valley, Brownsville, TX 78520;; ^v^Vienna Institute of Demography, Austrian Academy of Sciences, 1030 Vienna, Austria;; ^w^Austrian National Public Health Institute, Gesundheit Österreich (GÖG), 1030 Vienna, Austria;; ^x^Social Research Institute, Institute of Education, University College London, London, WC1H 0AL, United Kingdom;; ^y^Department of Sociology, University of Zurich, 8050 Zurich, Switzerland;; ^z^Independent researcher;; ^aa^Department of Sociology, University of Chile, Santiago, 7800284, Chile;; ^bb^Department of Political Science, The University of California, Irvine, CA 92617;; ^cc^School of Social and Political Science, University of Edinburgh, Edinburgh, EH8 9LD, United Kingdom;; ^dd^Institute for Political Science, Goethe University Frankfurt, 60323 Frankfurt, Germany;; ^ee^Department of Sociology, Center for Sociological Research, KU Leuven, 3000 Leuven, Belgium;; ^ff^Médialab, Sciences Po, 75007 Paris, France;; ^gg^Educational Measurement, Leibniz Institute for Educational Trajectories, 96047 Bamberg, Germany;; ^hh^School of Government and International Relations, Griffith University, Nathan, QLD, 4111, Australia;; ^ii^Department of Sociology, University of Tübingen, 72074 Tübingen, Germany;; ^jj^Max Planck Institute for Social Law and Social Policy, 80799 Munich, Germany;; ^kk^University of Luxembourg, 4365 Esch-sur-Alzette, Luxembourg;; ^ll^Department of Political Science, Université Libre de Bruxelles, 1050 Bruxelles, Belgium;; ^mm^Wirtschafts- und Sozialwissenschaftliches Institut (WSI), Hans Böckler Foundation, 40474 Düsseldorf, Germany;; ^nn^Berlin Institute for Integration and Migration Research (BIM), Humboldt University Berlin, 10099 Berlin, Germany;; ^oo^School of Human and Social Sciences, University of Wuppertal, 42119 Wuppertal, Germany;; ^pp^Empirical Educational and Higher Education Research, Freie Universität Berlin, 14195 Berlin, Germany;; ^qq^German Socio-Economic Panel Survey, 10117 Berlin, Germany;; ^rr^Department of Social Psychology, Tilburg University, 5037AB Tilburg, The Netherlands;; ^ss^Institute for Socio-Economics, University of Duisburg-Essen, 47057 Duisburg, Germany;; ^tt^Zeppelin University, 88045 Friedrichshafen, Germany;; ^uu^Department of Psychology, Medical School Hamburg, 20457 Hamburg, Germany;; ^vv^Federal Statistics Office Germany, Destatis, 65189 Wiesbaden, Germany;; ^ww^Department of Research on Social and Institutional Transformations, Institute of Political Studies of the Polish Academy of Sciences, 00-625 Warsaw, Poland;; ^xx^Department of Political Science, University of Oklahoma, Norman, OK 73019;; ^yy^Department of Political Science, University of Oslo, 0851 Oslo, Norway;; ^zz^Department of Politics and Public Administration, University of Konstanz, 78457 Konstanz, Germany;; ^aaa^Department of Sociology, Nuffield College, University of Oxford, Oxford, OX1 1JD, United Kingdom;; ^bbb^The Institute of Citizenship Studies (InCite), University of Geneva, 1205 Geneva, Switzerland;; ^ccc^Instituto de Sociologia, Pontifical Catholic University of Chile, Santiago, 7820436, Chile;; ^ddd^Department of Politics, University of Manchester, Manchester, M19 2JS, United Kingdom;; ^eee^Department of Institutional Research, Western Governors University, Salt Lake City, UT 84107;; ^fff^Department of Sociology, University of California, Los Angeles, CA 90095;; ^ggg^Department of Sociology and Centre for Social Data Science, University of Copenhagen, 1353 Copenhagen, Denmark;; ^hhh^Department of School Development, University of Education Karlsruhe, 76133 Karlsruhe, Germany;; ^iii^Department of Psychology III, Julius-Maximilians University Würzburg, 97070 Würzburg, Germany;; ^jjj^Department of Sociology, Boston University, Boston, MA 02215;; ^kkk^Faculty of Sociology, Bielefeld University, 33615 Bielefeld, Germany;; ^lll^Institute of Political Science, University of Münster, 48149 Münster, Germany;; ^mmm^Division of Migration, Ethnicity and Society (REMESO), Linköping University, 60174 Linköping, Sweden;; ^nnn^Administrative Headquarters, Max Planck Society, 80539 Berlin, Germany;; ^ooo^Department of Political Science, Utah State University, Logan, UT 84321;; ^ppp^Department of Politics, International Relations and Philosophy, Royal Holloway University of London, London, TW20 0EX, United Kingdom;; ^qqq^Department of Social Policy, Sociology and Criminology, University of Birmingham, Birmingham, B15 2TT, United Kingdom;; ^rrr^Department of Developmental, Personality and Social Psychology, Ghent University, 9000 Ghent, Belgium;; ^sss^Division of Social Science, New York University Abu Dhabi, Abu Dhabi, 10276, United Arab Emirates;; ^ttt^Department of Sociology, Washington University in St. Louis, St. Louis, MO 63130;; ^uuu^Institute of Sociology, Justus Liebig University of Giessen, 35394 Giessen, Germany;; ^vvv^University College Dublin, Dublin 4, Ireland;; ^www^Department of Sociology, University of Vienna, 1090 Vienna, Austria;; ^xxx^Interdisciplinary Social Science, Utrecht University, 3584 Utrecht, The Netherlands;; ^yyy^Institute for Social Sciences, University of Hildesheim, 31141 Hildesheim, Germany;; ^zzz^Department of Psychology, University of Hagen, 58097 Hagen, Germany;; ^aaaa^Research Institute for Quality of Life, Romanian Academy, 010071 Bucharest, Romania;; ^bbbb^Department of Sociology, Lucian Blaga University of Sibiu, 550024 Sibiu, Romania;; ^cccc^Netherlands Institute for Social Research, 2500 BD The Hague, the Netherlands;; ^dddd^Research Cluster "The Politics of Inequality", University of Konstanz, 78464 Konstanz, Germany;; ^eeee^Department of Psychology, University of South Florida, Tampa, FL 33620;; ^ffff^Faculty of Arts and Science, Kyushu University, Fukuoka, 819-0395, Japan;; ^gggg^Institute for Employment Research, Federal Employment Agency, 90478 Nuremberg, Germany;; ^hhhh^Kulturwissenschaftliche Fakultät, European University Viadrina, 15230 Frankfurt (Oder), Germany;; ^iiii^University of Groningen, 9712 CP Groningen,The Netherlands;; ^jjjj^Cluster "Data-Methods-Monitoring", German Center for Integration and Migration Research (DeZIM),10117 Berlin, Germany;; ^kkkk^Robert Schuman Center for Advanced Studies, European University Institute, 50133 Florence, Italy;; ^llll^University of Fribourg, 1700 Fribourg, Switzerland;; ^mmmm^Center for Social Conflict and Cohesion Studies (COES), Pontificia Universidad Católica de Chile, Santiago, 8331150, Chile;; ^nnnn^Department of Political Science and International Relations, Loyola Marymount University, Los Angeles, CA 90045;; ^oooo^Department of Sociology, Ludwig Maximilian University, 80801 Munich, Germany;; ^pppp^Institute for Political Science, Johannes Gutenberg University Mainz, 55099 Mainz, Germany;; ^qqqq^Institute of Sociology, Goethe University Frankfurt, 60323 Frankfurt, Germany;; ^rrrr^Knowledge Exchange and Outreach, Leibniz Institute for the Social Sciences (GESIS), 68159 Mannheim, Germany;; ^ssss^Data and Research on Society, Leibniz Institute for the Social Sciences, 68159 Mannheim, Germany;; ^tttt^Department of Survey Design and Methodology, Leibniz Institute for the Social Sciences (GESIS), 68159 Mannheim, Germany;; ^uuuu^Department of Sociology, University of Amsterdam, 1001 Amsterdam, The Netherlands;; ^vvvv^Jacobs Center for Productive Youth, University of Zurich, 8050 Zurich, Switzerland;; ^wwww^Department of Security and Crime Science, University College London, London,WC1E 6BT, United Kingdom;; ^xxxx^Institute for Media and Communication Studies, Freie Universität Berlin, 14195 Berlin, Germany;; ^yyyy^Lifestyle and Chronic Diseases, Epidemiology and Public Health, Sciensano, 1000 Brussels, Belgium;; ^zzzz^Center for Political Science Research, KU Leuven, 3000 Leuven, Belgium;; ^aaaaa^Centre for Research on Peace and Development, KU Leuven, 3000 Leuven, Belgium;; ^bbbbb^Department of Migration, Leibniz Institute for Educational Trajectories, 96047 Bamberg, Germany;; ^ccccc^Tübingen School of Education, University of Tübingen, 72074 Tübingen, Germany;; ^ddddd^Department of Social Sciences, Humboldt University Berlin, 10099 Berlin, Germany;; ^eeeee^Center for Social and Cultural Psychology, Université Libre de Bruxelles, 1050 Brussels, Belgium;; ^fffff^Department of Economics, Tilburg University, 5037AB Tilburg, The Netherlands;; ^ggggg^Department of Political Science, University of Duisburg-Essen, 47057 Duisburg, Germany;; ^hhhhh^Department of Geosciences, University of Münster, 49149 Münster, Germany;; ^iiiii^Chair of Political Sociology, University of Bamberg, 96052 Bamberg, Germany;; ^jjjjj^Institute of Sociology and Social Psychology, University of Cologne, 50931 Cologne, Germany;; ^kkkkk^Department of Education and Social Sciences, University of Cologne, 50931 Cologne, Germany;; ^lllll^Institute for the Evaluation of Public Policies, Fondazione Bruno Kessler, 38122 Trento, Italy;; ^mmmmm^Research Group "Health and Social Inequality", Berlin Social Science Center (WZB), 10785 Berlin, Germany;; ^nnnnn^Transformations of Democracy Unit, Berlin Social Science Center (WZB), 10785 Berlin, Germany;; ^ooooo^Research Unit Migration, Integration, Transnationalization, Berlin Social Science Center (WZB), 10785 Berlin, Germany;; ^ppppp^Center for Civil Society Research, Berlin Social Science Center, 10785 Berlin, Germany;; ^qqqqq^Department of Sociology, University of Copenhagen, 1353 Copenhagen, Denmark;; ^rrrrr^Department of Social Sciences, University of Luxembourg, 4366 Esch-sur-Alzette, Luxembourg;; ^sssss^Department of European Languages and Cultures, University of Groningen, 9712 EK Groningen, The Netherlands;; ^ttttt^Department of Demography, University of Vienna, 1010 Vienna, Austria;; ^uuuuu^Education and Employment, Institute for Advanced Studies, University of Vienna, Vienna, 1080 Austria;; ^vvvvv^Policy Perspectives, Citizen Perspectives, and Behaviors, Netherlands Institute for Social Research, 2594 The Hague, The Netherlands;; ^wwwww^President, Leibniz Institute for the Social Sciences (GESIS), 68159 Mannheim, Germany

**Keywords:** metascience, many analysts, researcher degrees of freedom, analytical flexibility, immigration and policy preferences

## Abstract

This study explores how researchers’ analytical choices affect the reliability of scientific findings. Most discussions of reliability problems in science focus on systematic biases. We broaden the lens to emphasize the idiosyncrasy of conscious and unconscious decisions that researchers make during data analysis. We coordinated 161 researchers in 73 research teams and observed their research decisions as they used the same data to independently test the same prominent social science hypothesis: that greater immigration reduces support for social policies among the public. In this typical case of social science research, research teams reported both widely diverging numerical findings and substantive conclusions despite identical start conditions. Researchers’ expertise, prior beliefs, and expectations barely predict the wide variation in research outcomes. More than 95% of the total variance in numerical results remains unexplained even after qualitative coding of all identifiable decisions in each team’s workflow. This reveals a universe of uncertainty that remains hidden when considering a single study in isolation. The idiosyncratic nature of how researchers’ results and conclusions varied is a previously underappreciated explanation for why many scientific hypotheses remain contested. These results call for greater epistemic humility and clarity in reporting scientific findings.

## Significance

Will different researchers converge on similar findings when analyzing the same data? Seventy-three independent research teams used identical cross-country survey data to test a prominent social science hypothesis: that more immigration will reduce public support for government provision of social policies. Instead of convergence, teams’ results varied greatly, ranging from large negative to large positive effects of immigration on social policy support. The choices made by the research teams in designing their statistical tests explain very little of this variation; a hidden universe of uncertainty remains. Considering this variation, scientists, especially those working with the complexities of human societies and behavior, should exercise humility and strive to better account for the uncertainty in their work.

Organized scientific knowledge production involves institutionalized checks, such as editorial vetting, peer review, and methodological standards, to ensure that findings are independent of the characteristics or predispositions of any single researcher (1, 2). These procedures should generate interresearcher reliability, offering consumers of scientific findings assurance that they are not arbitrary flukes and that other researchers would generate similar findings given the same data. Recent metascience research challenges this assumption as several attempts to reproduce findings from previous studies failed (3, 4).

In response, scientists have discussed various threats to the reliability of the scientific process with a focus on biases inherent in the production of science. Pointing to both misaligned structural incentives and the cognitive tendencies of researchers (5–7), this bias-focused perspective argues that systematic distortions of the research process push the published literature away from truth seeking and accurate observation. This then reduces the probability that a carefully executed replication will arrive at the same findings.

Here, we argue that some roots of reliability issues in science run deeper than systematically distorted research practices. We propose that to better understand why research is often nonreplicable or lacking interresearcher reliability, we need to account for idiosyncratic variation inherent in the scientific process. Our main argument is that variability in research outcomes between researchers can occur even under rigid adherence to the scientific method, high ethical standards, and state-of-the-art approaches to maximizing reproducibility. As we report below, even well-meaning scientists provided with identical data and freed from pressures to distort results may not reliably converge in their findings because of the complexity and ambiguity inherent to the process of scientific analysis.

## Variability in Research Outcomes

The scientific process confronts researchers with a multiplicity of seemingly minor, yet nontrivial, decision points, each of which may introduce variability in research outcomes. An important but underappreciated fact is that this even holds for what is often seen as the most objective step in the research process: working with the data after it has come in. Researchers can take literally millions of different paths in wrangling, analyzing, presenting, and interpreting their data. The number of choices grows exponentially with the number of cases and variables included (8–10).

A bias-focused perspective implicitly assumes that reducing “perverse” incentives to generate surprising and sleek results would instead lead researchers to generate valid conclusions. This may be too optimistic. While removing these barriers leads researchers away from systematically taking invalid or biased analytical paths (8–11), this alone does not guarantee validity and reliability. For reasons less nefarious, researchers can disperse in different directions in what Gelman and Loken call a “garden of forking paths” in analytical decision-making (8).

There are two primary explanations for variation in forking decisions. The competency hypothesis posits that researchers may make different analytical decisions because of varying levels of statistical and subject expertise that lead to different judgments as to what constitutes the “ideal” analysis in a given research situation. The confirmation bias hypothesis holds that researchers may make reliably different analytical choices because of differences in preexisting beliefs and attitudes, which may lead to justification of analytical approaches favoring certain outcomes post hoc. However, many other covert or idiosyncratic influences, large and small, may also lead to unreliable and unexplainable variation in analytical decision pathways (10). Sometimes even the tiniest of these differences may add up and interact to produce widely varying outcomes.

There is growing awareness of the dependence of findings on statistical modeling decisions and the importance of analytical robustness (9, 11–13). However, only recently scientists began to assess whether researcher variability affects scientific outcomes in reality, sometimes employing “many analysts” approaches where many researchers or teams independently test the same hypothesis with the same data. The first such study showed that when 29 researchers tested if soccer referees were biased toward darker-skin players using the same data, they reported 29 unique model specifications, with empirical results ranging from modestly negative to strongly positive (14). Thus far, most many-analysts studies have been small in scale or focused on narrow, field-specific analysis methods (15, 16). Recent studies by Botvinik-Nezer et al. (17) and Menkveld et al. (18) were larger, involving 65 and 164 teams, respectively. Critically, despite their size, these studies also found large interresearcher variation in reported results. They also made first steps at explaining the amount of variation in reported results using a small set of variables, such as the computational reproducibility and peer ratings of submitted analyses or the statistical software that analysts used. Yet, they had little success in explaining the variation in results or the distance of results from the overall mean of the results (i.e., error). We expanded on these explanatory attempts by observing every step of each independent research team’s workflow, expecting that such close observation should explain far more variance in research outcomes. Moreover, when coupled with measures of relevant analytical competencies and substantive beliefs of the analysts as explanatory variables, we expected to arrive at a deeper understanding of the results and crucially, which key decisions drive them.

## Methods

The principal investigators (PIs) coordinated a group of 161 researchers in 73 teams to complete the same task of independently testing a hypothesis central to an “extensive body of scholarship” (19) in the social sciences: that immigration reduces support for social policies among the public.* Our entire reproducible workflow for this study is available online in our Project Repository.^†^ The task given to the participants is typical for research on human societies, in which the central concepts and quantities of interest are open to broad and complex interpretations (20, 21). In classic political economy research, for example, Alberto Alesina and Edward Glaeser (22, 23) hypothesized that differences in North American and European social security systems are a result of immigration-generated ethnic diversity or a lack thereof. More recently, other scholars see immigration and refugee crises as catalysts of retrenchment of social security systems in Western Europe and across the globe. Put simply, this hypothesis was given to participating teams because it is influential, long standing, and typical for contemporary social research in political science, sociology, economics, geography, and beyond (24–29).

We recruited participants by circulating a call across academic networks, social media, and official communication channels of academic associations across social science disciplines (*SI Appendix*, *Research Design*). Although 106 teams expressed interest in participation, we count our initial sample as 88 that completed the prestudy questionnaire. In the end, 73 of those 88 teams (a total of 161 researchers with an average of 2.24 researchers per team) completed the study. Of these, 46% had a background in sociology; 25% had a background in political science; and the rest had economics, communication, interdisciplinary, or methods-focused degree backgrounds. Eighty-three percent had experience teaching courses on data analysis, and 70% had published at least one article or chapter on the substantive topic of the study or the usage of a relevant method (*SI Appendix*, *III. Participant Survey Codebook* has more participant details).

The PIs provided teams with data from the International Social Survey Program (ISSP), a long-running large-scale cross-nationally comparative survey of political and economic attitudes used in over 10,000 published studies.^‡^ The ISSP includes a six-question module on the role of government in providing different social policies, such as old-age, labor market, and health care provisions. This six-question module is also the source of the data used by David Brady and Ryan Finnigan (19) in one of the most cited investigations of the substantive hypothesis participants were instructed to test. The PIs also provided yearly indicator data for countries on immigrant 'stock' as a percentage of the population and on 'flow' as a net change in stock, taken from the World Bank, the United Nations, and the Organization for Economic Co-Operation and Development. Relevant ISSP and immigration data were available for 31 mostly rich and some middle-income countries. There were up to five survey waves from 1985, 1990, 1996, 2006, and 2016. All provided data come from publicly available sources.

To remove potentially biasing incentives, all researchers from teams that completed the study were ensured coauthorship on the final paper regardless of their results. Because the “participants” themselves were researchers and all tasks assigned to them were standard research practices theoretically worthy of coauthorship, institutional review prior to conducting this study was not necessary. The researchers participated in surveys to measure expertise and study-relevant beliefs and attitudes before and during the research process. Moreover, they took part in online deliberations before (a randomized half of teams) and after they had run their main analyses (all teams) (*SI Appendix, III. Participant Survey Codebook*). To familiarize participating researchers with the data, their first task was to numerically reproduce results from the Brady and Finnigan (19) study on a subset of the ISSP data. This was followed by a request that the teams develop their own ideal models for testing the same hypothesis using potentially all of the provided data, but that they submit their analysis plan prior to running the models. To enhance ecological validity, we allowed the teams to include additional data sources for measuring independent variables. Each team was then instructed to run their model(s) and report dependent variable–standardized effect estimates equal to the change in policy preferences (in SD units) predicted by a one-point change in the respective independent immigration variable. We also asked them to draw one of three subjective conclusions: whether their results offered evidence that supported the hypothesis that immigration reduces support for social policies among the public, whether their results offered evidence that rejected the hypothesis, or instead, whether they believed the hypothesis was not testable given these data.

Of the 73 teams, 1 conducted preliminary measurement scaling tests, concluded that the hypothesis could not be reliably tested, and thus, did not design or carry out any further tests. This left 72 teams submitting a total of 1,261 models. One team’s preregistered models failed to converge and thus, had no numerical results. This left a total of 71 teams with numerical results from 1,253 models. In their subjective conclusions, 16 teams determined that the two different measures of immigration should be considered independent hypothesis tests and therefore, submitted different conclusions for each. This changed the primary unit of analysis for subjective conclusions from 73 team conclusions to 89 team-level conclusions.

There was an average of 17.5 models per team among the 71 teams submitting numerical results (ranging from 1 to 124 models per team). Most teams submitted at least 12 models because they used each of the six ISSP questions as a single dependent outcome twice in their statistical models, once for each version of the immigration measure (stock and flow). Several teams submitted 18 models because they ran an additional 6 models with both immigration variables included. The teams often adjusted for the nested nature of the data, accounting for variance at the individual, country, year, and/or country-year levels. Some made no such hierarchical adjustments with multilevel models, and others used clustering of the SEs at the country, wave, and/or country-wave levels. Some used dummy interactions for example world region indicators (such as Eastern Europe) or political party preferences with immigration variables, leading to nonlinear predicted values. Others used alternative estimators based on maximum likelihood or Bayesian estimation as opposed to ordinary least squares (*SI Appendix*, Table S3 shows the most common model decisions). In all, researchers’ modeling decisions reflected the diversity of similar, but technically distinct, methodological approaches currently used in contemporary research.

**Fig. 1.**
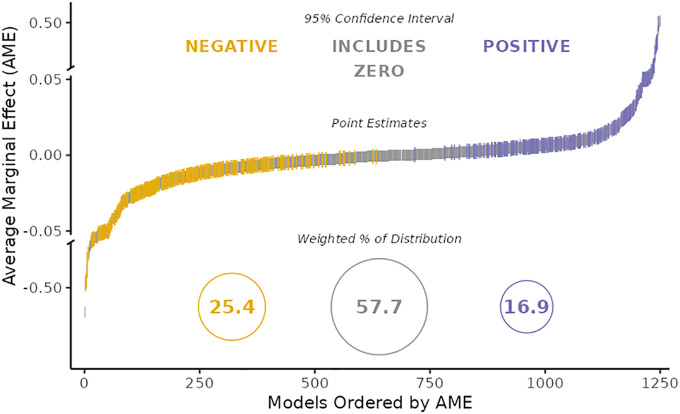
Broad variation in the findings from 73 teams testing the same hypothesis with the same data. The distribution of estimated AMEs across all converged models (*n* = 1,253) includes results that are negative (yellow; in the direction predicted by the given hypothesis the teams were testing), not different from zero (gray), or positive (blue) using a 95% CI. AME are *xy* standardized. The *y* axis contains two scaling breaks at ±0.05. Numbers inside circles represent the percentages of the distribution of each outcome inversely weighted by the number of models per team.

Each team’s code was checked and then anonymized for public sharing by the PIs. Some teams failed to report a standardized estimate. Also, different scaling of the two independent immigration variables meant that results were not always distributionally comparable. Therefore, we standardized the teams’ results for each coefficient for stock and flow of immigration post hoc. We also transformed the teams’ results into average marginal effects (AMEs), which are the standardized average effects of a one-unit change in the respective independent (immigration) variable on the respective dependent (policy support) variable, where this average is based on predictions for each observation in the dataset. The advantage of using AMEs is that they allow for a single marginal estimate in the presence of nonlinearities and present predicted probabilities that reflect the reality of the data sample rather than the mean of each independent variable (Fig. 1 shows results). After submitting their own results but prior to seeing the other teams’ results, each participant was randomly given a rough description of the models employed by four to five other teams and asked to rank them on their quality for testing the hypothesis. With six to seven rankings per team, the PIs constructed model rankings (*SI Appendix*, *Model Ranking*).

At any point after they submitted their results, including after results of the other teams were revealed, the teams could change their preferred models and resubmit their results and conclusion. No team voluntarily opted to do this. However, some teams’ results and conclusions changed after they were informed that the PIs were unable to reproduce their findings due to coding mistakes or a mismatch between their intended models and those that appeared in the code.

Next, we examined all 1,261 models and identified 166 distinct research design decisions associated with those models. “Decision” refers to any aspect in the design of a statistical model: for example, the measurement strategy, estimator, hierarchical structure, choice of independent variables, and potential subsetting of the data (*SI Appendix*, Table S12). For simplicity, decision also refers to variables measuring team characteristics, such as software used, overall familiarity with the subject or methods, and preexisting beliefs as measured in our participant survey (*SI Appendix*, Table S1). Of the 166 decisions, 107 were taken by at least three teams. We used these 107 as variables that might statistically explain the variation in the results and conclusions because the other 59 were unique to one or two teams and would thus impede statistical identification. In other words, uniquely identifying one or two teams' results via the variance in a single independent variable in the regression would interrupt the parsimonious estimation of residual, unexplained variance calculated in the level 2 equation. A dissimilarity matrix revealed that no two models of 1,261 were 100% identical.

To explore the sources of variance in results, we regressed the numerical point estimates and subjective conclusions on all different combinations and interactions of the 107 decisions. We used multilevel regression models, allowing us to account for models nested in teams and to explain total, within-team, and between-team variance in numerical results. For subjective conclusions, we used multinomial logistic regressions predicting teams’ conclusions to 1) support or 2) reject the target hypothesis, or 3) regard it as not testable. Our analyses proceeded in several stages. At each stage, decision variables and their interactions were tested, and only terms that explained the most variance using the least degrees of freedom (or deviance in the case of subjective conclusions) were carried to the next phase.

**Fig. 2.**
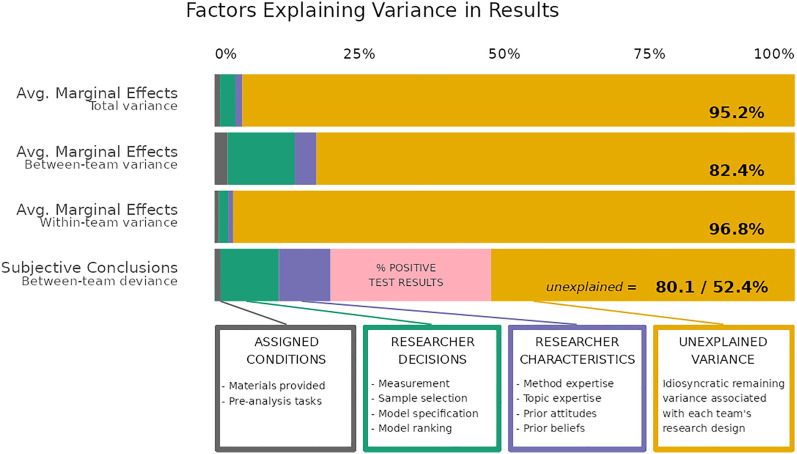
Variance in statistical results and substantive conclusions between and within teams is mostly unexplained by conditions, research design, and researcher characteristics. Decomposition of numerical variance taken from generalized linear multilevel regression models' AMEs (the top three rows). Explained deviance taken from multinomial logistic regressions using the substantive conclusions about the target hypothesis as the outcome submitted by the research teams (bottom row). We used informed stepwise addition and removal of predictors to identify which specifications could explain the most numeric variance (*SI Appendix*, Table S6) and others that could explain the most subjective conclusion deviance (*SI Appendix*, Table S7) while sacrificing the fewest degrees of freedom and maintaining the highest level of model fit based on log likelihood and AIC. We also used algorithms to test variable combinations, but these could not explain more meaningful variation (*Methods*). Assigned conditions were the division of participants into two different task groups and two different deliberation groups during the preparatory phase. Identified researcher decisions are the 107 common decisions taken in data preparation and statistical modeling across teams and their models. Researcher characteristics were identified through a survey of participants and multiitem scaling using factor analysis (*SI Appendix*, Fig. S3). The reader will find many other details in *SI Appendix*.

Exploring the variance in results runs the risk of overfitting. It is statistically inappropriate to use 107 variables when there are 87 team-test cases (from 71 teams with numerical results). Therefore, we entered the variables in groups and only kept variables from each group that showed an increase in the explained variance without a loss in fit as measured by Akaike's Information Criterion (AIC) and log likelihood. This started with “design” decisions, including which of the six survey questions the teams used as the dependent variable in a given model and dummies indicating the two random experimental treatments (which were included in the study but are unrelated to the main modeling task assigned to the teams). The next stage added “measurement” decisions, including which immigration measure the team used in a given model and how the dependent variable was measured (dichotomous, ordinal, multinomial, or continuous). The following stages added “data and sample” and “model design” decisions and concluded with the addition of “researcher aspects” (Project Repository, 04_CRI_Main_Analyses). We also reran the phase-wise analysis separately for each of the six survey questions used as dependent variables by the teams (Project Repository, 07_CRI_DVspecific_Analyses) (*SI Appendix*, Tables S4 and S9–S11). The exact variables in our final analysis plus various models leading up to them are found in *SI Appendix*, Tables S5 and S7. Fig. 2 reports the explained variance from our preferred model m13.

To check the robustness of our phase-wise strategy, we used an algorithm to analyze all possible variable combinations from our primary variables of interest—those that showed any capacity to explain variance in the main analyses (Project Repository, 06_CRI_Multiverse). This led us to a slightly different ideal model than m13 (Auto_1 in *SI Appendix*, Table S10). Although this alternative model had the best AIC and could explain slightly more model-level variance, it could not explain as much total variance. We then combined all variables from our main model (m13) and the algorithm-derived model (Auto_1) to generate a new model (Auto_1_m13). Although this new model explained more variance and had lower AIC, we were careful not to overfit because it had 22 variables, whereas m13 and Auto_1 had 18 and 15, respectively.

Next, based on PNAS peer review feedback, we generated a list of every possible interaction pair of all 107 variables. Of these 5,565 interactions, 2,637 have nonzero variance and thus, were usable in a regression without automatically being dropped. Including 8 or more interaction variables plus their main effects (i.e., 24 or more variables, many that were cross-level interactions) led to convergence or overidentification problems. Even with 87 level 2 cases, this leaves us with roughly 4 cases per variable. Research reviewing different simulation studies on level 2 case numbers suggests that 10 cases per variable are a bare minimum, and we should have closer to 50 ideally (30). Therefore, we settled on 7 interacted variables as our absolute maximum (which corresponds to 21 variables, including two main effects for each interaction). We then randomly sampled 1,000 sets of 7 variables from the list of all and let the algorithm run every subcombination of these, which led to just over 1 million models. We then took the 2 models with the lowest AIC score from each of the 1,000 iterations and extracted all variables from those models. There were 19 unique variables among the 2,000 best-fitting models in total, which we then analyzed using the same “random-seven” sampling method. The best-fitting models from this second iteration left us with four interaction variables as candidates to explain more variance while avoiding sacrificing the simplicity of the model and overfitting (as indicated by model AIC). We added each of these variables separately to our initial algorithm-generated results. None of these models could explain more variance in research outcomes than m13 or Auto_1_m13 (*SI Appendix*, Table S10, Auto_2 to Auto_5).

## Main Results

Fig. 1 visualizes the substantial variation of numerical results reported by 71 researcher teams that analyzed the same data. Results are diffuse. Little more than half the reported estimates were statistically not significantly different from zero at 95% CI, while a quarter were significantly different and negative, and 16.9% were statistically significant and positive.

We observe the same pattern of divergent research outcomes when we use the teams’ subjective conclusions rather than their statistical results. Overall, 13.5% (12 of 89) of the team conclusions were that the hypothesis was not testable given these data, 60.7% (54 of 89) were that the hypothesis should be rejected, and 28.5% (23 of 89) were that the hypothesis was supported (*SI Appendix*, Figs. S5, S9, and S10).^§^

**Fig. 3.**
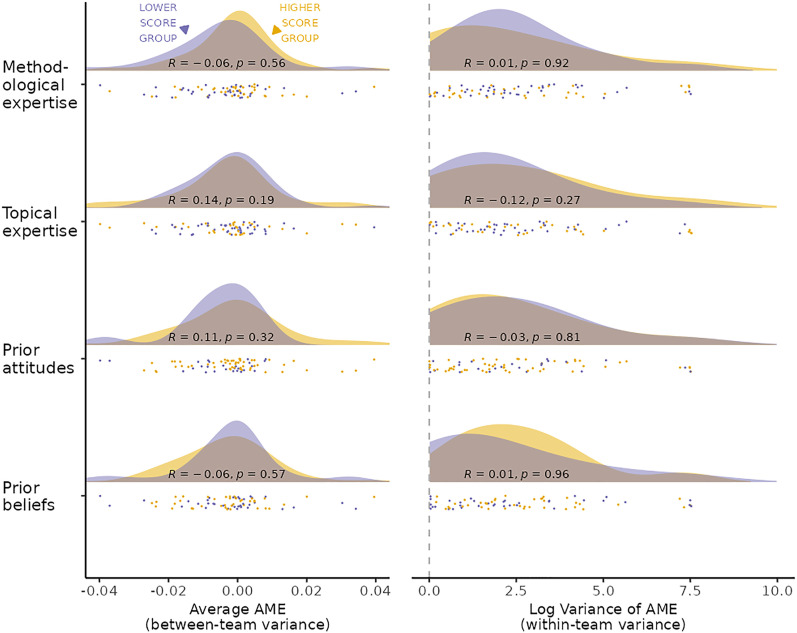
Researcher characteristics do not explain outcome variance between teams or within teams. The distribution of team average of AMEs (*Left*) and within-team variance in AMEs (*Right*) across researchers grouped according to mean splits (“lower” and “higher”) on methodological and topic expertise (potential competencies bias) and on prior attitudes toward immigration and beliefs about whether the hypothesis is true (potential confirmation bias). Log variance was shifted so that the minimum log value equals zero. Teams submitting only one model assigned a variance of zero. Pearson correlations along with a *P* value (“*R*”) are calculated using continuous scores of each researcher characteristic variable.

We find that competencies and potential confirmation biases do not explain the broad variation in outcomes; researcher characteristics show a statistically significant association with neither statistical results nor substantive conclusions (Fig. 3). Hence, the data are not consistent with the expectation that outcome variability simply reflects a lack of knowledge among some participants or preexisting preferences for particular results.

In principle, variation in outcomes must reflect prior decisions of the researchers. Yet, Fig. 2 shows that the 107 identified decision points explain little of the variation. The major components of the identified researcher decisions explain less than a quarter of the variation in four measures of research outcomes. Most variance also remains unexplained after accounting for researcher characteristics or assignment to a small experiment (not reported in this study) (“assigned conditions” in Fig. 2). Looking at total variance in the numerical results (top bar), identified components of the research design explain 2.6% (green segment), and researcher characteristics only account for a maximum of 1.2% of the variance (violet segment). In other words, 95.2% of the total variance in results is left unexplained, suggesting that massive variation in reported results originated from idiosyncratic decisions in the data analysis process.

The share of explained variance is somewhat higher when looking at between-team results (second bar), but still, 82.4% remained unexplained. Variance continues to remain mostly unexplained when moving away from the numerical results and considering researchers’ substantive conclusions (bottom bar; 80.1% unexplained). It is noteworthy that even the percentage of test results per team that statistically support their conclusions explains only about a third of the deviance in conclusions (salmon-colored segment in the bottom bar), which points at the variation in how different researchers interpret the same set of numerical results. Overall, the complexity of the data-analytic process leads to variation that cannot be easily explained, even with a close look at researcher characteristics and researcher decisions.

Finally, we followed previous many-analysts research (31) by creating a benchmark for the above results via a multiverse simulation of possible model specifications. Using the same approach, we found that 23 decisions could explain just over 16% of the variance in numerical outcomes among 2,304 simulated models (*SI Appendix*, Table S8). In contrast to our ecological research setting observing actual researcher behaviors, we fall far short of this 16% simulated total explained variance by almost 12 percentage points. Even with the use of an algorithm to sample all possible variable combinations, we remain over 11 percentage points short.

## Discussion

Results from our controlled research design in a large-scale crowdsourced research effort involving 73 teams demonstrate that analyzing the same hypothesis with the same data can lead to substantial differences in statistical estimates and substantive conclusions. In fact, no two teams arrived at the same set of numerical results or took the same major decisions during data analysis. Our finding of outcome variability echoes those of recent studies involving many analysts undertaken across scientific disciplines. The study reported here differs from these previous efforts because it attempted to catalog every decision in the research process within each team and use those decisions and predictive modeling to explain why there is so much outcome variability. Despite this highly granular decomposition of the analytical process, we could only explain less than 2.6% of the total variance in numerical outcomes. We also tested if expertise, beliefs, and attitudes observed among the teams biased results, but they explained little. Even highly skilled scientists motivated to come to accurate results varied tremendously in what they found when provided with the same data and hypothesis to test. The standard presentation and consumption of scientific results did not disclose the totality of research decisions in the research process. Our conclusion is that we have tapped into a hidden universe of idiosyncratic researcher variability.

This finding was afforded by a many-analysts design as an approach to scientific inquiry. Some scholars have proposed multiverse analysis to simulate analytic decisions across researchers (31), a method to provide a many-analysts set of outcomes without the massive coordination and human capital commitment otherwise necessary for such a study. The drawback of a simulation approach is that it is constructed based on a single data analysis pipeline from one research team and may not reflect the complex reality of different research processes carried out by different teams in different contexts. This study observed researchers in a controlled yet ecological work environment. In doing so, it revealed the hidden universe of consequential decisions and contextual factors that vary across researchers and that a simulation, thus far, cannot capture.

### Implications.

Researchers must make analytical decisions so minute that they often do not even register as decisions. Instead, they go unnoticed as nondeliberate actions following ostensibly standard operating procedures. Our study shows that, when taken as a whole, these hundreds of decisions combine to be far from trivial. However, this understanding only arises from the uniqueness of each of the 1,253 models analyzed herein. Our findings suggest reliability across researchers may remain low even when their accuracy motivation is high and biasing incentives are removed. Higher levels of methodological expertise, another frequently suggested remedy, did not lead to lower variance either. Hence, we are left to believe that idiosyncratic uncertainty is a fundamental feature of the scientific process that is not easily explained by typically observed researcher characteristics or analytical decisions.

These findings add a perspective to the metascience conversation, emphasizing uncertainty in addition to bias. The conclusion warranted from much of the metascience work carried out in the wake of the “replication crisis” in psychology and other fields has been that published research findings are more biased than previously thought. The conclusion warranted from this and other similar studies is that published research findings are also more uncertain than previously thought.

As researchers, we bear the responsibility to accurately describe and explain the world as it is but also, to communicate the uncertainty associated with our knowledge claims. Although the academic system privileges innovation over replication, providing a novel answer to a question is just as essential as informing about how much trust we can place in that answer. Our study has shown that to fully assess and address uncertainty, replications are valuable but insufficient. Only large numbers of analyses may show whether in a specific field, independent researchers reliably arrive at similar conclusions, thus enhancing or undermining our confidence in a given knowledge claim.

Specifically, we believe that serious acknowledgment of idiosyncratic variation in research findings has at least four implications for improving the presentation and interpretation of empirical evidence. First, contemplating that results might vary greatly if a given study had been conducted by a different set of researchers or even the same researchers at a different time, calls for epistemic humility when drawing conclusions based on seemingly objective quantitative procedures. Second, the findings remind us to carefully document everything because, in combination, even the most seemingly minute decisions could drive results in different directions; and only awareness of these minutae could lead to productive theroetical discussions or empirical tests of their legitimacy. Third, countering a defeatist view of the scientific enterprise, this study helps us appreciate the knowledge accumulated in areas where scientists do converge on expert consensus—such as the human impact on the global climate or a notable increase in political polarization in the United States over the past decades. Fourth, our study suggests that if we freed scientists from bias caused by the “perverse incentives” inherent in the institutions of science, their own preexisting biases or beliefs might not matter as much to the outcomes they generate as some may fear. In fairness, the teams indicated what models they intended to run in advance. Although they were free to update and change these at any time, we can assume that this may have reduced potential confirmation bias.

### Limitations and Outlook.

Our study has limitations that warrant discussion. First, we do not know the generalizability of our study to different topics, disciplines, or even datasets. A major segment of social science works with survey data, and our results reflect this type of research. In experimental research, the data-generating model is often clearer or involves fewer decisions. Moreover, in social research, there are no Newtonian laws or definite quantum statistical likelihoods to work with, suggesting that our case might overestimate variability compared with the natural sciences. On the other hand, functional magnetic resonance imaging (fMRI), gene, and space telescope data, for example, are far more complex than what we gather in social surveys. The complexity of data analysis pipelines is correspondingly greater in these fields, but it is possible that having more analytical decisions allows us to account for more of the variation in research outcomes. We believe that the number of decision points in data analysis and the extent to which researchers understand the data-generating process may determine the degree of outcome variation in a field, but it remains an open question if and how design decisions play a smaller or larger role across fields.

Second, although we hoped to offer deeper insights on the substantive hypothesis under observation, we did not obtain evidence that moves conclusions in any direction. These lessons combined with the fact that a substantial portion of participants considered the hypothesis not testable with these data offer a potential explanation for why this is such a contested hypothesis in the social sciences (19, 24, 32).

Looking forward, we take the fact that 13.5% of participating analysts claimed that the target hypothesis is “not testable” with the provided data as a powerful reminder of the importance of design appropriateness and clear specification of hypotheses. This implicates clarity in the meaning of a conclusion. In our study, “support” of the hypothesis generally meant rejection of the null, whereas “reject” meant consistency with the null or inconclusive results. Teams were left to their own devices in deciding what constituted support for, evidence against, or nontestability of the target hypothesis, and this alone introduced a degree of indeterminacy into the research process. Overall, these observations call for more attention to conceptual, causal, and theoretical clarity in the social sciences as well as for the gathering of new data when results no longer appear to move a substantive area forward (20, 21). They also suggest that if we want to reap epistemic benefits from the present move toward open research practices, we must make more conscious efforts to complement methodological transparency with theoretical clarity.

Finally, we note that the conclusions of this study were themselves derived from myriad seemingly minor (meta-)analytical decisions, just like those we observed among our analysts. We, therefore, encourage readers to scrutinize our analytical process by taking advantage of *SI Appendix*, the reproduction files, and the web-based interactive app that allow for easy exploration of all data underlying this study.^¶^

## Supplementary Material

Supplementary File

## Data Availability

Data and code have been deposited in GitHub (https://github.com/nbreznau/CRI) (33), and Harvard Dataverse (https://doi.org/10.7910/DVN/UUP8CX) (34).

## Footnotes

^*^*SI Appendix*, Figs. S1 and S2 and Tables S1 and S2 have the time line and more participant details.

^†^It is available at https://github.com/nbreznau/CRI.

^‡^Information is available at https://issp.org/about-issp/.

^§^This is a reminder that 16 teams reported two differing conclusions based on their interpretation of different model specifications, causing the *N* to jump from 72 teams to 89 team-conclusion units of analysis (*Methods*).

^¶^Information is available at https://nate-breznau.shinyapps.io/shiny/ and https://github.com/nbreznau/CRI.
