# Supplementary material for: Observing many researchers using the same data and hypothesis reveals a hidden universe of uncertainty
Source: Proc Natl Acad Sci U S A. 2022 Oct 28;119(44):e2203150119. doi: 10.1073/pnas.2203150119 (PMC9636921; doi:10.1073/pnas.2203150119)
Supplement: Supplementary File [file pnas.2203150119.sapp.pdf]

Supplementary Materials for

Observing Many Researchers using the Same Data and Hypothesis Reveals a Hidden Universe of Data Analysis

Table of Contents

List of Tables and Figures ... 2
Overview ... 3
Article Abstract ... 3
Abstract for Supplementary Materials ... 3
Research Design ... 4
Timeline ... 4
Pre-Analysis Plan ... 6
Hypothesis tested by Participants ... 6
Communications ... 7
Participant Materials ... 7
Researcher Characteristics ... 8
Participant Survey ... 8
Measurement of Characteristics ... 9
Model Ranking ... 12
Team Results ... 16
Effects and Conclusions ... 16
Model Specification Coding ... 17
Common Models ... 19
Explaining Result Variance ... 21
Visualizing Result Variance ... 21
Main Regression Models ... 26
Multiverse Analysis – How Much Variance Should we Expect to Explain? ... 35
DV-Specific Regression Models ... 36
Dredging Variance Function ... 37
Variance Function Regression ... 39
Supplementary Material Appendix ... 41
I. Communications ... 41
II. Model Specification Coding and Distribution ... 53
III. Participant Survey Codebook ... 80
IV. Model Ranking during Participant Survey, Wave 4 ... 111
Model Descriptions for Ranking ... 111
Ranking Derived from Deliberation and Voting ... 188
References ... 190

List of Tables and Figures

**Fig S1. Timeline of the CRI project**	4
**Fig S2. Teams and Participants by Stage**	5
**Table S1. Selected researcher characteristics**	8
**Fig S3. Measurement model for researcher characteristics**	11
**Fig S4. Subjective ranking of model specifications**	14
**Fig S5. Distribution of effect sizes and subjective conclusions**	16
**Fig S6. Specification curves by researcher characteristics and model score**	21
**Fig S7. Specification curves from Fig S6 plotted against measurement decisions**	22
**Fig S8. Specification curves from Fig S6 plotted against sample and estimation methods**	23
**Fig S9. Subjective conclusions by team plotted against researcher characteristics**	24
**Fig S10. Subjective conclusions by percentage of supporting test results**	25
**Fig S11. Unexplained team and model-level variance by researcher characteristics**	33
**Fig S12. Intercept and unexplained variance by researcher characteristics**	34
**Table S2. Correlations among researcher characteristics**	10
**Table S3. Selected frequencies of model specifications out of 1,261 models**	18
**Table S4. Common model specifications by sub-domain clusters**	20
**Table S5. Multilevel linear regression predicting variance in AME by model and team**	27
**Table S6. Model statistics for Table S5**	28
**Table S7. Multinomial regression predicting support for hypothesis**	29
**Table S8. Multiverse results explaining simulated researcher variability**	35
**Table S9. DV-specific regressions predicting AMEs**	36
**Table S10. Computer-assisted variance explained**	37
**Table S11. Variance function regression results**	40
**Table S12. Model specification and coding**	63

Overview

Article Abstract

This study explores how researchers' analytical choices affect the reliability of scientific findings. Most discussions of reliability problems in science focus on systematic biases. We broaden the lens to include conscious and unconscious decisions that researchers make during data analysis and that may lead to diverging results. We coordinated 161 researchers in 73 research teams and observed their research decisions as they used the same data to independently test the same prominent social science hypothesis: that greater immigration reduces support for social policies among the public. In this typical case of research based on secondary data, we find that research teams reported both widely diverging numerical findings and substantive conclusions despite identical start conditions. Researchers' expertise, prior beliefs, and expectations barely predict the wide variation in research outcomes. More than 90% of the total variance in numerical results remains unexplained even after accounting for research decisions identified via qualitative coding of each team's workflow. This reveals a universe of uncertainty that is otherwise hidden when considering a single study in isolation. The idiosyncratic nature of how researchers' results and conclusions varied is a new explanation for why many scientific hypotheses remain contested. It calls for greater humility and clarity in reporting scientific findings.

Abstract for Supplementary Materials

This particular study was one phase of the Crowdsourced Replication Initiative, a collaborative 'many analyst' project designed by Nate Breznau, Alexander Wuttke and Eike Mark Rinke while working at the Mannheim Centre for European Social Research (MZES) at the University of Mannheim, Germany. The Timeline (Fig S1) offers a general overview of the stages and timing of the research. All steps of data preparation, coding and analyses[1] except those that would risk the anonymity of participants are available in our Project Repository[2]. More details about the other studies conducted within the Crowdsourced Replication Initiative and details not directly related to the findings in this study can be found in the Executive Report.

[1] We make use of ggplot(1) and raincloud plot(2) packages for visualizations and lavaan(3) for measurement modeling

[2] https://github.com/nbreznau/CRI

Research Design

Timeline

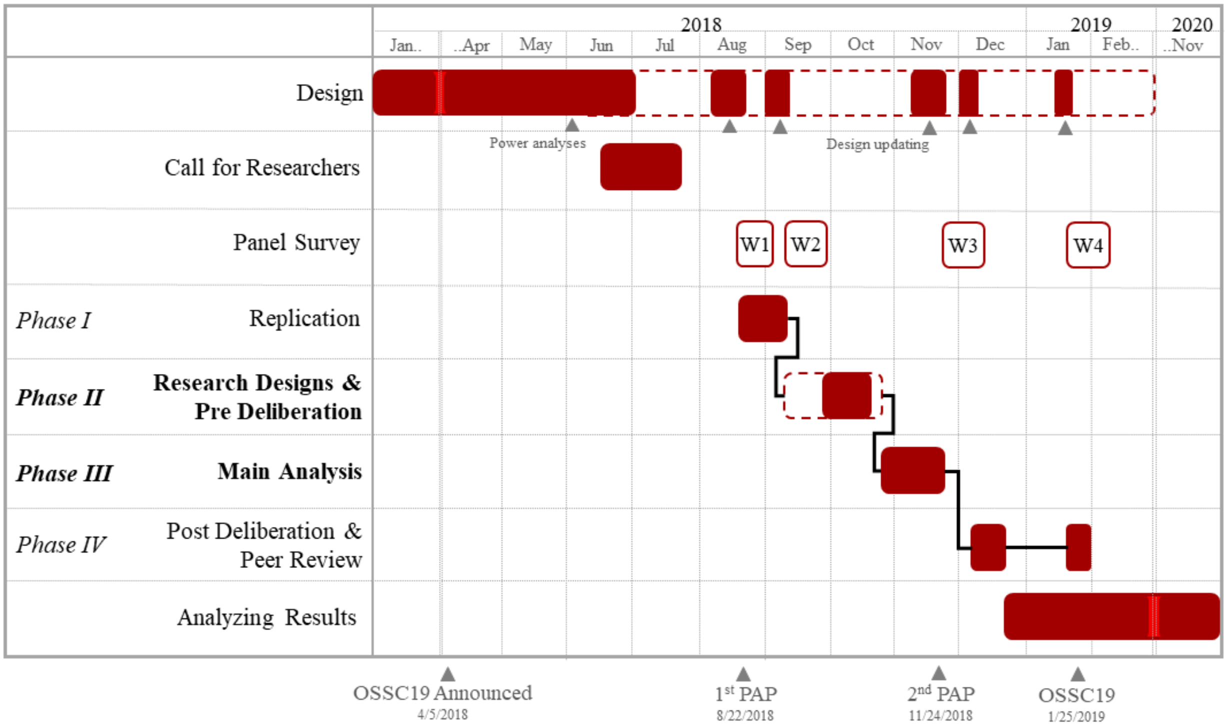

Fig S1. Timeline of the CRI project
Design took place from January 2018 through February 2019, data were collected from July 2018 through December 2018, coding, principal investigator's replication of each team's results and analyses took place from Feb 2019 until November 2020. "Phase I" through "Phase IV" were tasks assigned to the Participants. "OSSC19" refers to an open social science conference that helped inspire the project and where the preliminary results were presented. 'PAP' means pre-analysis plan and the date is when we pre-registered each PAP on the Open Science Framework.

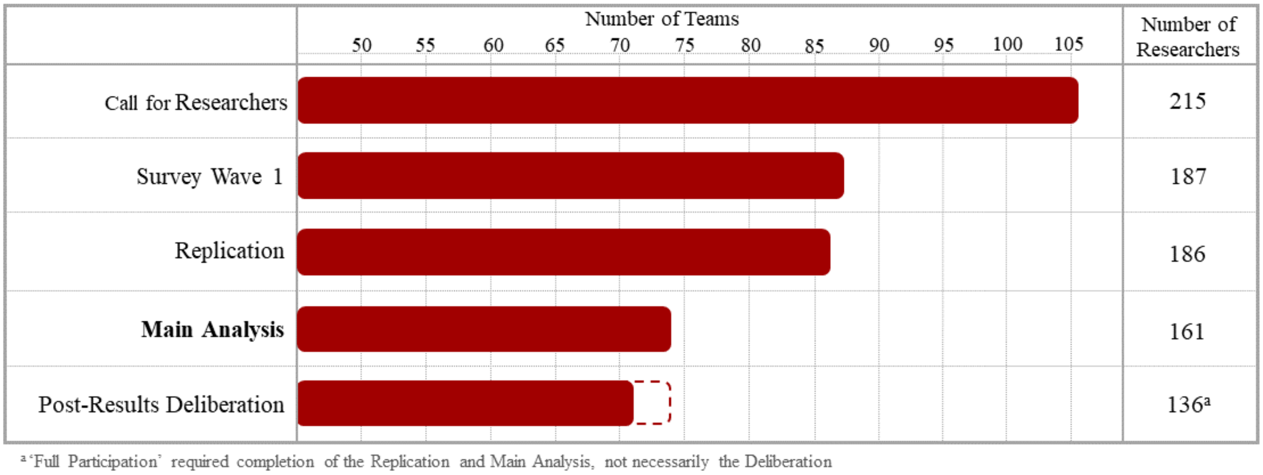

Fig S2. Teams and Participants by Stage

At first, 215 researchers volunteered to participate, but only 187 completed the first participant survey wave (88 teams). More researchers dropped out over time and the final number of Participants was 161 in 73 teams; average 2.24 per team. One researcher dropped out from their team about two-thirds of the way through, meaning several waves of survey data are available for 162 participants. 'Full Participation' in the study included completion of the Main Analysis task to test the hypothesis with the given data. Post-Results Deliberation was optional. See Codebook, p. 80 for more details. Breakdown by reported discipline: 1.9% Communication, 4.7% Economics, 46% Sociology, 24.9% Political Science, 4.2% Methods-Related Degree, 6.1% Other, and 4.2% non-response.

Pre-Analysis Plan

We posted a pre-analysis plan (PAP) on the Open Science Framework on 22/8/2018 focusing on our general plan and a power analysis to determine how many researchers we required for the crowdsourced project(4). This pre-analysis plan was not peer reviewed and it did not cover the qualitative coding of model specifications. We cite it for transparency and so the reader understands our expectations *that we would be able to explain results of crowdsourced researchers testing the same hypothesis based on their* ***model specifications*** *and* ***researcher characteristics***. As a result, we expected to then know something more about the actual hypothesis being tested.

Hypothesis tested by Participants

<u>Hypothesis:</u>

That greater immigration reduces support for social policies among the public.

<u>Data:</u>

International Social Survey Program (ISSP) waves I-V (1985-2016), plus various macro-indicators.

Communications

The 'Call for Researchers' (see Fig S1) was put out in a web portal hosted by the Mannheim Centre for European Social Research at the University of Mannheim, Germany. It simultaneously advertised the open science conference ("OSSC", starting on 4/5/2018) and the Call for Researchers from 20/6/2018 until 27/7/2018. The verbatim text is in Communication S1. The following Communications S2-S10 are the verbatim emails sent to participants with greetings, salutations and informational links removed. Throughout the process there were several emails exchanged between the Principal Investigators (PIs) and the Participants. Communications S1-S4 introduced the Participants to the project, gave them survey instructions and asked them to conduct a replication of a study reflecting the state of the art in social science research testing the hypothesis that *immigration undermines support for social policy*. This helped introduce the researchers to the topic, as many had not previously worked in this area. As all were voluntary research collaborators no IRB approval was necessary for this study.

The main task they were given was to design their own test of the same hypothesis ("Main Analysis" in Fig S1). They were to first design their test (analogous to a pre-analysis plan) and then after submitting this, to engage in the research. To simulate a real-world research environment there were of course free to deviate from this plan if they felt it necessary (see Communication S8. Main analysis). The communications verbatim are available in the Supplementary Material Appendix 'Communications'.

Participant Materials

We provided the ISSP data in comma separated and Stata formats, and if teams requested it, we converted them to any other format. We provided three measures of immigrant "stock" from the World Bank, United Nations (UN) and Organization for Economic Co-Operation and Development (OECD) and one measure of immigrant "flow" as net migration provided by the UN. These were all data sources we could find that covered a majority of the countries in the ISSP data. We provided a variety of macro-indicators of GDP per capita, Gini, Social Spending, Employment Rate, Unemployment Rate, Population and Ethnic-Fractionalization following our knowledge of variables commonly used in the state of the art in this research area compiled into one comma-separated file (also a Stata file available) to make it easier for the participants and to reduce choices they had to make. See folder "Provided Materials" in the "data" folder in our Project Repository and see instructions in Communication S6. Research design. The specific questions from the ISSP that participants were asked to use for testing the hypothesis came from a battery (English verbatim here):

"On the whole, do you think it should or should not be the government's responsibility to ...

- ... provide a job for everyone who wants one
- ... provide health care for the sick
- ... provide a decent standard of living for the old
- ... provide a decent standard of living for the unemployed
- ... reduce income differences between the rich and the poor

- ... provide decent housing for those who can't afford it”

For each question respondents could select from “Definitely should be”, “Probably should be”, “Probably should not be”, “Definitely should not be”, and “Can’t choose”. Throughout our code and occasionally in the Supplementary Materials we refer to the variables produced from these questions as *Jobs, Health*, *OldAge, Unemp, IncDiff* and *House*.

Researcher Characteristics

Participant Survey

We speculated that researchers’ own qualities might influence their replications, research designs and expansion results. Therefore, we surveyed researchers on both objective criteria, such as experience with methods and the substantive topic, and subjective criteria, such as their own beliefs about the hypothesis and immigration in general(5). In addition, we asked them questions about their time commitment, constraints they faced and some other feedback about the process of crowdsourcing. We conducted 4 waves throughout the CRI allowing us to field a core questionnaire and determine if participation in the CRI or either experimental condition might alter subjective perceptions and experiences, see Supplementary Materials Appendix III. Participant Survey Codebook for more details and all frequency distributions.

Table S1 provides a subset of researcher characteristics aggregated to the team level. Our teams contained 1-3 researchers who on average disagreed or were neutral in their beliefs that the hypothesis that immigration reduces support for social policy is true, felt that restrictions on immigration should be lower in their countries, have taught roughly 2-9 undergraduate statistics courses, were interested in the topic of the CRI, and had at least one if not more immigration and social policy topic related publications.

Survey Question	Mean	SD	Min	Max
Hypothesis is False, Wave 1	2.4	0.6	1	5
Hypothesis is False, Wave 2	2.5	0.6	1	5
Less Immigration Restrictions Preferable	4.5	1.1	1	6
Stats Teaching (# of courses)	5.4	3.7	1	12
Multilevel Modeling Skill	4.1	0.8	1	5
Stats Publications	1.9	0.8	1	3
Topic Interest	4.1	1	1	5
Immigration-Topic Publications	2.1	0.9	1	3
Policy-Topic Publications	1.7	0.9	1	3

Table S1. Selected researcher characteristics

Based on the Participant Survey (First wave N = 162 researchers, final wave N = 161), values aggregated to the team-level. See III. Participant Survey Codebook for all questions. Publications

questions coded (0=none, 1=one, 2= more than one). Created by repository file 002_CRI_Data_Prep

Measurement of Characteristics

We constructed our survey to tap into specific characteristics of researchers. In particular, we were interested in their prior beliefs about the main hypothesis, attitudes toward immigration, statistical skills and prior topical experience and knowledge (correlations in Table 1). We used structural equation modelling techniques to generate multi-item scales that offer a reliable metric for comparison. There are many more items in our survey beyond these characteristics and we hope future researchers will engage with these rich data (available on the Harvard Dataverse and our Project Repository) (*5*).

We engaged in measurement techniques at both the individual-level and aggregated team-level and we found that in either case our reflective indicators correlated at 0.95 at the team-level so we opted for the aggregate team-level approach. We could only use data at the team-level as teams submitted results jointly and there is no variance across team-members within-teams on either of our main dependent variables of model effects and subjective conclusions about the hypothesis.

Table S2 provides correlations of all variables used and Fig S3 provides the measurement model and fit indices and the chi-square/df p-value using those variables.

survey question	label	stat1	stat2	stat3	stat4	stat5	stat6	stat7	topic1	topic2	topic3	topic4	topic5
Method Skill, Constraint	(stat1)												
Software Skill, Constraint	(stat2)	0.28											
First Phase Difficulty	(stat3)	0.43	0.08										
Stats Teaching Experience	(stat4)	0.39	-0.06	0.30									
Multilevel Modelling Experience	(stat5)	0.56	0.00	0.32	0.47								
Stats-Related Publications	(stat6)	0.22	-0.02	0.23	0.48	0.41							
Multilevel Publications	(stat7)	0.35	-0.01	0.15	0.47	0.55	0.43						
Immigration-topic Publications	(topic1)	0.30	0.07	0.12	0.29	0.37	0.17	0.24					
Policy-related Publications	(topic2)	0.29	0.11	0.18	0.37	0.37	0.32	0.41	0.43				
Opinion-Policy Publications	(topic3)	0.26	-0.04	0.12	0.46	0.30	0.33	0.28	0.26	0.33			
Interest in Topic	(topic4)	0.00	-0.03	0.03	0.07	0.15	0.03	0.17	0.27	0.14	0.25		
Not Familiar with Topic	(topic5)	0.17	-0.01	0.22	-0.05	0.21	-0.03	0.10	0.32	0.20	0.26	0.26	
Very Familiar with Topic	(topic6)	0.20	-0.08	0.13	0.12	0.21	0.07	0.09	0.26	0.29	0.40	0.23	0.27
Topic-related Publications	(topic7)	0.16	0.09	0.07	0.31	0.19	0.08	0.12	0.26	0.34	0.26	0.14	0.15
Topical Teaching Experience	(topic8)	0.13	0.11	0.12	0.18	0.20	0.13	0.12	0.32	0.43	0.22	0.15	0.18
Topic-related Dicussions	(topic9)	0.13	0.02	0.26	0.18	0.29	0.14	0.12	0.17	0.13	0.33	0.12	0.23
Belief in Hypothesis General	(belief1)	0.00	0.06	-0.01	0.12	0.12	0.05	0.00	0.18	0.03	-0.02	-0.13	-0.11
Belief in H, Old-age	(belief2)	0.10	0.02	0.14	-0.01	-0.06	0.17	0.03	0.07	-0.04	0.07	-0.14	-0.19
Belief in H, Unemployment	(belief3)	0.04	0.10	-0.01	0.02	0.10	-0.02	-0.16	0.15	-0.02	-0.14	-0.20	-0.16
Belief in H, Income Gap	(belief4)	-0.16	-0.02	-0.07	-0.02	-0.02	0.02	-0.09	0.18	0.06	0.02	-0.13	-0.09
Belief in H, Housing	(belief5)	-0.09	-0.16	0.05	0.20	0.24	-0.01	0.12	0.26	0.18	-0.04	0.02	0.00
Belief in H, Job Provision	(belief6)	-0.28	0.05	-0.05	-0.18	-0.15	-0.05	-0.28	0.07	-0.09	0.01	-0.08	0.10
Belief in H, Health Care	(belief7)	-0.22	-0.20	-0.09	0.14	0.02	-0.07	0.09	0.13	0.18	0.03	0.03	-0.02
Strength of Belief in H	(belief_strength)	0.25	0.14	0.11	0.10	0.24	0.12	0.25	0.34	0.23	0.26	0.18	0.38
Shouldn't Restrict Immigration	(pro_immigrant)	0.22	-0.08	0.18	0.07	0.18	-0.05	0.09	0.13	0.03	0.01	0.03	0.15

	label	topic6	topic7	topic8	topic9	belief1	belief2	belief3	belief4	belief5	belief6	belief7	belief_strength
Very Familiar with Topic	(topic6)												
Topic-related Publications	(topic7)	0.38											
Topical Teaching Experience	(topic8)	0.27	0.59										
Topic-related Dicussions	(topic9)	0.06	0.26	0.15									
Belief in Hypothesis General	(belief1)	-0.22	0.11	0.03	0.02								
Belief in H, Old-age	(belief2)	0.10	0.05	0.16	-0.11	0.17							
Belief in H, Unemployment	(belief3)	0.10	0.07	0.03	-0.14	0.52	0.19						
Belief in H, Income Gap	(belief4)	-0.07	-0.03	-0.18	0.04	0.59	-0.04	0.53					
Belief in H, Housing	(belief5)	-0.09	0.06	0.11	0.04	0.56	0.07	0.45	0.48				
Belief in H, Job Provision	(belief6)	0.11	0.00	-0.05	-0.07	0.44	0.05	0.46	0.53	0.29			
Belief in H, Health Care	(belief7)	0.01	0.05	0.03	-0.10	0.45	0.16	0.21	0.29	0.57	0.24		
Strength of Belief in H	(belief_strength)	0.19	0.08	0.11	0.08	-0.22	-0.11	-0.41	-0.28	-0.24	-0.04	-0.09	
Shouldn't Restrict Immigration	(pro_immigrant)	0.00	0.00	-0.05	0.04	0.01	-0.12	0.06	-0.04	0.09	-0.16	-0.02	-0.02

Table S2. Correlations among researcher characteristics

Values aggregated to the team-level and then correlated. N=161 Participants in 73 Teams. Created by repository file 002_CRI_Data_Prep

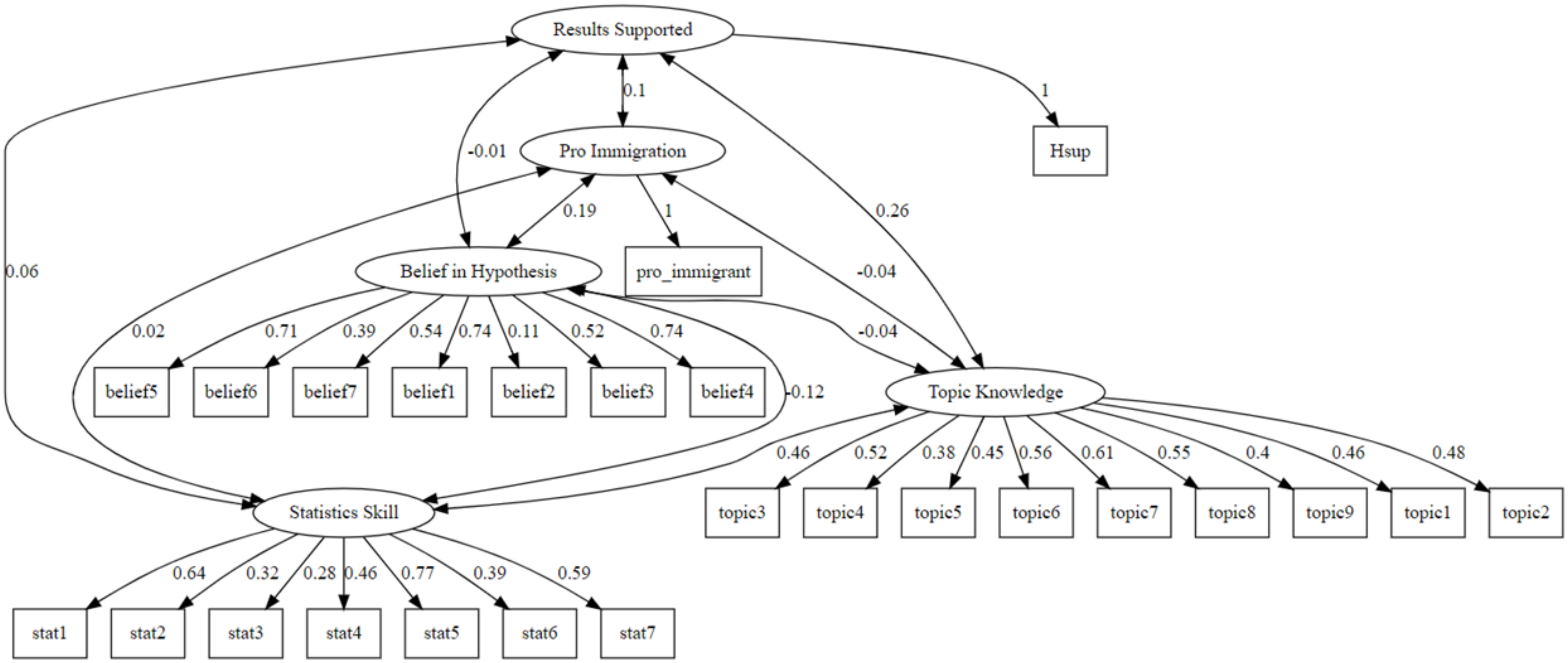

Fig S3. Measurement model for researcher characteristics

Team-Level, N = 73. CLI= 0.855, TFI= 0.837, RMSEA=0.061, chi/df p= 0.004. A similar analysis was conducted at the participant level yielding results correlated at 0.95. Created by [Project Repository]() file 002_CRI_Data_Prep.Rmd.

Model Ranking

There were two opportunities for participants to rank the veracity of given models and their specific features. In the fourth wave of the survey, we randomly but systematically submitted the descriptions of the models from the researcher's pre-analysis plans. We also asked participants to rank certain characteristics of models during an online deliberation.

IV. Model Ranking during Participant Survey.

The survey response rate led to 3-4 rankings per model. This allowed us to calculate the average score per model in response to the question of "…how confident [the Participant was] that the respective research design is adequate for testing the hypothesis that 'immigration undermines social policy preferences' using ISSP data" on a scale from 1 to 5 covering response categories "Unconfident", "Rather unconfident", "Neither unconfident nor confident", "Rather confident" and "Confident."

Model aspect ranking during deliberation

We collected descriptive evidence on the deliberation process as it unfolded using an online deliberation platform Kialo. We selected an online communication platform that was developed using scientific insight with deliberative principles in mind(4). We asked users to rank the veracity of four statements regarding case selection, the use of robust clustered standard errors, the power to detect an effect of the test variable and the use of what some refer to as a "two-way fixed effects" (2WFE) model. These four categories emerged from the pre-analysis plans submitted by each team regarding their ideal test models, and based on a preliminary deliberation regarding research designs randomly assigned to half the teams. Reference to these features were mentioned in all designs and many teams directly challenged the state of the art reliance on rich western democracies, failures to account for units nested in higher levels and the shortcomings of the 2WFE approach.

As many participants elected not to participate in the post-results deliberation (see Fig S2. Teams and Participants by Stage) we asked them to vote by email and added their votes to the final tallies.

Combined model scoring

We then took the ranking of each component of the models from the participant survey and the deliberation voting and centered these so that the average rank of the usage of OLS regression as an estimation strategy would equal zero. Thus, we have relative rankings (displayed in Fig S4 below.). We shorten the three negative bars for easier visualization. The coding procedure takes place in the R file 'CRI/data_prep/001_CRI_Subj_Votes.Rmd' in our Project Repository.

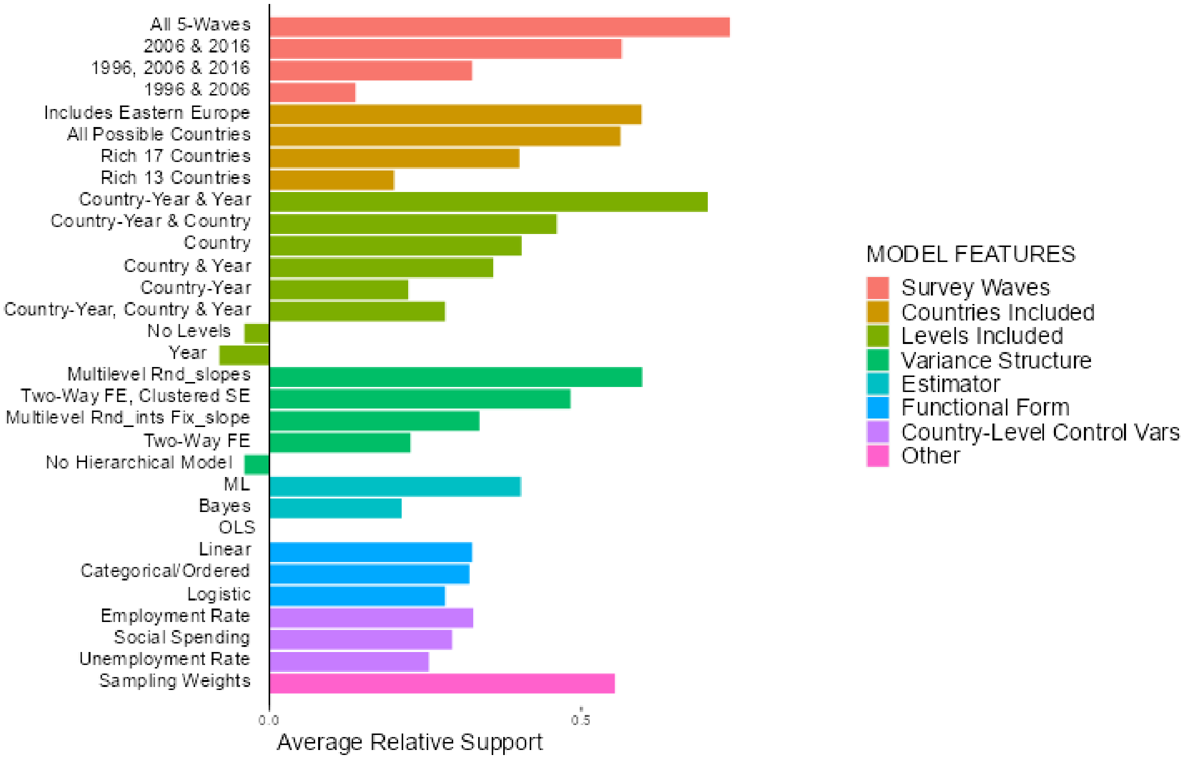

Fig S4. Subjective ranking of model specifications

Scores standardized and centered at the mean score for OLS. Three negative bars shortened for ease of visualization. Scores determined by peer review. Each participant ranked 4-5 models and these scores were compiled and combined with a voting system used in an online deliberation to create an average score for all models that contained a given listed component above, see preceding section for more details (see

IV. Model Ranking during Participant Survey) . Created by repository file 001_CRI_Subj_Votes.

Team Results

Effects and Conclusions

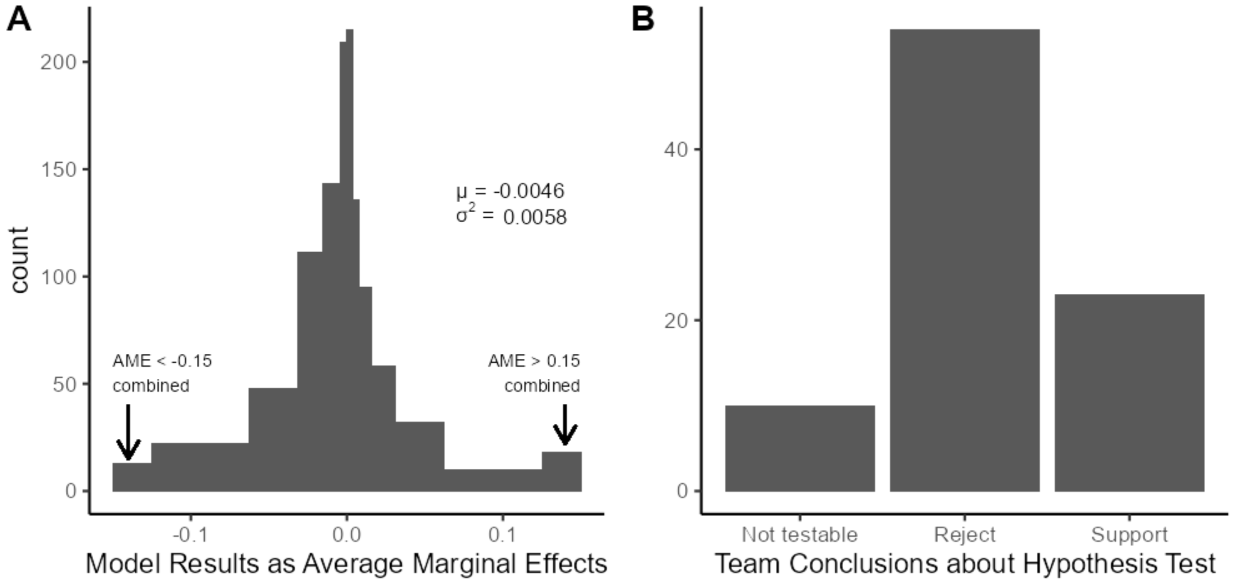

Fig S5. Distribution of effect sizes and subjective conclusions

Average Marginal Effects ("AME") are standardized across models and between immigration test variables to maximize comparability. Everything beyond AME +/- 0.15 are trimmed into the outermost bins. Bin size is quadratic-decreasing. Mean and variance are weighted by the number of models per team. In Panel B, there are 88 subjective conclusions from 73 teams, because some teams drew different conclusions for the two main test variables measuring immigration. Created by repository file 01_CRI_Descriptives.

Model Specification Coding

We used qualitative coding of each team's pre-analysis plan to derive a basic outline of model specifications. However, the teams reported vastly different aspects of models, some teams code did not match their planned or reported models and in the end many teams deviated from their original plans. Therefore, model specifications come from a careful review of each team's submitted code. All specifications that were found in at least three team's models were coded in addition to any 'major' specifications such as the inclusion of additional variables, alterations to the regression equation or any sample selection procedures made. Some specifications were too detailed to include. Whereas roughly 3% of models involved a Bayesian estimation technique to recover credible intervals, it was too detailed to report exact features of the Bayesian models relating to the burn-in, iterations and posterior updating criteria. Therefore, these few models were simply coded as having a "Bayes" estimator. The same is true for models including a measure of fractionalization. Although some teams used an ethnic-fractionalization measure and others used a religious- or cultural-fractionalization measure, we simply coded any type of measure under the general code "fractionalization" as an independent variable. A list of all model specifications and their coding rules and frequency distributions are available in Supplementary Materials Appendix II. Model Specification Coding and Distribution.

Each team's code is available publicly in the Project Repository.

Value	N	Cum. %	Weight. %
Dependent Variable			
IncDiff	205	16.26%	12.53%
Jobs	205	16.26%	12.53%
OldAge	205	16.26%	12.53%
Unemp	205	16.26%	12.53%
Multi-item scale	195	15.46%	35.67%
Health	123	9.75%	7.11%
House	123	9.75%	7.11%
total		100.00%	100.00%
Test Variable, immigration measure			
Stock	625	49.56%	51.39%
Flow	593	47.03%	45.14%
Change in Flow	43	3.41%	3.47%
total		100.00%	100.00%
Test Variable, lag			
current	622	49.33%	51.74%
1-year	473	37.51%	34.78%
per wave	63	5.00%	5.90%
5-year	60	4.76%	5.03%
10-year	27	2.14%	1.85%
3-year	16	1.27%	0.69%
total		100.00%	100.00%
Test Variable, measurement			
Immigrant, foreign-born	727	57.65%	59.63%
Net Migration	424	33.62%	35.97%
Non-Western Immigrant	36	2.85%	1.27%
Western Immigrant	36	2.85%	1.27%
Muslim-country Immigrant	18	1.43%	0.46%
Refugee	16	1.27%	0.69%
Asylum applicants	4	0.32%	0.69%
total		100.00%	100.00%
Test Variable, multiple in model			
No (Independent test)	843	66.85%	69.28%
Yes	418	33.15%	30.72%
total		100.00%	100.00%

Value	N	Cum. %	Weight. %
Software			
Stata	894	70.90%	59.72%
R	241	19.11%	26.39%
Mplus	74	5.87%	9.72%
MLwiN	32	2.54%	2.78%
SPSS	20	1.59%	1.39%
total		100.00%	100.00%
Estimator			
Logistic	630	49.96%	36.81%
General linear model	398	31.56%	32.87%
Ordinary least squares	118	9.36%	15.05%
Ordered/Multinomial logistic	82	6.50%	7.64%
Bayesian	33	2.62%	7.64%
total		100.00%	100.00%
Number of Countries in Sample			
13	327	25.93%	31.28%
26	156	12.37%	4.17%
11	123	9.75%	11.62%
14	88	6.98%	2.66%
12	85	6.74%	3.21%
15	82	6.50%	7.82%
22	73	5.79%	3.94%
17	56	4.44%	6.71%
16	47	3.73%	4.77%
27	47	3.73%	2.95%
25	40	3.17%	2.66%
24	30	2.38%	2.20%
10	18	1.43%	1.85%
18	16	1.27%	1.74%
other	73	5.79%	12.41%
total		100.00%	100.00%
Country-Level Independent Variables			
Social Spending	512	40.60%	51.60%
Other/None	441	34.97%	28.72%
Employment Rate	188	14.91%	8.15%
Unemployment Rate	76	6.03%	4.79%
GDP per capita	44	3.49%	6.75%
total		100.00%	100.00%

Table S3. Selected frequencies of model specifications out of 1,261 models

Codebook for available in II. Model Specification Coding and Distribution. Created by repository file 002_CRI_Data_Prep.

Common Models

No two models were identical when comparing all specifications. However, looking across sub-domains we see that some models had identical samples, variables or estimation/equation components (below in Table S4. Common model specifications by sub-domain clusters. We expected that these sub-domains should be among the most important decisions when testing the hypothesis. "Identical" may not be completely identical, for example inclusion of a GDP per capita indicator at the country-time level would be coded regardless of how GDP was measured (e.g., whether this came from the United Nations, World Bank or OECD data, or whether it was lagged by one year or not) because these details are not common enough to get their own codes.

Specifications by Sub-Domain	AME (mean)	AME (sd)	No. of Identical Models	No. of Teams	Support Hypothesis (mean)
Sample Specifications					
13 Richest Democracies, 1996 & 2006 Waves	0.018	0.174	189	9	0.167
11 of 13 Richest Democracies, 1996, 2006 & 2016 Waves	-0.026	0.098	102	8	0.375
13 Richest Democracies, 1996, 2006 & 2016 Waves	-0.002	0.056	96	10	0.050
Variable Specifications					
No country-level, and 5 socio-demographic individual-level indep. variables	-0.001	0.012	53	6	0.509
No country-level, and 5 socio-demographic individual-level indep. variables + income	-0.002	0.039	38	7	0.135
Estimation/Equation Specifications					
DV: Jobs, Test var: Stock of immigrants, logistic regression, 'two-way fixed effects'	0.005	0.004	18	8	0.000
DV: Unemployment, Test var: Stock of immigrants, logistic regression, 'two-way fixed effects'	-0.003	0.007	18	8	0.000
DV: Income Differences, Test var: Stock of immigrants, logistic regression, 'two-way fixed effects'	0.000	0.013	18	8	0.000
DV: Old-age Care, Test var: Stock of immigrants, logistic regression, 'two-way fixed effects'	0.006	0.011	18	8	0.000
DV: House, Test var: Stock of immigrants, logistic regression, 'two-way fixed effects'	0.004	0.003	17	10	0.244
DV: Health, Test var: Stock of immigrants, logistic regression, 'two-way fixed effects'	-0.008	0.015	17	10	0.244
DV: Jobs, Test var: Net migration, logistic regression, 'two-way fixed effects'	0.006	0.016	17	10	0.244
DV: Unemployment, Test var: Net migration, logistic regression, 'two-way fixed effects'	-0.009	0.005	17	10	0.244
DV: Income Differences, Test var: Net migration, logistic regression, 'two-way fixed effects'	-0.002	0.003	11	8	0.043
DV: Old-age Care, Test var: Net migration, logistic regression, 'two-way fixed effects'	0.006	0.008	11	8	0.043
DV: House, Test var: Net migration, logistic regression, 'two-way fixed effects'	0.007	0.007	9	6	0.000
DV: Health, Test var: Net migration, logistic regression, 'two-way fixed effects'	0.007	0.012	9	6	0.000

Table S4. Common model specifications by sub-domain clusters

Weighted means and sd by number of models per team. "Identical models" indicates identical specifications within each sub-domain. Of all 1,532 models none were identical when comparing across all domains. Created by repository file 02_Common_Specifications.

Explaining Result Variance

Visualizing Result Variance

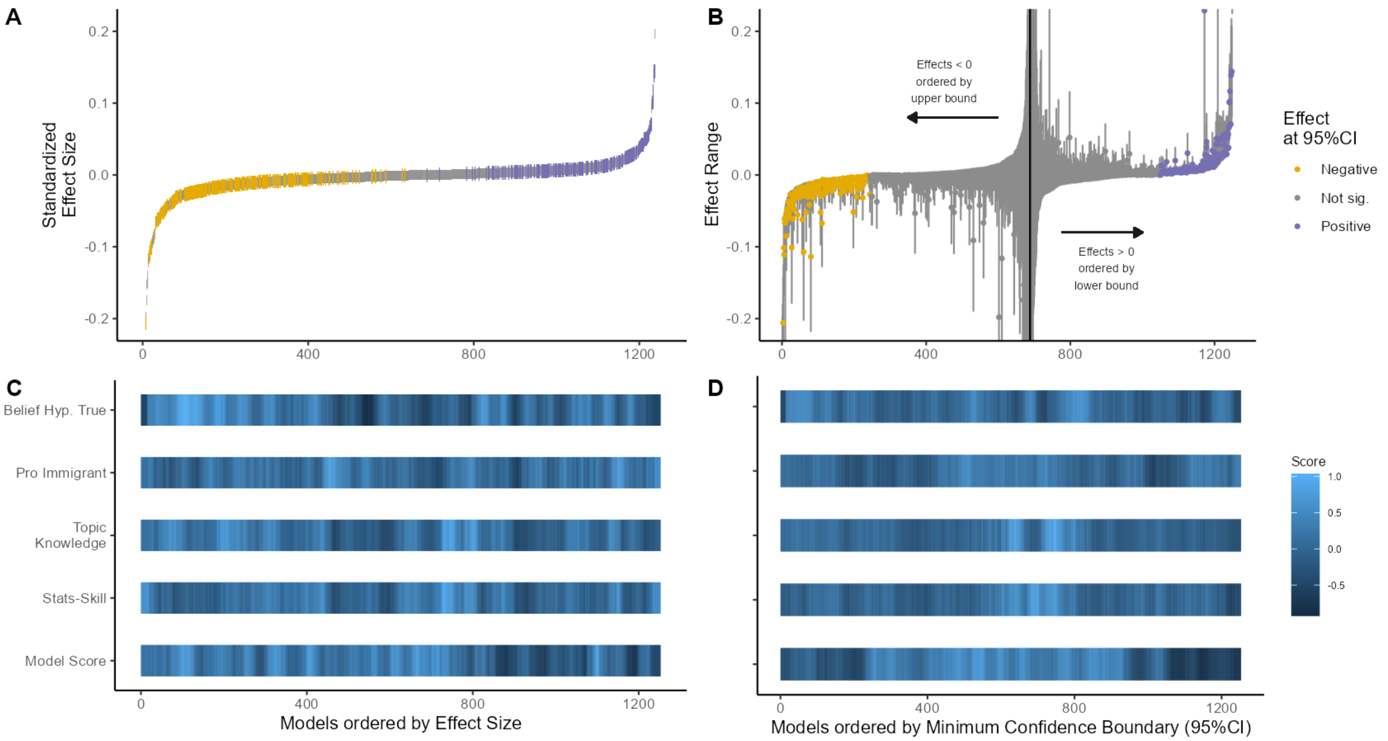

Fig S6. Specification curves by researcher characteristics and model score
Specification curves ordered by AMEs (left) and minimum value of the 95% confidence interval (right) compared to a 10-model rolling average of researcher characteristics, used to help identify potential patterns. Created by repository file 03_CRI_Spec_Analysis.

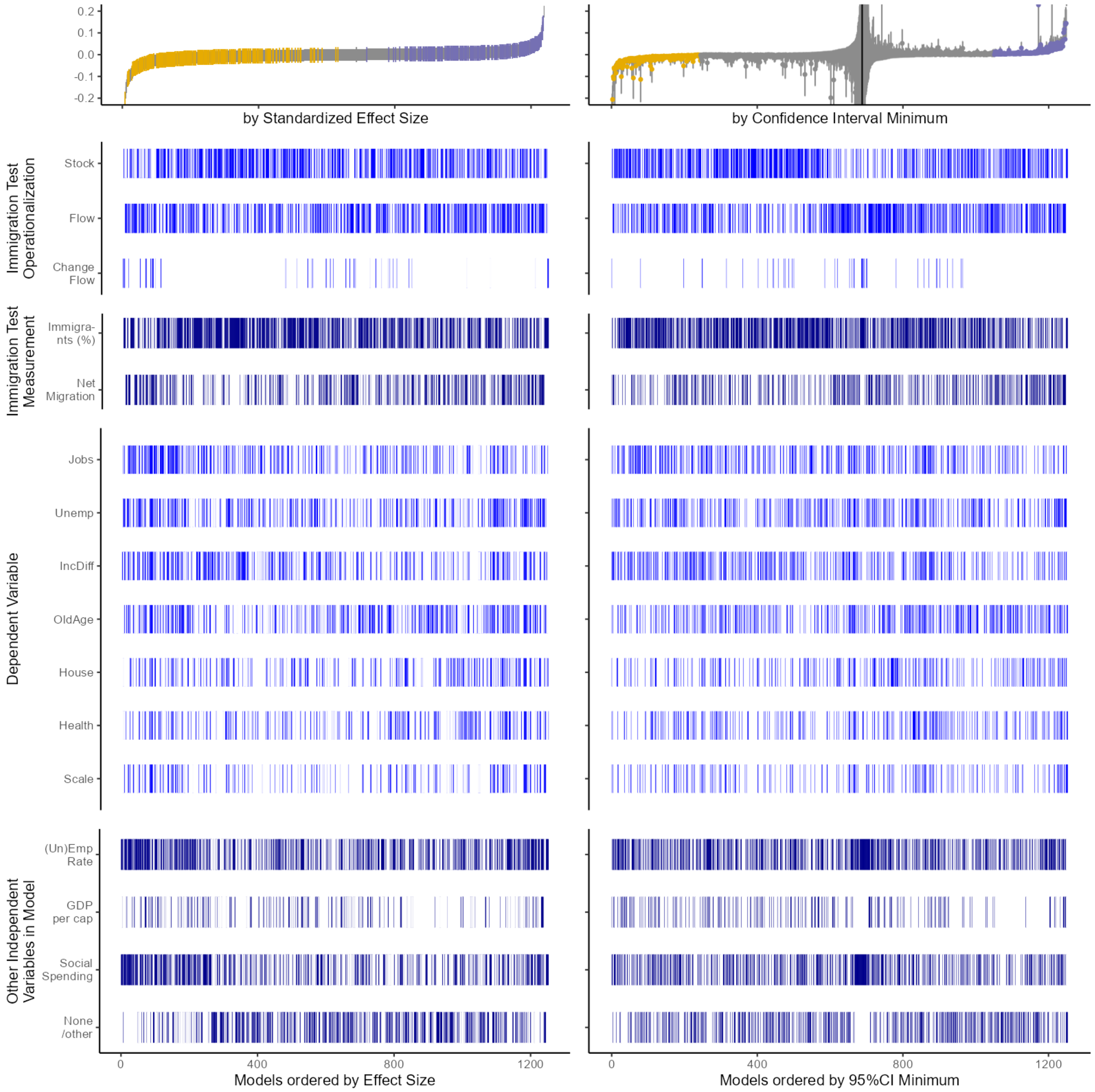

Fig S7. Specification curves from Fig S6 plotted against measurement decisions

Created by repository file 03_CRI_Spec_Analysis.

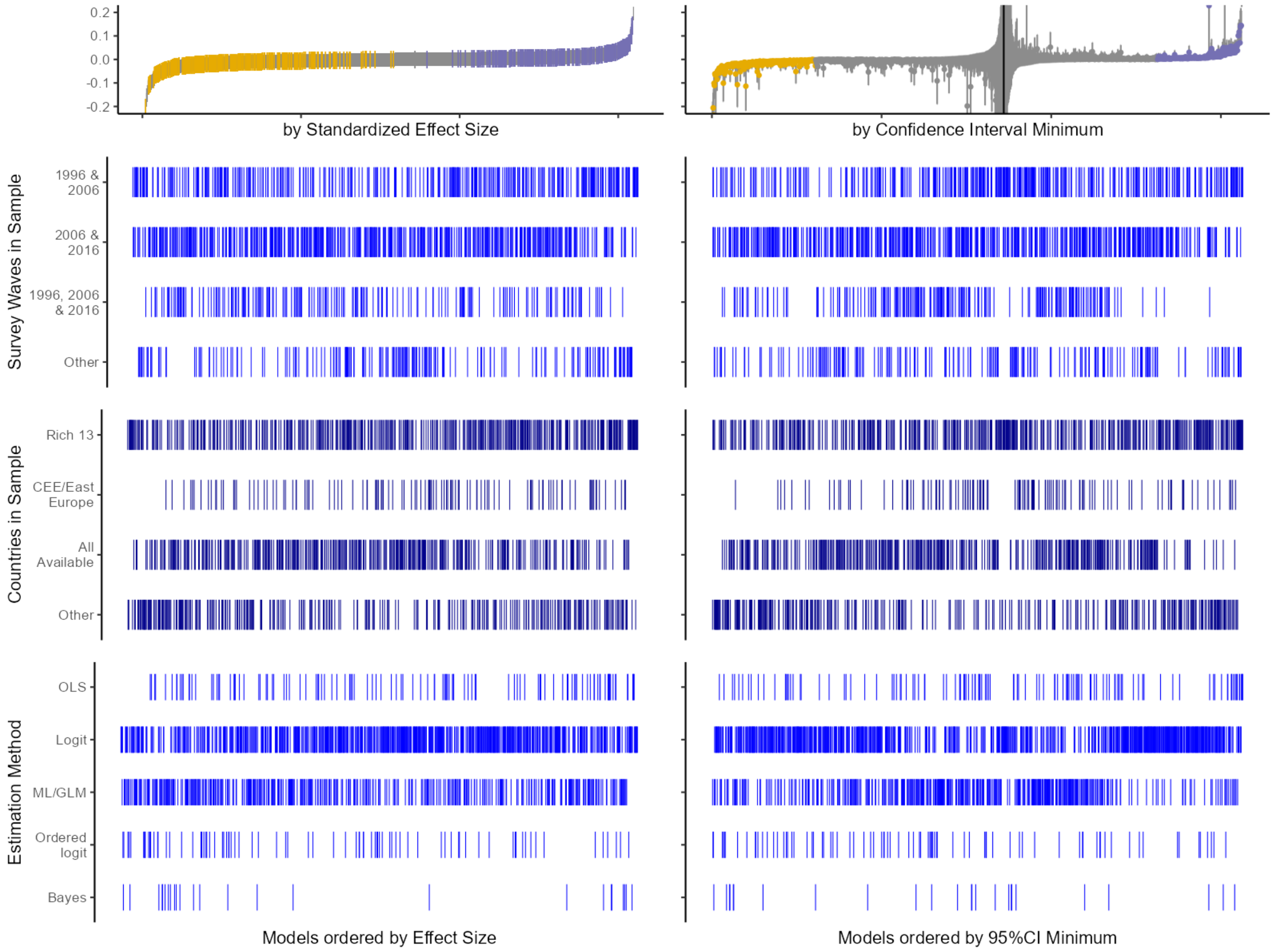

Fig S8. Specification curves from Fig S6 plotted against sample and estimation methods
Created by repository file 03_CRI_Spec_Analysis.

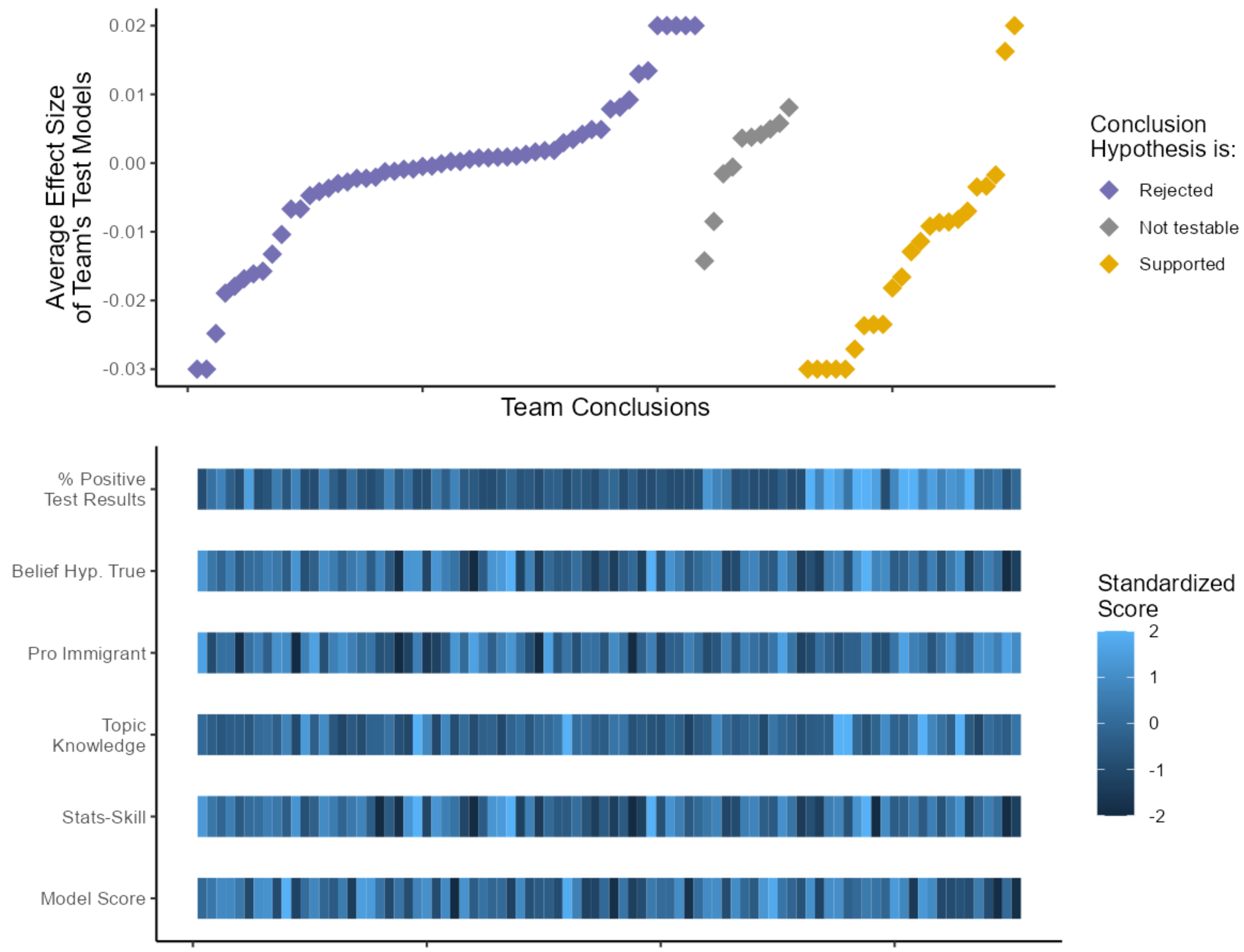

Fig S9. Subjective conclusions by team plotted against researcher characteristics
Teams reported their subjective conclusions as either "Supported" or "Rejected" the hypothesis, or that they believed the hypothesis was "Not testable" given these data. The Y-axis presents the average AME by team, X-axis only used to correlate conclusions (above) with model specifications (below). We find that 14.8% of team-findings had a majority of negative effects (supporting the hypothesis that immigration reduces support for social policy) that were significant at $p<0.05$, while 6.8% had a majority of positive effects (contradicting the hypothesis). Overall, 13.5% (12/89) concluded "Not Testable" (one team not shown here because they concluded it was not testable before running their models due to a failed measurement invariance test), 60.7% (54/89) concluded "Reject" and 25.8% (23/89) concluded "Support". Created by repository file 03_CRI_Spec_Analysis.

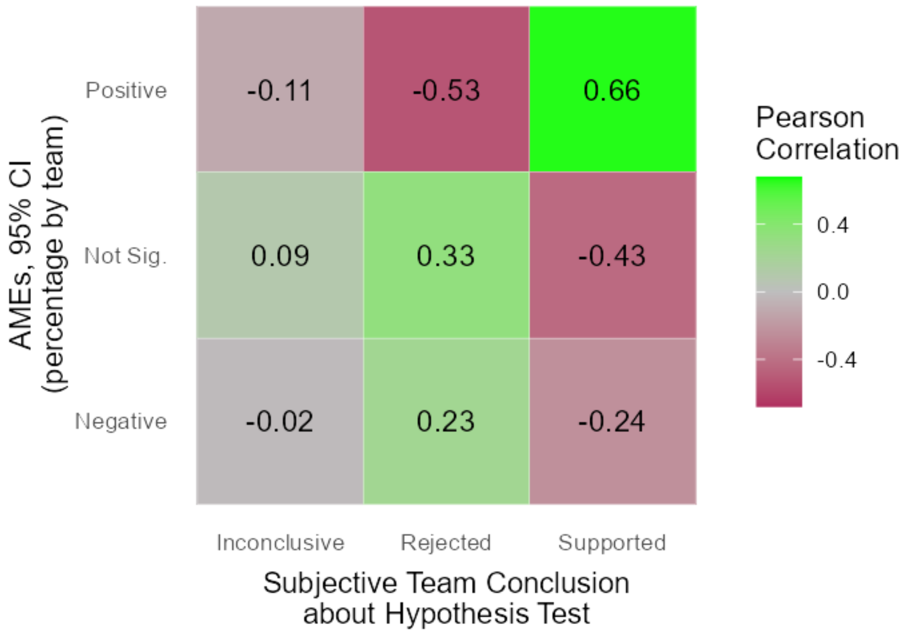

Fig S10. Subjective conclusions by percentage of supporting test results
Pearson correlation heat map plotting the three outcomes as dummy variables compared with the percentage of AMEs per team that were not significantly different from zero for the "Inconclusive" column, positive and significantly different from zero for the "Rejected" column and negative and significantly different from zero for the "Supported" column; all at 95%CI. Created by repository file 01_CRI_Descriptives.

Main Regression Models

We develop regression models to explain the Average Marginal Effect (AME) estimates in each model using the following formula.

$$Y_{mt} = \alpha + X_m + Z_t + \mu_{mt} + y_t \quad (1)$$

In formula (1) Y is the AME for a given model m produced by team t regressed on a grand-mean intercept α, a vector of model specification variables X_m, a vector of team-level variables Z_t, a model-specific error term μ_{mt} and a team-specific error term (the deviance from the grand mean intercept for each team, a team-specific intercept) y_t. Standard two-level multi-level model using general least squares (also known as "hierarchical linear model" in some literatures). The results of models adding in variables for the dependent variable used in a given team's model, the estimator, the main test variable, sample, model design and researcher characteristics are added to the regression and the reduction in the variance of μ_{mt} ("Within-team variance" in Figure 3, main text) and y_t ("Between-team variance") is presented as a percentage to indicate how much each model specification can explain at each level.

Predictors	m00	m01	m02	m04	m06	m07	m09	m10	m11	m12	m13
(Intercept)	-0.002	0.002	0.003	-0.006	-0.037	-0.040	-0.030	-0.029	-0.030	-0.023	-0.030
DV (ref = all others)											
Jobs		-0.008	-0.008	-0.007	-0.007	-0.006	-0.007	-0.007	-0.007	-0.007	-0.007
Income-Differences		-0.012**	-0.013**	-0.012*	-0.012*	-0.012*	-0.012*	-0.012*	-0.012*	-0.012*	-0.012*
Housing		0.014 *	0.014 *	0.013 *	0.013 *	0.013 *	0.012 *	0.012 *	0.012 *	0.013 *	0.012 *
Exp Group 1			-0.004								
Exp Group 2			0.001								
Estimation (ref = all others)											
Logit				0.017 *	0.016 *	0.018 *	0.017 *	0.017 *	0.018 *	0.017 *	0.017 *
OLS				0.014	0.020	0.026 *	0.024 *	0.024 *	0.024 *	0.023	0.024
Main Test Measure (ref = Immigration Flow)											
Test: Immigration Stock				-0.005	-0.005	-0.005	-0.005	-0.005	-0.005	-0.005	-0.005
Test: Change in Flow				0.043 ***	0.047 ***	0.049 ***	0.048 ***	0.048 ***	0.048 ***	0.048 ***	0.048 ***
Sample Includes:											
1996 Wave					-0.005	-0.015	-0.016	-0.016	-0.016	-0.019	-0.016
2006 Wave					0.031	0.021	0.020	0.020	0.019	0.016	0.020
2016 Wave					0.036	0.028	0.025	0.025	0.025	0.020	0.025
2006*2016 Interaction					-0.043	-0.039	-0.037	-0.037	-0.037	-0.033	-0.037
13 Rich Democracies					0.015	0.019 *	0.018 *	0.018	0.018 *	0.018	0.018 *
Eastern Europe (at least 3 countries)					0.025 *	0.028 *	0.027 *	0.027 *	0.027 *	0.026 *	0.027 *
All Available Countries					-0.022	-0.020	-0.019	-0.019	-0.019	-0.018	-0.019
Model Design											
"Two-Way Fixed-Effects"						0.021	0.017	0.017	0.017	0.018 *	0.016
Country-Year Level						0.023 *	0.025 *	0.026 *	0.026 *	0.024 *	0.025 *
Fixed-Effect (within unit test)						0.005					
Random-Effect (between unit test)						0.008					
Researcher Characteristics											
Belief in Hypothesis							0.000				
Pro-Immigrant								0.000			
Topical Knowledge									0.001		
Stats Experience										0.010	
Model Score											0.000

*Confidence interval, does not cross zero at * 95%   ** 99%   *** 99.9%*

Table S5. Multilevel linear regression predicting variance in AME by model and team
Created by repository file 04_CRI_Main_Analyses.

	m00	m01	m02	m04	m06	m07	m09	m10	m11	m12	m13
*Residual Variance (*100)*											
Team-Level	0.062	0.060	0.062	0.055	0.053	0.053	0.053	0.053	0.053	0.052	0.052
Model-Level	0.488	0.484	0.485	0.480	0.478	0.477	0.477	0.477	0.477	0.476	0.477
Total	0.550	0.545	0.547	0.535	0.531	0.530	0.530	0.529	0.530	0.529	0.529
Variance Explained											
Team-Level	0	2.3%	-1.1%	11.2%	13.8%	13.5%	14.1%	14.3%	14.1%	15.3%	15.1%
Model-Level	0	0.8%	0.8%	1.7%	2.0%	2.3%	2.4%	2.4%	2.4%	2.4%	2.3%
Total	0	0.9%	0.5%	2.8%	3.4%	3.6%	3.7%	3.7%	3.7%	3.9%	**3.8%**
Model Indicators											
AIC	-3024	-3005	-2985	-2985	-2932	-2903	-2911	-2908	-2911	-2911	-2914
log-Likelihood	1514.8	1508.5	1500.6	1502.4	1482.9	1472.3	1475.2	1474.1	1475.5	1475.6	1476.8
Team-N/ Model-N	87/ 1,253	87/ 1,253	87/ 1,253	87/ 1,253	87/ 1,253	87/ 1,253	87/ 1,253	87/ 1,253	87/ 1,253	87/ 1,253	87/ 1,253

Table S6. Model statistics for Table S5
Created by repository file 04_CRI_Main_Analyses.

Panel A. The Likelihood of Hypothesis Test Conclusion "Support" versus "Not testable"						
Variable	m00_H (Odds-ratio)	m01_H (Odds-ratio)	m02_H (Odds-ratio)	m04_H (Odds-ratio)	m06_H (Odds-ratio)	m08_H (Odds-ratio)
(Intercept)	-0.738 *	-0.562	-0.400	0.439	0.331	-2.155
Dependent Variable (ref = others)						
Jobs		-0.214	-0.154	13.418	13.881	15.801 ***
IncDiff		-0.034	-0.221	-13.580	-13.720	-14.463 ***
House		-0.182	0.068	-0.038	-0.055	
Exp Group 1			-0.331			
Exp Group 2			-0.054			
Estimator (ref = others)						
Logistic				0.569	1.143	1.223
Ordinary Least Squares				0.787	1.295	3.968
Test Variable (ref = "Flow")						
Stock				-2.010	-2.137	-2.530
Change in Flow				-83.068 *	-99.463	-272.542 ***
Sample Includes:						
2006 Wave					-2.003	-3.172
2016 Wave					1.518	2.306
13 Rich Democracies					1.000	3.288 *
Eastern Europe					3.942	7.245
All Available Countries					-3.653	-4.038
Model Design:						
"Two-Way Fixed-Effects"						1.648
Includes Country-Year Level						2.589
Within Country Slopes						75.697 ***
Non-linearities						-2.215

*Confidence interval, does not cross zero at * 95% ** 99% *** 99.9%*

Table S7. Multinomial regression predicting support for hypothesis

Continued on next page. Created from 04_CRI_Main_Analyses.

Panel B. The Likelihood of Hypothesis Test Conclusion "Support" versus "Reject"

	m00_H	m01_H	m02_H	m04_H	m06_H	m08_H
(Intercept)	0.853 ***	1.293 **	1.704 **	2.731 **	1.746	2.397
Dependent Variable (ref = others)						
Jobs		-0.276	-0.208	-0.112	-0.165	-1.012
IncDiff		-1.232	-1.439	-1.566	-1.257	0.378
House		0.955	1.231	1.067	1.336	
Research Conditions						
Experimental Group			-0.253			
Deliberation Group			-0.653			
Estimator (ref = others)						
Logistic				0.437	0.760	0.739
Ordinary Least Squares				0.375	0.529	1.210
Test Variable (ref = "Flow")						
Stock				-2.669 **	-2.659 **	-3.072 **
Change in Flow				-2.710	-2.188	-143.892 ***
Sample Includes:						
2006 Wave					-0.456	-0.847
2016 Wave					0.653	1.156
13 Rich Democracies					1.234	1.245
Eastern Europe					3.372	4.183
All Available Countries					-2.720	-3.154
Model Design:						
"Two-Way Fixed-Effects"						-0.167
Includes Country-Year Level						0.562
Within Country Slopes						71.839 ***
Non-linearities						-0.256
**Panel C. Model Statistics**						
Observations	88	88	88	88	88	88
R2 Nagelkerke	0	0.048	0.077	0.214	0.287	0.515
Deviance	160.215	156.574	154.329	142.79	136.006	110.479
log-Likelihood	-80.107	-78.287	-77.165	-71.395	-68.003	-55.24

*Confidence interval, does not cross zero at * 95% ** 99% *** 99.9%*

Table S7 [continued]. Multinomial regression predicting support for hypothesis

Panel A. The Likelihood of Hypothesis Test Conclusion "Support" versus "Not testable"							
Variable	m00_H (Odds-ratio)	m09_H (Odds-ratio)	m10_H (Odds-ratio)	m11_H (Odds-ratio)	m12_H (Odds-ratio)	m13_H (Odds-ratio)	m14_H (Odds-ratio)
(Intercept)	-0.738 *	-2.041	-0.261	-4.112	-3.202	-4.576	-2.484
Dependent Variable (ref = others)							
Jobs		16.378 ***	16.385 ***	21.206 ***	16.518 ***	10.23	23.045 ***
Income-Differences		-15.324 ***	-14.987 ***	-18.717 ***	-14.563 ***	-9.493	-20.871 ***
Estimator (ref = others)							
Logistic		1.034	1.237	1.151	1.452	1.443	0.879
Ordinary Least Squares		3.976	4.306 *	3.15	4.015	4.692 *	3.723
Test Variable (ref = "Flow")							
Stock		-2.592	-2.384	-2.305	-2.417	-2.474	-2.29
Change in Flow		-300.010 ***	-263.162 ***	-234.357 ***	-261.068 ***	-200.864 ***	-227.623 ***
Sample Includes:							
2006 Wave		-3.213	-3.788	-1.859	-2.79	-3.522	-2.617
2016 Wave		2.644	2.364	2.309	2.394	1.923	2.815
13 Rich Democracies		3.017	3.683 *	3.675 *	3.532 *	3.381	3.829 *
Eastern Europe		7.15	7.233	9.018 *	7.458	6.612	9.215 *
All Available Countries		-4.06	-3.736	-5.969	-4.203	-4.242	-6.14
Model Design:							
"Two-Way Fixed-Effects"		1.794	1.766	1.452	1.646	1.686	1.899
Includes Country-Year Level		2.492	2.818	2.412	2.878	2.245	2.565
Within Country Slopes		87.046 ***	71.190 ***	48.702 ***	65.790 ***	56.587 ***	39.689 ***
Includes GDP measure		-127.350 ***	-96.073 ***	-87.926 ***	-105.332 ***	-69.768 ***	-77.693 ***
Non-linearities		-2.129	-2.613	-1.977	-2.423	-1.709	-2.219
Researcher Characteristics							
Belief Hypothesis is True		-1.505				-1.975	1.194
Pro-Immigration			-0.408			-0.113	-1.287
Topic Knowledge				-4.914		-11.859	-7.528
Stats Skills					-1.088	2.006	0.515
Model Score						18.842	
Results: % Positive at CI95							-10.076*
Results: % Neg. at CI95							0.280

*Confidence interval, does not cross zero at * 95% ** 99% *** 99.9%*

Table S7 [continued].

Panel B. The Likelihood of Hypothesis Test Conclusion "Support" versus "Reject"	m00_H	m09_H	m10_H	m11_H	m12_H	m13_H	m14_H
(Intercept)	0.853 ***	2.391	4.805	1.414	2.216	1.955	4.287
Dependent Variable (ref = others)							
Jobs		-1.024	-1.067	-0.439	-0.992	-1.03	-0.535
Income-Differences		0.406	0.502	0.155	0.465	0.313	-0.045
Estimator (ref = others)							
Logistic		0.741	0.677	0.687	0.781	0.808	0.461
Ordinary Least Squares		1.202	1.566	0.835	1.13	1.454	1.331
Test Variable (ref = "Flow")							
Stock		-3.086 **	-2.929 **	-2.902 **	-3.036 **	-3.064 **	-2.886 **
Change in Flow		-166.789 ***	-134.444 ***	-88.460 ***	-123.676 ***	-107.292 ***	-68.342 ***
Sample Includes:							
2006 Wave		-0.841	-1.516	-0.228	-0.799	-1.108	-0.893
2016 Wave		1.168	1.231	1.316	1.208	1.078	1.249
13 Rich Democracies		1.254	1.719	1.412	1.307	1.235	1.857
Eastern Europe		4.208	4.285	5.842	4.172	4.016	6.235
All Available Countries		-3.19	-2.91	-4.974	-3.191	-3.205	-5.003
Model Design:							
"Two-Way Fixed-Effects"		-0.183	-0.033	-0.128	-0.163	-0.089	0.092
Includes Country-Year Level		0.582	0.808	0.535	0.615	0.587	0.723
Within Country Slopes		83.249 ***	67.360 ***	43.819 ***	61.579 ***	53.384 ***	34.530 ***
Includes GDP measure		-2.128 *	-2.091 *	-2.020 *	-2.096 *	-2.112 *	-1.951 *
Non-linearities		-0.263	-0.718	-0.324	-0.292	-0.202	-0.803
Researcher Characteristics							
Belief Hypothesis is True		-0.053				0.167	3.536
Pro-Immigration			-0.526			-0.514	-1.576*
Topic Knowledge				-2.589		-3.455	-4.156
Stats Skills					-0.249	1.030	
Model Score						2.339	
Results: % Positive at CI95							
Results: % Neg. at CI95							-11.120**
**Panel C. Model Statistics**							0.884
Observations	88	88	88	88	88	88	88
R2 Nagelkerke	0	0.522	0.536	0.544	0.521	0.599	0.762
Deviance	160.215	109.632	107.674	106.587	109.721	98.929	70.667
log-Likelihood	-80.107	-54.816	-53.837	-53.294	-54.86	-49.465	-35.334

*Confidence interval, does not cross zero at * 95% ** 99% *** 99.9%*

Table S7 [continued].

significantly different from zero at 95%CI.

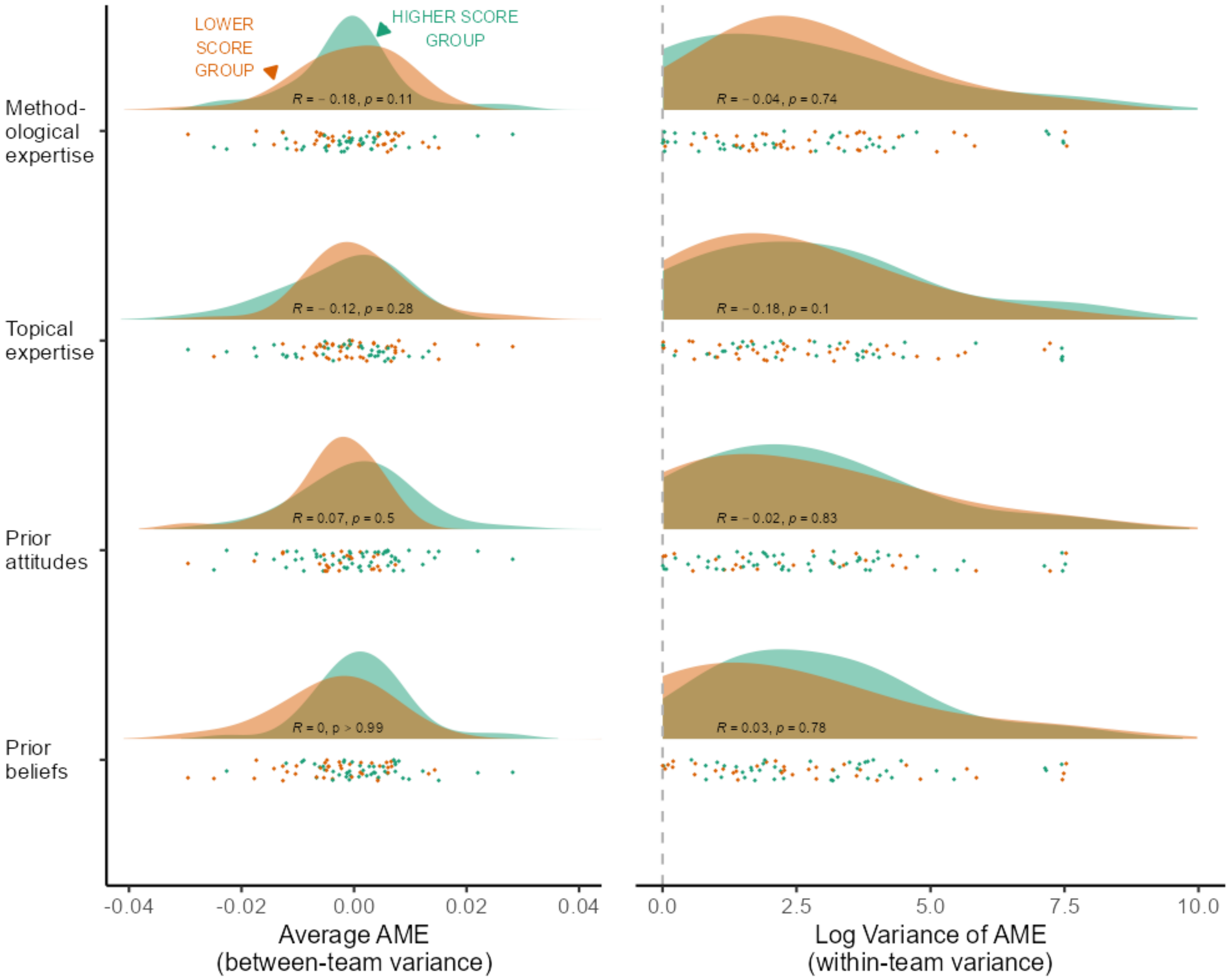

Fig S11. Unexplained team and model-level variance by researcher characteristics

Similar to Fig 2 in the main text, but plotting the intercept and within-team variance unexplained by regression model M7. Created from repository file 05_CRI_Main_Analyses_Variance_Function.

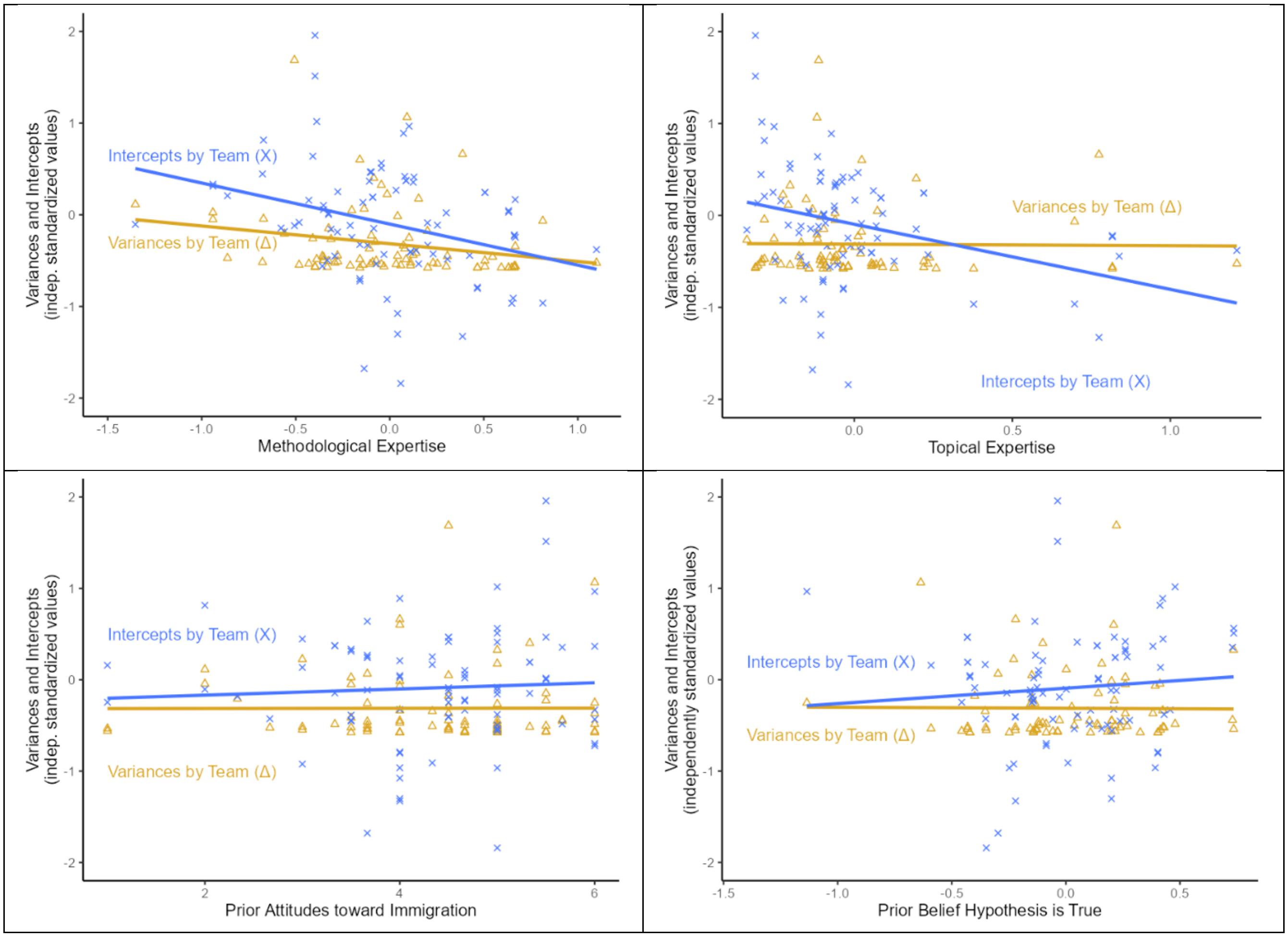

Fig S12. Intercept and unexplained variance by researcher characteristics
Intercepts and residual within-team variance from a reduced version of M7. These are the linear versions of the R-squared values presented in Fig S11. All correlations not significant at $p > 0.05$. Created from repository file [05_CRI_Main_Analyses_Variance_Function].

Multiverse Analysis – How Much Variance Should we Expect to Explain?

It is possible that the 3.8% of total variance in AMEs we can explain through model specifications and researcher characteristics is not surprisingly low. We have low variance and results go in all directions, and perhaps 3.8% is reasonable. We test this by simulating model specifications on a dataset worked up by one of the teams – Team 18, the first team going in order from 1 upwards we found that used R and three waves of data in their sample. They used dichotomized dependent variables and logit regressions, so we had to adjust the code slightly to accommodate linear dependent variables as well. We then simulated all these potentially real researcher model specifications based on the common model specifications from our study. After running all six dependent variables across 3 different sample specifications, two different types of immigration measures, up to four independent variables at the country level, and three different estimation techniques with various variance components by year and country we had 2,304 models. The code 05_CRI_Multiverse.Rmd details this process in our Project Repository. Table S8 below demonstrates the explained variance using the r-squared statistic. As we did not have teams in this simulation we can only explain the total variance. As a reminder to the reader, we could explain 3.8% of the total variance in the real results submitted by the teams (see Table S6 above).

Model	Specifications	r^2
m1	DVs + AME type	0.118
m2	+ sample	0.119
m3	+ IVs	0.128
m4	+ other DV + Estimator	0.131
m5	+ DV*Type interaction	0.164

Table S8. Multiverse results explaining simulated researcher variability

We ran 2,304 models simulating all possible choices among major model specifications used by the teams. Then we predicted the resulting AMEs using the model specifications. The r-squared reveals how much variance we can explain, and we use this as a baseline to compare against our results. Created from repository file 06_CRI_Multiverse.

DV-Specific Regression Models

Model ID	DV	Model Specifications Included	r2	adj_r2	Models
m03	Jobs	~ logit + ologit + lpm + mlogit + ols	0.012	-0.013	203
m03	Unemp		0.008	-0.017	203
m03	IncDiff		0.016	-0.009	203
m03	OldAge		0.021	-0.004	203
m03	House		0.010	-0.033	123
m03	Health		0.014	-0.028	123
m04	Jobs	~ logit + ols + Stock + ChangeFlow	0.204	0.188	203
m04	Unemp		0.049	0.030	203
m04	IncDiff		0.122	0.104	203
m04	OldAge		0.146	0.129	203
m04	House		0.312	0.289	123
m04	Health		0.307	0.284	123
m06	Jobs	~ logit + ols + Stock + ChangeFlow + w1996 + w2006 + w2016 + w2006*w2016 + orig13 + eeurope + allavailable	0.226	0.182	203
m06	Unemp		0.101	0.049	203
m06	IncDiff		0.161	0.112	203
m06	OldAge		0.172	0.124	203
m06	House		0.356	0.298	123
m06	Health		0.319	0.258	123
m08x	Jobs	~ logit + ols + Stock + ChangeFlow + twowayfe + mlm_fe + mlm_re + gdp_ivC + anynonlin	0.221	0.180	203
m08x	Unemp		0.067	0.018	203
m08x	IncDiff		0.181	0.139	203
m08x	OldAge		0.183	0.140	203
m08x	House		0.452	0.403	123
m08x	Health		0.394	0.339	123
m12x	Jobs	~ logit + ols + Stock + ChangeFlow + twowayfe + mlm_fe + belief_ipred + pro_immigrant + stats_ipred + topic_ipred + total_score	0.216	0.171	203
m12x	Unemp		0.091	0.039	203
m12x	IncDiff		0.183	0.136	203
m12x	OldAge		0.214	0.169	203
m12x	House		0.470	0.417	123
m12x	Health		0.397	0.337	123

Table S9. DV-specific regressions predicting AMEs
Linear regressions with model specifications as independent variables and AME as dependent variable were run separately for each dependent variable of the six to which the teams were limited. House and Health used less frequently across teams, thus the smaller number of models. Model ID is analogous to the IDs used in Table S5. Main Regression Results, except those with “x” referring to the removal of some variables to maximize the adjusted r-squared.

Dredging Variance Function

Predictors	m13 (Tbl S5)	Auto_1	Auto_1_m13	Auto_2	Auto_3	Auto_4	Auto_5
(Intercept)	-0.030	-0.006	-0.016	-0.006	-0.011	-0.005	0.002
DV (ref = all others)							
Jobs	-0.007	-0.011	-0.010	-0.011	-0.011	-0.011	-0.011
IncDiff	-0.012 *	-0.025 ***	-0.024 ***	-0.025 ***	-0.025 ***	-0.025 ***	-0.025 ***
House	0.012 *	0.014 *	0.013 *	0.014 *	0.014 *	0.015 **	0.014 *
Unemp						-0.004	
Estimation (ref = all others)							
Logit	0.017 *		0.017 *				
OLS	0.024		0.020		0.012		
Main Test Measure (ref = Flow)							
Test: Immigration Stock	-0.005	-0.019 **	-0.020 **	-0.019 **	-0.019 **	-0.019 **	-0.020 **
Test: Change in Flow	0.048 ***	0.045 ***	0.048 ***	0.045 ***	0.043 ***	0.045 ***	0.044 ***
Sample Includes:							
1996 Wave	-0.016		-0.017				-0.016
2006 Wave	0.020		0.019				
2016 Wave	0.025	-0.007	0.026	-0.006	-0.007	-0.007	-0.010
2006*2016 Interaction	-0.037		-0.037				
13 Rich Democracies	0.018 *	0.018	0.018 *	0.018	0.018	0.018	0.020 *
Eastern Europe	0.027 *	0.012	0.028 *	0.013	0.012	0.012	0.015
All Available Countries	-0.019		-0.020				
Model Design							
"Two-Way Fixed Effects"	0.016	0.006	0.003	0.007	0.006	0.006	0.014
Country-Year Level	0.025 *	0.017	0.025 *	0.017	0.014	0.017	0.026 *
Any Clustering				-0.006			
Unbalanced Panel					0.010		
GDP as Indep. Variable				-0.005			
Conservatism as Indep. Variable							0.035
Public Employment as Indep. Variable						-0.002	

*Confidence interval, does not cross zero at * 95% ** 99% *** 99.9%*

Table S10. Computer-assisted variance explained

Using a 'dredge' function we ran all possible combinations of the variables previously determined to have any potential to explain variance (see Table S5) (6). We first iterate through types of model specifications to reduce computing time and avoid over-fitting (see Auto_1). Then we create a list of all possible interactions, then remove those that have no variance, and then run 1,000 samples of a random 7 interaction terms leading to over 1 million models. From these we select the best two fitting models from each of the 1,000 runs and make a list of all variables from those runs. We then iterate over 100 samples of random 7 of these variables and take the best fitting two models from each of the 100 leading to four potential interaction variables that might improve the baseline model m13 (from Table S5 and leading to Auto_2-Auto_5). Sometimes the AIC score is improved and sometimes the level-specific variance explained as well, but none can explain as much of the total variance. Created from repository file 06_CRI_Multiverse.

Predictors	m13 (Tbl S5)	Auto_1	Auto_1_m13	Auto_2	Auto_3	Auto_4	Auto_5
Algorithm Probed Interactions							
IncDiff * Stock		0.026 **	0.026 **	0.026 **	0.025 **	0.025 **	0.026 **
Jobs * "Two-Way Fixed.."		0.012	0.011	0.012	0.011	0.012	0.012
Stock * "Two-Way Fixed.."		0.016	0.017	0.016	0.016	0.016	0.016
Jobs * Stock		-0.002	-0.003	-0.002	-0.003	-0.003	-0.003
Any Clustering * GDP				-0.006			
OLS * Unbalanced Pan.					-0.021		
Unemp * Public Emp.						0.000	
1996 Wave * Conservatism							-0.047
Model Score	0.000		-0.009				
Model Statistics							
Residual Variance							
Team-Level	0.001	0.001	0.000	0.001	0.001	0.001	0.001
Model-Level	0.005	0.005	0.005	0.005	0.005	0.005	0.005
Total	0.005	0.005	0.005	0.005	0.005	0.005	0.005
Explained Variance							
Team-Level	15.13%	12.20%	19.68%	7.00%	10.74%	9.11%	6.67%
Model-Level	2.34%	2.63%	2.89%	2.67%	2.59%	2.57%	2.97%
Total	**3.77%**	**3.70%**	**4.77%**	**3.15%**	**3.50%**	**3.30%**	**3.39%**
Team-Test Level	87	87	87	87	87	87	87
Model-Level	1,253	1,253	1,253	1,253	1,253	1,253	1,253
AIC	-2913.8	-2935.2	-2888.3	-2909.4	-2910.4	-2909.4	-2915.7
log-Likelihood	1476.9	1484.6	1468.2	1474.7	1475.2	1474.7	1477.8

*Confidence interval, does not cross zero at * 95%   ** 99%   *** 99.9%*

Table S10 continued. Computer-assisted variance explained

See table caption above for explanation

Variance Function Regression

Considering the multilevel model presented in formula (1) we can use a variance function regression with the same within- and between-team-variables to estimate their statistical associations with residual variance also decomposed to the within- and between-levels using the following notation(*32*)

$$\hat{Y}_{mt} = \boldsymbol{R'_{mt}\beta} \tag{2}$$

$$\log \sigma^2_{mt} = \boldsymbol{S'_{mt}\lambda} \tag{3}$$

Formula (2) is a short-hand for formula (1) and β is a coefficient associated with each given variable in the vector R'_{mt} (which includes both X and Z vectors from that formula). Together they give the predicted value $\hat{Y}_{mt}$ for each model in each team. Formula (3) represents the unexplained variance σ^2_{mt} in the residuals $\mu_{mt} + y_t$ (which together equal $\hat{Y}_{mt} - Y_{mt}$) after estimating formula (2). It is logged following a standard procedure of model fitting, or what many know as detecting heteroscedasticity. But here, the difference in a one-unit log change is predicted as a result of one-unit changes in each of the vector of variables S'_{mt} which may or may not be identical to those in R'_{mt}. The coefficient of this prediction for each variable is λ. The result is that the X and Z vectors of variables can be used to explain not only higher or lower values of Y, but also the degree of unpredictability in Y for each team, i.e., the residual variance.

To calculate these shorthand formulas (2) and (3) we

1. Calculate the total residuals $\mu_{mt} + y_t$ and all regression coefficients β from formula (1).
2. Fit a gamma regression with a log-link of the squared residuals on S'_{mt} to get all coefficients λ. Then save fitted values $\sigma^2_{mt} = \exp\,(S'_{mt}\,\lambda)$.
3. Fit a weighted linear regression of Y_{mt} on R'_{mt} where the weights are $\frac{1}{\alpha^2_{mt}}$.
4. Iterate steps 2 and 3 to convergence, updating β and predicted values of $\mu_{mt} + y_t$ from the linear regression and λ and σ^2_{mt} from the gamma regression. This means β is now corrected for any heteroscedasticity in $\hat{Y}_{mt}$.

The results of this regression using Model m07 but the model cannot converge because these are far too many predictors for the variance function. Therefore, we elect to only use our researcher characteristics variables Belief in Hypothesis, Pro-Immigrant, Topical Knowledge, Stats Experience and Model Score. This is an extremely liberal test as it considers no model specifications, thus if we do not observe any meaningful variance predictions here, we can almost entirely rule out these variables as meaningful to the degree (variance) of result variance by team.

Variable	Variance Coeff	Mean Coeff	Standard Coeff
Belief in Hypothesis	0.354	-0.005	-0.002
Pro-Immigrant	0.006	0.000	0.000
Topical Knowledge	-0.566	-0.038	-0.010
Stats Experience	0.200	0.028	0.006
Model Score	3.722	-0.023	-0.005

Table S11. Variance function regression results

No coefficients β or λ are significantly different from zero at 99%CI. “Mean Coeff” is the β now corrected for any heteroscedasticity in $\hat{Y}_{mt}$, whereas “Standard Coeff” is the normal regression result without correction. Created in repository file 05_CRI_Main_Analyses_Variance_Function.

Supplementary Material Appendix

I. Communications

Become one among many authors:

Replicate and enhance a cross-national quantitative study

One way of making social science more open is to move beyond individual or small research teams working in isolation, and use modern technology to scale up and crowdsource. This component of the MZES Open Social Science Conference 2019 promotes and explores the practice of crowdsourcing in social science studies.

For the OSSC19 Crowdsourced Replication Initiative, we seek researchers to participate in a crowdsourced replication project on a high-profile social science question: **How does immigration shape public opinion?**

Besides the theoretical and practical importance of this question, the crowdsourced replication project is relevant to social science for two reasons. One is that research on this topic is thus far **inconclusive**. The other is a need to explore the epistemic **potential of crowdsourcing approaches** in macro-comparative social research.

Crowdsourcing is a methodologically innovative means for answering research questions using secondary data. Crowdsourcing replication, expansion, deliberation, experimental practices, and meta-analyses may provide substantial improvements in substantive and methodological findings over what any research team could achieve alone. With this initiative, we aim to explore and develop crowdsourcing as a methodology for the social sciences.

Participation in the project helps promote this goal. Specifically, participating researchers will (a) **replicate** and (b) **expand** a previously published **cross-national quantitative study**. We plan to distribute the results of this collective effort through the Open Science Framework (OSF) and prepare them for publication in a high visibility social science journal. **All participants who complete the analytical tasks will be co-authors on the final paper**, written under the stewardship of Dr. Nate Breznau.

We invite teams of 1-3 researchers to **independently analyze the data**. Team members should have solid knowledge in and prior **experience with quantitative statistical analysis**. The data provided for analysis by the conference co-organizers are both individual and country-level; therefore knowledge of or willingness to engage in **macro-comparative research**, i.e., identifying effects at ‘level-2’, is desirable. Researchers from all disciplines are welcome to participate, at all levels of research, including professors, postdocs, PhD students, lecturers, and analysts working in non-academic professions. The amount of time needed to participate for each team will depend on their methodological skills. The conference co-organizers will **provide all necessary data**. We estimate the first part of the project, the replication of the original study, will take no more than **six to eight hours** on average. The second part of the project, the expansion of the study with new data, will depend on the individual goals of the participating researchers in improving upon the original study but is likely to take a **few days of work**.

Researchers should **apply** to take part in the OSSC19 Crowdsourced Replication Initiative by filling out the following form no later than 27 July 2018. In August we will distribute data and instructions. Replications should be completed by 23 September 2018 and expansions by 19 November 2018. Each team’s models and results will be made public in the bundle of all results, but will not be identifiable to any other team or the public. Irrespective of the ‘quality’ of research or ‘capabilities’ of researchers, each team’s participation makes a **positive contribution** to developing crowdsourcing as a specific open social science practice and our specific crowdsourced initiative.

Please contact Dr. Nate Breznau with any inquiries you may have regarding the OSSC19 Crowdsourced Replication Initiative.

Communication S1. Call for Researchers
The original text including bolded words and phrases, as it appeared on the OSSC19 website from 20/6/2018 until 27/7/2018. Excludes original links and website graphics. The call was publicized across academic networks, email listserv’s and social media.

Thank you for your participation in the OSSC19 Crowdsourced Replication Initiative. This research project investigates the topic of immigration and social policy attitudes. Your participation will extend the scientific understanding of this topic and help develop crowdsourcing and replication as social science research methods with the particular goal of overcoming some of the problems inherent in research done alone or in a single research team.

Let us first express our gratitude and excitement about the high number of researchers who registered for this project and have thus shown their support for this goal. We are looking forward to exploring with you ways to improve how we as social scientists conduct our research.

As part of this project, you are both a researcher and a participant in an experiment. As a researcher, you will use macro-comparative, cross-national survey data to investigate the linkages between immigration and social policy preferences. As a participant, the Initiative PI's will assign you to one of several groups with varying research conditions, in order to examine how to best do crowdsourced replication work.

During this process we expect you to follow the instructions you will receive to the best of your ability. This will be necessary to allow for a well-coordinated crowdsourcing effort. As you fulfil these tasks, you will replicate one or more high impact studies related to this topic. Following the replication, you will plan and conduct an expansion of the original study, in addition to deliberating with other research teams. Please be aware of the following two points:

(1) If you participate in this project and complete the tasks, you will be a co-author of the paper presenting the substantive results of our effort. The paper is currently planned as part of a special issue on open science in a visible publication outlet. By guaranteeing authorship we hope to remove incentives for p-hacking and other unethical research practices and only offer incentives for you to pursue knowledge and truth. No incentive to find something statistically significant, positive, negative, or otherwise.

(2) It is essential that you do not discuss or share the information, instructions, and activities contained in this project while it is ongoing as this could bias the experiment and study results.

This project will proceed in several steps, including study replication, study expansion and intermittent deliberations between participants. These tasks will be carried out between the end of August the beginning of January.
You will receive replication data and a first survey invitation on *August 20th*. Replication results will be due on *September 10th*. Research designs for the expansion analyses will be due on *September 23rd*. The expansion analysis will be due on *November 19th*. On *January 5th* we will draw joint conclusions about the project results.

Please be mindful of these dates in your own planning. We will be in touch in due course regarding a more detailed timeline of the initiative.

It is essential that you complete your tasks associated with the crowdsourced initiative on time. This is necessary in order to accomplish a well-coordinated replication enterprise. If you are unable to complete your tasks or unsure, please contact us immediately. Researchers or teams that are unable to meet the deadlines will have to be considered dropouts, including the loss of authorship, to preserve the experimental conditions and to keep this large-scale project manageable.

We are excited about this project and we are grateful for your participation.
If you have any questions please address Nate Breznau at this email address.

Communication S2. Welcome information, 7/28/2020
Email sent to all who registered to participate.

It is time to begin the OSSC19 Crowdsourced Replication Initiative (CRI) replication stage. First we need all members of your team to complete a short (~2 min.) survey. Shortly thereafter you will receive your replication data and instructions.

Please click the following link to begin the survey:

#code_complete#

Note that this is a personalized link. Each individual in the survey received a personalized link. Please do not share. If you lose the link or it does not work please request a new one. It is possible to interrupt filling out the survey and to continue later if you need to.

You will be asked to respond to occasional surveys during the CRI as part of your research tasks. If you participate with a research team, each member of your team should fill out each survey independent of the other members. Please do not discuss your responses to the survey questions in your team.

Keep in mind that September 10, 2018 is the deadline for the replication results, which is why we ask that you complete this short survey within two working days. At the end of this email you will find the overall CRI timetable, to give you an overview of the several stages of this project.

Thank you again for your participation in this innovative research collaboration.

Communication S3. Wave 1 survey invitation, 8/20/2018

In this project we are crowdsourcing the replication of a 2014 study by Brady and Finnigan (B&F). The published paper and online supplemental materials are attached to this email and in a shared folder (see link below). There are many different types of replication. Your team has only one goal in this first stage of replication. That is to replicate this study to determine *verifiability*. You are to assess whether the reported results of the study follow appropriately from the data and methods employed by the original authors.

We provide you with the same two waves of *International Social Survey Program* (ISSP) data, the country-level data, and the analytical code (*Stata* format) used by B&F, and you should follow their reported methods as closely as possible to determine if:

1. their results are reproducible - to check their results;
2. the results you find (whether identical or not) confirm their reported conclusions; and
3. the methods they describe in their paper are accurately reflected in their models and results - to check their work.

We ask that you replicate their work using your preferred statistical software. That is the software that your team plans to work in throughout this entire project. This is important because there are many more stages that will build on the code you develop in this stage, and we do not expect you to learn new code for this project. We ask you to assess verifiability of their methods and results, and to do this independently of their *Stata* code (.do file), although you are welcome to use it as a guide or run it to cross-check your own code. Please do everything that the authors reported doing in executing their analyses. However, it is not necessary to run their supplemental models or analyses for now. At a minimum we ask that you replicate the results from Tables 4 and 5. If you like you can replicate other models, but we need your verifiability test for Tables 4 and 5 - otherwise the replication will be incomplete.

To ensure that you use the correct version of the ISSP data, download these datafiles from our shared data folder (they are too large to attach to an email), they are in either *Stata*, .csv, or .xls format and titled ZA2900 and ZA4700. Note that in .csv and .xls format the data contain no meta-data (i.e., no variable labels or differentiation between string and numeric) so you might need access to additional documentation. If you cannot manage to import or work with one of these formats please contact us for transferring the data into your preferred format.

Shared Data Folder (click to access ISSP data, plus other materials left here for convenience; if you do not have HTML enabled email you may copy and paste the link at the end of this email into your browser).

Please be sure that you document all your work and that we can reproduce your results using the code you give us. Please document any cases in which you conclude that the authors' research is not verifiable in either results or the match between what they claim to do and what they actually do (i.e., points 1-3 above). Please write a short summary of your arguments supporting claims that their reported methods *do not match* their actual methods. If during this replication concerns or ideas arise for different or better analytical strategies than those employed by the original authors, this is great, but please keep them in mind for the phase after the replication when you will be asked to expand or improve upon this particular study. But for now, we ask that you do not yet run additional analyses or alternative model specifications as these might bias your task.

Results should be submitted by September 10th, 2018 to [Nate Breznau] and must include your code saved in its own language file (e.g., .do, .R, .inp, etc) and a results table in spreadsheet format. We provide an attached Excel Replication Template where you can fill in your results for B&F's Tables 4 and 5, but feel free to replicate their other main models if you are interested. It is not necessary to reproduce or verify their graphs for now.

We know how much time pressure you may face as a productive scholar, but we must stress the importance of completing the replication on time as the success of the project depends on starting the next phase of the CRI on time. We estimate that this exercise may take between 5 and 14 hours of working time depending very much on your own experience with the data and/or the models employed herein. Thank you for your understanding and participation in this exciting initiative. We remind you that all participants completing the CRI tasks will be co-authors on the final paper where we present the results of the study. Do not hesitate to ask if you have questions or need assistance.

Communication S4a. Replication instructions for Transparent Group, 23/8/2018

Participant teams were randomly assigned to one of two groups. This group was given the original study's published article and publicly shared Stata code.

You are now asked to replicate a study to start this project. You are assigned to replicate a published study but to do so without knowing the study. We realize this may seem unusual; however, your participation is crucially important to developing deeper knowledge about replication and crowdsourcing. We kindly ask that you attempt to replicate this study to the best of your ability using only the materials we provide, and without spending time trying to ‘figure out’ where it came from. Again, your cooperation in this collaborative and co-authored research project is of great importance.

Attached to this email is a Methods and Results section from this study, re-written by us to render it anonymous. We ask that you focus entirely on replication and assess the verifiability of the study by:

1. replicating their exact models - to the best of your ability
2. checking if your results match the results described in the Results section

The original authors used two waves of *International Social Survey Program* (ISSP) data and a few country-level measures. We link you to these data directly in a shared data folder (they are too large to attach to an email), they are in either *Stata*, .csv, or .xls format and titled ZA2900 (ISSP 1996), ZA4700 (ISSP 2006), and L2data (for the country-level data). Note that in .csv and .xls format the data contain no meta-data (i.e., no variable labels or differentiation between string and numeric) so you might need access to additional documentation. Please work only with the data provided as it is essential to our project that all replication teams work with identical data. If you cannot manage to import or work with one of these formats please contact us for transfering the data into your preferred format.

Shared Data Folder (click to access ISSP and country-level data, if you do not have HTML enabled email please copy and paste the link at the end of this email into your browser).

Please work in the statistical software you normally work with. We ask that you do not learn a new software in order to participate in this initiative. Please be sure that you document all your work and that we can reproduce your results using the code you give us. If you need additional documentation (e.g., codebooks) there are two links at the end of this email, one for each ISSP wave. If during this replication concerns or ideas arise for different or better analytical strategies than employed by the original authors, please keep them in mind for the phase after the replication when you will have the chance to share them and to do them. But for now we ask that you do not yet run additional analyses or alternative model specifications as these might bias your task.

Results should be submitted by September 10th, 2018 to [Nate Breznau]. Please include your code saved in its own language file (e.g., .do, .R, .inp, etc) and a results table in spreadsheet format (.csv, .xlsx, .gsheet etc). We provide an attached Excel “Replication Template Anon” to give you an example of the ideal ‘style’ of results, and if you like you can fill in your results.

We know how much time pressure you may face as a productive scholar, but we must stress the importance of completing the replication on time as the success of the project depends on starting the next phase of the CRI on time. We estimate that this exercise may take between 5 and 14 hours of working time depending very much on your own experience with the data and/or the models employed herein. Thank you for your understanding and participation in this exciting initiative. We remind you that all participants completing the CRI tasks will be co-authors on the final paper where we present the results of the study. Do not hesitate to ask if you have questions or need assistance.

Communication S4b. Replication instructions for Opaque Group, 23/8/2018

Participant teams were randomly assigned to one of two groups. This group was given a summary of the methods and results written by the PIs.

We wanted to inform you that the OSSC19 Crowdsourced Replicated Initiative is on track to be successful, thanks to your participation. We started with 213 registered participants in 103 research teams. 204 participants completed the first survey wave and started replicating the study on immigration and public opinion. The vast majority of research teams submitted their replication results and we will begin analyzing them in the coming weeks.

Now that you completed the first replication phase we hope you will share some details about your experience.

Moreover, we want to ask you about immigration and social policy preferences again. This survey might take 10 minutes. Please note that there is a debriefing about our goals in the first phase of the replication at the end of the survey and a chance for you to offer open-ended feedback if you like.

Please click the following link to begin the survey:

#code_complete#

Note that this is a personalized link. Each participant receives a personalized survey link. Therefore, please do not share the link. If you lose the link or it does not work please contact us. It is possible to interrupt filling out the survey and to continue later if you need to.

We again thank you for your participation in this survey.

Communication S5. Wave 2 survey invitation, 9/12/2018

Replication stage: Thanks for participating!

Thank you for replicating the study by Brady and Finnigan - either directly, or indirectly as an anonymized version! The goal of this first stage was assessing the study's verifiability. We have collected all your results and will analyze them as we discussed in the debriefing at the end of the survey you completed. In case you want to read the debriefing again you will find it here (please make sure you are using HTML enabled email, or see the end of this email for the full link).

In the next phase of the Crowdsourced Replication Initiative, our focus expands beyond the verifiability of Brady & Finnigan's study to the substantive question on the relationship between immigration and policy attitudes and how best to answer this using ISSP data.

Expansion stage: developing a Research Design

The substantive question comes down to the hypothesis that a greater stock or a greater increase in the stock of foreign persons in a given society leads the general public to become less supportive of social policy, where “social policy” refers to any policy that provides basic protections, social insurance, welfare or wellbeing services, income replacement or active labor market programs. In short, what many scholars refer to as the ‘social welfare state’. This was the hypothesis Brady and Finnigan aimed to test, and it is a common hypothesis in the literature.

Your goal is to test this hypothesis in the best way you (and your research team, if applicable) determine. Consider the Brady and Finnigan study as an example of the state of the art. They find no general effect, thus finding proof against the hypothesis. You are now going to consider if this state of the art is adequate, or needs to be re-designed to better test the hypothesis. In doing so you are essentially testing if the finding that there is no effect of foreign-born (in stock or change in stock) on support for social policy is *robust* and *generalizable*.

For this type of crowdsourced research to be successful we need all participants to use the same data source, therefore we ask you to develop your Research Design with ISSP data. However, if you like you are free to incorporate more waves of the ISSP ‘Role of Government’ data. Brady and Finnigan used 1996 and 2006, but there are two earlier waves and one later wave. More information about the samples in these waves is available by clicking on the year (1985, 1990 and 2016) (see end of email for full link text if you do not have HTML enabled). Moreover, this hypothesis focuses on advanced/rich welfare state democracies. Just like Brady and Finnigan we ask that you focus your attention only on these countries. However, we do not define what this means, please use your own understanding of which societies should be included in this list.

We ask that you aim to keep your Research Design to 750 words or less. However, we do not want to constrict your work, so if absolutely necessary you might go *a bit* over. Moreover, you are free, if not encouraged, to use tables and figures (for example, a table of models or variables, path models, equations or DAGs) which you should not count against the 750 words. Please make sure you use all six questions from the ISSP incorporated by Brady and Finnigan. However, we do not want to constrict your creativity or research methods, so if you have a sound argument for adding further questions or alternative forms of measurement (so long as they remain in the Role of Government data), you are free to do so.

We also want to inform you that you will engage in an online deliberation over the Research Designs with the other participants. This is part of the crowdsourcing collaboration method, and it will take place before we present all the results in the final paper. This will be a structured deliberation and it will determine what we will present to the academic community. We therefore hope you put a lot of time and effort into the development of this research design, e.g., modeling and estimation strategies, variables and measurement, and identification. Whatever you deem to be a thorough, or best possible design for testing this hypothesis given these data. It is also important that this Research Design is well developed because you will later execute this design in practice as you have described it - although it is *your research* and if at any point you insist on changing your analyses, we would allow you to do so. If you are in a team and you cannot reach a consensus over the best Research Design, please report what points you disagree over and plan to execute these alternative models as sensitivity tests.

Please submit your Research Design to Nate Breznau [email link] by October 23rd, 2018 in whatever form of document you prefer. As a best practice of open science, we ask you to not run any analyses during this research design phase, construction of the Research Design is only about making the best possible test regardless of results.

Communication S6. Research design, 15/9/2018

Dear Researchers,

Thank you for conducting and submitting the centerpiece of the CRI, the expansion analysis. While processing your results, please take this survey on your impressions of the process and outcome of your research. If you have not yet submitted the expansion please do so ASAP and then take this survey afterwards.

We are acutely aware of your time constraints, yet,this survey is crucial for evaluating our crowdsourcing process and the validity of crowdsourcing as a research method.

Consider it a key part of our joint publication on immigration and policy preferences. In the research designs, we noted strong variation in the measurement design for the dependent variable(s). Therefore one section asks you to explain your analytical choices using open ended questions. We will use your responses to this question when compiling the evidence to be reported in the resulting publication.

Roughly, we estimate that this survey will take about 4 minutes to complete plus the time you need to reflect on your measurement choices

Here is the survey link:

#code_complete#

Note that this link is personalized for you. Therefore, please do not share the link. If you lose the link or it does not work, please contact us. It is possible to interrupt filling out the survey and to resume at a later point if you need to.

We again thank you for your participation in this survey.

Communication S7. Survey invitation wave 3, 24/11/2018

The Next Phase: Expansion

It is time for the Expansion, in which you will conduct analyses according to your submitted Research Design. We kindly ask you to follow your Research Design as closely as possible for this phase. Prior to completion will have the possibility to change your submitted results, in case new ideas emerge during your research or the deliberation after this phase: See the timeline at the end of this email.

Following participant requests, we compiled a database of macro indicators from 1980-2017 for all countries surveyed at least once in the ISSP. Despite some requests for additional data, we only compiled indicators of the measures used in Brady and Finnigan's (2014) original study, including their sensitivity analyses. We do not want to bias your work or the collective product thus we ask that you obtain any additional data yourselves. Moreover, please be cautious when using the harmonized data we supply. We provide these data as a service to you after several participants expressed concerns with time pressure. Still, we are interested in your research and the choice of indicators we provide might be different from what you would have done without our help, so the ideal setting is one where you conduct your own work.

Please submit results by November 22nd, 2018. We ask that you provide all results (full regression tables, marginal effects, plotted margins, etc. -- whatever you find the best and most thorough way); however, we ask that you make sure to provide the following two things:

(1) The marginal effect of a 1% higher or lower stock of immigrants, and the marginal effect of a 1 more person per 1,000 (a 1-point increase in net migration) on the dependent variable(s). We ask that you provide 95% confidence intervals for these margins. We realize this may not be possible for all forms of analyses, but please do the best you can to obtain these estimates. Furthermore, we realize that many of you will analyze data from each of the six outcome-relevant survey questions independently. In such cases, we expect 12 marginal predictions.

(2) We ask that you provide a substantive conclusion based on your test of the hypothesis that a greater stock or a greater increase in the stock of foreign persons in a given society leads the general public to become less supportive of social policy, where "social policy" refers to any policy that provides basic protections, social insurance, welfare or wellbeing services, income replacement or active labor market programs. In short, what many scholars refer to as the 'social welfare state'. Your conclusion should be one of the following options: (a) support, (b) lack of support, or (c) not testable. Importantly, please also provide a short argument (e.g., at least a paragraph) for why you found (a), (b) or (c) as your result. Thank you!

We will rely on these two estimates when compiling results for the final paper, therefore we urge you to be as careful as possible when providing yours!

Additional Data and Codebook

All data are in the folder "CRI Shared Data", here: [link]

The macro data are the file "cri_macro", the codebook explaining measurement and sources is "cri_macro_codebook".

The ISSP data are named according to their original files at GESIS.

Some cautions about the data: Not all indicators are available for all countries and years. Please note that if you know a better source of data on GDP, income inequality, or any other measure of interest, please do what you can to come to the best possible substantive conclusions about our focal hypothesis. Please note that a "." exists where data are missing.

Communication S8. Main analysis, 26/10/2018

First some good news: The hard work of data wrangling and crunching is finished!

Now we need to bring it all together to present our results to the world. Based on our collective work thus far, we identify three decisions that have been contentious or unaddressed by many research designs. Therefore, we call on your expert opinion. This will be done through feedback and voting in an online deliberation environment. This task begins now and should be concluded by December 16th, it is one of the two final research design review tasks before the CRI closes with our final survey wave.

Three Key Research Decisions

Before we introduce the online platform, we ask that you take a moment to review the three following contentious decisions. Please note these do not necessarily reflect our positions, they are open arguments for discussion:

1. Case selection.
To test the hypothesis that immigration undermines public support for social policy it is necessary to look only at rich Western democracies. In their original study, Brady and Finnigan (2014) identified these as Australia, Canada, Denmark, Finland, France, Germany, Ireland, Japan, the Netherlands, New Zealand, Norway, Portugal, Spain, Sweden, Switzerland, the United Kingdom and the United States. They analyzed a sub-sample of 13 out of these 17 countries due to data availability. Research designs testing their hypothesis should only include some or all of these seventeen countries. Any additional countries are inappropriate for testing the hypothesis.

2. Clustered Standard Errors.
In their two-way fixed-effects models, Brady and Finnigan (2014) did not use clustered standard errors at the country-level. Therefore, the significance tests of their regression coefficients are based on thousands of individual-level cases when in fact there are only 13 country cases, or 26 country-time-point cases. Therefore, to truly test the hypothesis that immigration undermines public support for social policy it is necessary to cluster the standard errors for all country-level independent variable coefficients. Otherwise, the estimates are untrustworthy.

3. Power.
One of the teams in the CRI did a power analysis of a bivariate regression with 13 cases repeated in two waves. This basic regression offers the greatest possible power that Brady and Finnigan [2014] had in their original two-way fixed-effects models. They concluded that if the true effect of immigration on social policy preferences is less than 0.16 standardized units (i.e., a Cohen's *d* of 0.16 if an "immigration treatment" is assumed to be a standard deviation in immigrant stock or flow and the mean shift in preferences is on a standardized scale), then there is less than an 80% likelihood of recovering this effect (at .05 alpha). If similar power analyses were conducted for each research design, all research designs in the CRI that do not have 80% power should have their results excluded as unfit for testing the hypothesis.

The Method and Results of Deliberative Voting

This takes place on Kialo, an online deliberation environment designed by communication scholars. It is important for the outcome of our entire crowdsourcing effort that each individual participant does voting here, not just one per team. A randomly selected half of the participants have already gained some experience with Kialo during the Expansion Phase. We have listened to your feedback about your experience and have tried to implement your suggestions for making the exchange of arguments as easy as possible. We prepared detailed instructions to help you get started in Kialo: [link removed]

[*continued on next page…*]

Communication S9. Invitation to deliberation and voting. 12/7/2018

For drafting the substantive conclusion of the CRI, it is important to know whether you support or oppose these three theses and why. In addition to improving the overall quality of our collective results, this task will help us defend our project against potential peer-review criticism and to offer a nuanced and well-thought-out conclusion in our article.

Kialo Login and Password

We will all use first names in Kialo to generate a personal touch, but not get distracted by the academic status that be might be associated with last names. Your first names will have "_cri" at the end, (e.g., Max_cri). We have already set up a Kialo account for you: please log in at www.kialo.com, with the following email address and password (it is not a real email address so please do not try to use it). The reason we set up 'disposable' accounts for each of you is that we do not want to force you to use a third-party website.

[login credentials provided]

!!! *Please do not change your password. We cannot recover your password if you lose it* !!!

We are aware this is a new tool that most of you are not familiar with. We did our best to make it as easy as possible for you to use Kialo. Please view the video we created for you to begin effective use of Kialo.

[link removed]

If you are having trouble we strongly encourage you to contact Nate.Breznau@[redacted]. Nate will be available to answer your questions. You can also address him directly in Kialo using @Nate_Breznau.

Thank you for your participation!

Please provide arguments for (Pro +) and against (Con -) each of the three theses. Please rate the 'veracity' of each design by voting from 0 (least truthful) to 4 (highest level of truth) in your opinion. You may change your arguments or votes at any time. Follow the link below to view the instructional video and contact nate.breznau@[redacted] in case you have any issues or questions.

Communication S9 continued. Invitation to deliberation and voting. 12/7/2018

Dear Participants,

This survey is your final task in the CRI. Please note that this survey may take longer than previous surveys because it has a different purpose than the preceding surveys. The main goal of this final CRI phase is to get your assessment of research conducted by the other teams. We will use this information to generate our collective results to answer the substantive research question about immigration and social policy preferences.

Specifically, you will be given 3 research designs to read; each described in 150 words or less. We want to know your opinion of each design as this will be a key variable in our meta-analysis.

Here is your link to the survey.

#code_complete#

Note that this is a personalized link. Each participant receives a personalized survey link. Therefore, please do not share the link. If you lose the link or it does not work please contact us. It is possible to interrupt filling out the survey and to continue later if you need to.

We again thank you for your participation in this survey and the CRI.

Communication S10. Survey invitation wave 4, 20/1/2019

II. Model Specification Coding and Distribution

The following 10 pages are a table listing the model specifications we identified among the team's software code. It includes a definition of each variable and the percentage of the 1,261 models that include each specification in the column "proportion" and whether that specification was used to investigate variance in results. Those not used were either only present in sensitivity models not in main models (proportion = 0.00%) or present in so few cases that they were not useful.

variable name	definition	proportion	missing	used in analysis
u_teamid	Random team number assignment except team 0, which refers to the Brady and Finnigan study. These specifications are excluded from the analysis but left in here for comparison.	100%	0	No
count	A counter to return results to their original order	100%	0	No
id	Team number plus model number counted in order within teams	100%	0	No
AME	Average marginal effect as produced by team's provided code; or added by PIs to produce when not present.	100%	8	Yes
lower	Lower confidence boundary at 95%CI	100%	9	Yes
upper	Upper confidence boundary at 95%CI	100%	9	Yes
error	The absolute deviation of the high 95% CI from the margin	100%	9	No
z	Z-statistic or equivalent (T-value)	100%	9	No
p	p-value or equivalent confidence interval relative to zero (e.g. for Bayes estimation)	100%	9	Yes
DV	Dependent variable used, single questions are labeled "Jobs" etc., and any scale variables start with "Scale_" followed by the number of items (in the case of 4 it is jobs, unemp, incdif, oldage and 6 is all six; the one case of 5 excludes jobs); "Scale_Deserv/Deserv" means undersving and deservingness criteria were applied to the items	100%	0	Yes
main_IV_type	Test variable type for hypothesis that immigration undermines social policy support. It is either "stock" (% foreign-born), "flow" (change in %, either net migration or a change in "stock"), or "change in flow" reflecting a derivative of flow.	100%	0	Yes
main_IV_source	The data source; note that many teams imputed some countries using other data sources, we have not coded this and left this as the primary source code only. This variable is depricated.	100%	0	No
main_IV_source_file	The name of the source file used.	100%	0	No
main_IV_measurement	Measuring what type of immigrants. "Emigration" is coded as "Immigrant, foreign-born".	100%	0	Yes

variable name	definition	proportion	missing	used in analysis
main_IV_time	The time period that the team used to measure 'flow' of immigrants, 1-year 5-year, etc. Note that the PIs rescaled everything to the equivalent of a 1-year change for comparability, but this refers to the metric of the variable prior to any linear rescaling. Note that some measures are an average of 1year net migration rates over 5-years. For these we code them as 1-year change (even if this is an average). For example, team 7.	100%	0	Yes
main_IV_effect	Total, within or between effect. For non-multilevel models, it is always total. A within effect of stock is "Flow per wave". Note that PIs rescaled all effects to be 1-year equivalent as much as possible.	100%	0	Yes
package	Software package, character categories.	100%	0	Yes
inv_weight	The number of models per team, must be divided by 1 to use for weighting.	100%	0	Yes
num_countries	Number of countries in the model sample	100%	0	Yes
Jobs	Single question on government provision of jobs is the dependent variable, or if 'scale' = 1 it was used as part of the scale	29.18%	0	Yes
Unemp	Single question on government provision of unemployment protection is dependent variable, or if 'scale' = 1 it was used as part of the scale	29.49%	0	Yes
IncDiff	Single question on government reduction of income differences is dependent variable, or if 'scale' = 1 it was used as part of the scale	29.49%	0	Yes
OldAge	Single question on government provision of old-age care is dependent variable, or if 'scale' = 1 it was used as part of the scale	29.49%	0	Yes
House	Single question on government provision of housing is dependent variable, or if 'scale' = 1 it was used as part of the scale	20.40%	0	Yes
Health	Single question on government provision of health care is dependent variable, or if 'scale' = 1 it was used as part of the scale	20.70%	0	Yes
Scale	A multi-item scale was createdd and used as the dependent variable, the questions used to construct the scale are indicated by the previous 6 variables	14.90%	0	Yes
Stock	dichotomous for main_IV_type	49.58%	0	Yes
Flow	dichotomous for main_IV_type	47.14%	0	Yes
ChangeFlow	dichotomous for main_IV_type	3.28%	0	Yes

variable name	definition	proportion	missing	used in analysis
main_IV_as_control	If the other main IV was used in the same model, 0=no, 1=yes (assumes there is only one other main_IV used by the team, although this was not always the case); for example, if both stock and flow are in the same model, this = 1. It is implied that within and between effects of one variance partitioned variable are together in one model (by definition) so these models=0. within/between models only =1 if both stock and 'flow' are used as separate variables (e.g. team 34's second half of models)	32.85%	0	Yes
twowayfe	Two-way fixed-effects (2WFE). Contains dummy variables for country and year, regardless of estimation strategy. The PIs do not prefer the term "Two-way fixed-effects" because it can lead to confusion, but it was used in the Brady and Finnigan study so we follow this nomenclature.	47.36%	0	Yes
cluster_any	Any kind of clustering command added by the researcher. Does not include multilevel model's implicit 'clustering' for each level's equation. Unspecified which level of clustering, or if any of the above three are specified, thus it measures any clustering.	21.47%	0	Yes
mlm_any	Any multilevle model. It = 1 if mlm_re, mlm_fe, and/or mlm_hybrid = 1	52.25%	0	Yes
mlm_re	"Random-effects" model. Any form of multilevel model that estimates separate equations for variance at the individual-level and at a higher level (countries or country-time points). Includes random-intercepts and fixed-coefficients (what is known as a "random-effects model" in econometrics). Thus adjusts for the different variance and different number of cases within and between higher levels. For a single model with both (mlm_hybrid), this is also ticked.	45.00%	0	Yes

variable name	definition	proportion	missing	used in analysis
mlm_fe	"Fixed-effects" model. Any form of multilevel model that estimates separate equations for variance at individual- and higher levels (countries or country-time points). Includes random-intercepts so that country-level variables are mean-centered within countries over time and the model only explains changes in the dependent variable that take place within countries, not between countries (known as "fixed-effects model" in most literature). For a single model with both re and fe (mlm_hybrid), this is ticked along with mlm_hybrid, but mlm_re is not ticked when this case references a model's within-effect (see explanation in "mlm_re". This is also =1 if a country-level first-differences model was used.	7.26%	0	Yes
hybrid_mlm	Includes both re and fe	5.35%	0	Yes
level_cyear	unspecified modeling of country-year level, can include random-effects or dummy variables in a multilevel model	15.58%	0	Yes
level_country	unspecified modeling of country level, can include random-effects or dummy variables in a multilevel model	44.31%	0	Yes
level_year	unspecified modeling of year level, can include random-effects or dummy variables in a multilevel model. Refers technically to "wave" of the survey as years vary slightly within waves.	17.19%	0	Yes
country_dummies_only	If not a 2WFE: Includes a dummy variable for each country. Also includes dummies within a multilevel model but not random-effects intercepts.	0.92%	0	Yes
year_dummies_only	If not a 2WFE: Includes a dummy variable for each year, only. Also includes dummies within a multilevel model but not random-effects intercepts.	17.95%	0	Yes
year_as_count	Year added as a continuous variable. Coded 1 if year is entered as a continuous independent variable and more than two years (waves) are included in the model.	1.68%	0	Yes
logit	Mutually exclusive functional forms: Logistic. Fits "S" shaped logistic curve to explain zeros and ones in the dependent variable. Includes multilevel logistic.	51.80%	0	Yes

variable name	definition	proportion	missing	used in analysis
ologit	Mutually exclusive functional forms: Ordered logistic or probabalistic estimator (probit). Fits logistic curves into the dependent variables' values with the usage of cut-points to allow for several categorical outcomes, i.e., explains the likelihood of being in any one of the several outcomes over another. This includes item-response models, ordered logistic and probit models.	6.88%	0	Yes
lpm	Linear probability model estimation. DV coded 0/1 but linear model used.	5.04%	0	Yes
listwise	Listwise deletion. Cases are dropped from the data if any relevant variable has a missing value for a given observation	96.49%	0	Yes
multimpute	Pairwise information or imputation employed. Accounts for missing data by using either a pairwise estimation (e.g., full-information maximum likelihood) or multiple imputation.	1.22%	0	Yes
ols	Mutually exclusive functional forms: Ordinary least squares estimator	9.01%	0	Yes
mlogit	Mutually exclusive functional forms: Multinomial logistic estimator. Includes multilevel ordered logit or probits	0.92%	0	Yes
ml_glm	Mutually exclusive functional forms: Maximum likelihood. ML or any other iterative version not OLS, Bayes or Logit, includes GLM and MWFE for example.	31.32%	0	Yes
bayes	Mutually exclusive functional forms: Bayesian estimator. Estimated with a Bayesian technique, such as MCMC, to fit posterior probabilities based on prior distributions to arrive at more 'consistent' estimates at level-2.	2.52%	0	Yes
L2boots	Robust standard error, or Bootstrapped level-2 analysis. Employs some form of testing for heteroskedasticity or outliers by removing one country-time/country case at a time and re-running the model, jackknife, sandwich robust, or a "fe robust" in xtreg in Stata.	1.68%	0	No
weights	Any survey weights applied	3.90%	0	No
pseudo_pnl	Constructed a pseudo-panel of individual-level groups	0.61%	0	No
stata	Stata software employed, dummy for package	71.96%	0	Yes
r	R software employed, dummy for package	19.33%	0	Yes
mplus	Mplus software employed, package dummy	5.65%	0	Yes

variable name	definition	proportion	missing	used in analysis
spss	SPSS software employed, dummy	1.53%	0	Yes
mlwin	MLwiN software, dummy for package	2.44%	0	Yes
dichotomize	Dependent variable is dichotomized	61.12%	0	Yes
categorical	Dependent variable has >2 categories	7.33%	0	Yes
mmodel	Measurement model. Uses scaling, factor analysis or item-response to test/generate latent dependent variable/scale. Always with linear estimator.	17.42%	0	Yes
unbalpanel	Unbalanced 'time-series'.Includes different numbers of countries per wave	54.47%	0	Yes
w1985	Includes data from ISSP 1985 wave	3.67%	0	Yes
w1990	Includes data from ISSP 1990 wave	5.65%	0	Yes
w1996	Includes data from ISSP 1996 wave	77.39%	0	Yes
w2006	Includes data from ISSP 2006 wave	94.81%	0	Yes
w2016	Includes data from ISSP 2016 wave	61.19%	0	Yes
australia	country included in sample	95.42%	0	Yes
austria	country included in sample	0.69%	0	Yes
belgium	country included in sample	15.58%	0	Yes
bulgaria	country included in sample	1.83%	0	Yes
canada	country included in sample	77.46%	0	Yes
chile	country included in sample	12.07%	0	Yes
croatia	country included in sample	12.61%	0	Yes
cyprus	country included in sample	1.83%	0	Yes
czechia	country included in sample	35.91%	0	Yes
denmark	country included in sample	46.37%	0	Yes
finland	country included in sample	45.23%	0	Yes
france	country included in sample	96.33%	0	Yes
germany	country included in sample	96.72%	0	Yes
germany_west	distinguished (!not coded, not enough cases)	NA	0	No
germany_east	distinguished (!not coded, not enough cases)	NA	0	No
hungary	country included in sample	37.89%	0	Yes
iceland	country included in sample	14.97%	0	Yes
india	country included in sample	0.46%	0	Yes
ireland	country included in sample	74.26%	0	Yes
israel	country included in sample	22.46%	0	Yes
italy	country included in sample	7.94%	0	Yes
japan	country included in sample	89.69%	0	Yes
korea	country included in sample	27.12%	0	Yes
latvia	country included in sample	37.43%	0	Yes
lithuania	country included in sample	0.99%	0	Yes
netherlands	country included in sample	24.52%	0	Yes
new_zealand	country included in sample	93.74%	0	Yes
norway	country included in sample	97.56%	0	Yes

variable name	definition	proportion	missing	used in analysis
philippines	country included in sample	0.00%	0	No
poland	country included in sample	29.03%	0	Yes
portugal	country included in sample	24.60%	0	Yes
russia	country included in sample	11.61%	0	Yes
slovakia	country included in sample	14.82%	0	Yes
slovenia	country included in sample	37.36%	0	Yes
spain	country included in sample	98.47%	0	Yes
sweden	country included in sample	98.47%	0	Yes
switzerland	country included in sample	95.42%	0	Yes
great_britain	country included in sample	95.11%	0	Yes
n_ireland	distinguished (!not coded, not enough cases)	NA	0	No
usa	country included in sample	96.18%	0	Yes
south_africa	country included in sample	6.42%	0	Yes
taiwan	country included in sample	0.92%	0	Yes
turkey	country included in sample	0.92%	0	Yes
uruguay	country included in sample	0.00%	0	Yes
venezuela	country included in sample	0.00%	0	Yes
orig13	Identical to the original 13 countries used in Brady & Finnigan's 'two-way fixed-effects' models. Equivalent to the "13 richest democracies" in some descriptions.	38.50%	0	Yes
orig17	Identical to 17 countries used by Brady & Finnigan in mlm random-effects models.	2.60%	0	Yes
eeurope	Includes at least 3 Eastern Europe countries	37.66%	0	Yes
allavailable	>21 countries, all available or mostly all	32.62%	0	Yes
emigration_ivC	gross or net out migration, 'flow'	0.46%	0	No
emplrate_ivC	Employment rate (usually of those in the labor force)	37.36%	0	Yes
socx_ivC	Social Expenditures % of GDP, "SOCX"	40.03%	0	Yes
unemprate_ivC	Unemployment rate of those in the labor force (usually means registered unemployed)	12.07%	0	Yes
gdp_ivC	GDP per capita	14.97%	0	Yes
unchange_ivC	annual change in unemp rate	0.00%	0	No
poverty_ivC	50% median (e.g.)	0.00%	0	No
fbunemprate_ivC	foreign-born unemployment rate	0.46%	0	No
fbunempchange_ivC	change in fb unemployment rate	0.46%	0	No
fbeducrate_ivC	fb education rate	0.00%	0	No
fbeducratechange_ivC	change in fb education rate	0.00%	0	No
socxchg_ivC	Change in SOCX	0.00%	0	No
gdpchange_ivC	Any change measure of GDP (1yr/5yr, etc)	0.00%	0	No
ginin_ivC	Gini, not enough cases of pre-tax Gini to differentiate pre/post Also includes one case of top income concentration (WID)	7.49%	0	No

variable name	definition	proportion	missing	used in analysis
mcp_ivC	Multiculturalism Policy Index (https://www.queensu.ca/mcp/home) or MIPEX data used, or IMPIC 'Immigration Policies in Comparative Perspective'	8.56%	0	No
regime_ivC	some form of categorical welfare state or institutional regime type, not including a post-communist split	0.69%	0	No
socialistdummy_ivC	former state socialist societies = 1, everyone else = 0	2.75%	0	No
targeting_ivC	Benefits target groups (v. universal)	0.00%	0	No
socx_programspecific_ivC	Social spending decomposed into single program domains	0.00%	0	No
conservatism_ivC	Conservative (left v right) government political ideology index (e.g., Schmidt index); includes vote-share measures (see Team 82 & 94)	3.82%	0	No
decomm_ivC	some measure of replacement rates (Scruggs/CWED)	0.76%	0	No
tradeunion10_ivC	10 year change in trade union share of employed	0.00%	0	No
subFB_ivC	Subjective foreign-born, country mean	0.00%	0	No
socult_ivC	Socio-cultural proximity scale using country of origin for immigrants (see Team 29)	0.61%	0	No
fract_ivC	Ethnic fractionalization/Herfindahl index - e.g., from UN stock-by-origin data, e.g., Alesina. Teams that used more than one fractionalization variable (only Team 62) are coded simply as "1" here.	6.72%	0	Yes
antiimm_ivC	Aggregate measures of anti-immigrant attitudes/sentiment from other surverys (e.g., ISSP National identity or ESS).	0.92%	0	No
pop_ivC	Population of country	0.46%	0	No
age_iv	Age as continuous	89.92%	0	No
age2_iv	age-squared, or a categorical break down (i.e. a non-linear age function)	82.58%	0	No
sex_iv		93.12%	0	No
employed_iv	Understood as employed or as categorical variable with self, public, full, part, etc.	92.82%	0	No
income_iv		56.15%	0	No
occclass_iv	Occupational Class	1.22%	0	No
occstatus_iv	Occupational Status	0.23%	0	No
education_iv	Very rough. Any measure of attainment or years, in any number of categories. Future research may want to consider a finer grained coding here.	91.14%	0	No
married_iv	marital status	18.87%	0	No

variable name	definition	proportion	missing	used in analysis
household_iv	household composition (unspecified)	19.71%	0	No
reldenom_iv	religious denomination	1.22%	0	No
relattend_iv	religious service attendance	14.51%	0	No
publice_iv	employed in the public sector	4.81%	0	No
urban_iv	urban/rural/suburban, unspecified	14.36%	0	No
fb_iv	foreign-born, respondent in the ISSP	0.00%	0	No
cuts_iv	subjective attitude government make cuts	0.23%	0	No
taxes_iv	subjective attitude government tax more/less	0.00%	0	No
upol_iv	subjective attitude interseted in politics	3.90%	0	No
leftright_iv	left-right subjective political ideology, or actual reported party vote coded into left-right categories	5.19%	0	No
trust_iv	political trust	1.07%	0	No
efficacy_iv	efficacy (believes he/she can influence government)	0.92%	0	No
fbXnet	interaction indicated by "X"	1.99%	0	No
netXcons	net migration*conservatism index	0.00%	0	No
netXeduc	interaction indicated by "X"	0.23%	0	No
netXage	interaction indicated by "X"	0.00%	0	No
netXsex	interaction indicated by "X"	0.00%	0	No
netXunemp	interaction indicated by "X"	0.15%	0	No
netXinc	interaction indicated by "X"	0.92%	0	No
fbXeduc	interaction indicated by "X"	0.23%	0	No
fbXage	interaction indicated by "X"	0.00%	0	No
fbXsex	interaction indicated by "X"	0.00%	0	No
fbXunemp	interaction indicated by "X"	1.07%	0	No
fbXgini	interaction indicated by "X"	0.00%	0	No
fbXurban	interaction indicated by "X"	0.00%	0	No
fbXinc	interaction indicated by "X"	0.00%	0	No
squared_imm	making a quadratic form for one or both immigration variables	2.44%	0	No
fractXfb	interaction indicated by "X"	0.00%	0	No
fbXleftright	interaction indicated by "X"	1.22%	0	No
anynonlin	Used any nonlinearity, =1 if any above =1 in a few cases if interaction variable not in list (e.g., team 98 immigration*party voting); includes one case of a squared-DV (team 29)	7.26%	0	Yes
Hsupport	What the researchers themselves conclude, presumably includes support of all (a majority) of test variables related to immigration but not a guarantee - team prerogative. Immigration undermines social policy preferences, and the evidence from the team supports this enough to claim it.	10.70%	0	Yes

variable name	definition	proportion	missing	used in analysis
Hreject	What the researchers themselves conclude, presumably includes support of all (a majority) of test variables realted to immigration but not a guarantee - team perogative. Inconclusive evidence in support of the hypothesis is also considered a "rejection" here. We are specifically testing that it does reduce social policy preferences, so if it does not clearly reduce them but the test is reasonably reliable or trusted, it is rejected.	58.52%	0	Yes
Hnotest	What the researchers themselves conclude, presumably includes support of all (a majority) of test variables realted to immigration but not a guarantee - team perogative.. This hypothesis is not testable, or evidence inconclusive to support or reject, with these data.	8.94%	0	Yes
Hmixed	This could include 'mixed' results where some preferences go up and others go down as a function of stock or flow of immigrants, but 'Hmixed' is only recorded if the researcher draws two separate conclusions about stock and flow and that these conclusions are each internally consistent enough (subjective, no systematic code) to warrant conclusive arguments. The teams should check that these are accurate codes.	21.85%	0	Yes
Hsupport_stock	We only list specific in case the researchers claim "mixed" support or specifically report hypothesis tests separately by type of test variable in addition to offering an overal conclusion. Otherwise missing.	16.20%	943	Yes
Hreject_stock	see above	11.76%	943	Yes
Hnotest_stock	see above	0.00%	943	No
Hsupport_net	see above	10.39%	943	Yes
Hreject_net	see above	16.20%	943	Yes
Hnotest_net	see above	1.38%	943	Yes

Table S12. Model specification and coding

Qualitative Coding Notes

General Notes:

When authors did not provide a Stata version we set it at version 15.

Latent variables do not have natural metric. When they are standardized this blurs between and within effects. We decided that for parsimony the best option is to take the standard deviation of the variable with the largest variance among the six for the original 13 countries. The result is 0.48 (cropped) as the standard deviation for the variable "Jobs". We re-scale all latent variables to have this standard deviation.

For models using the data we provided: We divide the net migration variable by 10 to make them equivalent to the percent foreign-born scale. For example, a net migration of 20 is equal to 20 more people out of 1,000 that are foreign-born. That is equivalent to a 2% increase in foreign-born. Dividing the variable by 10 is equivalent to multiplying the coefficient by 10

For models using the bradyfinnigan2014.dta file: We divide the netmigpct variable by 5 because it is in actual percentage values, but is measured over a 5-year period.

Margins calculated from variables measured over more than one year are multiplied by the number of years. For example, the effect of a 1 point change in net migration over 5 years (a .2 point change per year on average), would be 5 times larger if that 1-point change occurred over just 1-year (i.e., .2*5). When it is "per wave" we take an average of the gaps by wave. This means that a 1% change increase in immigrants in the period of one year is extremely unlikely, maybe never happened in this time period (Spain exception?). Therefore, consider taking the average change across Europe per year and multiplying the coefficients.

In cases of MLwiN or certain models, margins cannot be calculated. The hand calculation is coefficient + or - 1.96*SE or a 'hack' odds-ratio calculation, see Team 30.

For t-scores and p-values https://www.bmj.com/content/343/bmj.d2304

Team-specific Notes:

Team 0:

Note used in our main analyses. These are the hand-coded results from Brady & Finnigan's 'state of the art' study.

netmigpct=netmigpct/5

Team 1:

These were their 'ideal' models. If PIs understand correctly, the (emprate + foreignpct + socx + netmigpct):year:country command is trying to correct the country-year measures of these level-2 variables. We are worried that this is trying to estimate a within-effect for these variables and this is the source of the problem. There are not enough years to get a within country over-time effect. But we are not experts with brms models, so we are unsure. We are torn between asking the team to change their models (the reality of research where preferred/ideal models are not always possible)

and keeping the models as they are (the reality of results, that sometimes they just don't converge or work out). However, given our goals of understanding model specifications and subjective conclusions by the teams, we would hope that the team considers updating the models so that they converge. For example, changing to gaussian is one idea, maybe separating the level-2 variables into within and between variance by hand rather than asking the program to do it.

Team 2:

Netmig*10

5-year model AME*5

By making Figure 4 they indicated their preferred models

Team 3:

netmigpctLag/10

Team 5:

Refugee 3-year flow AME*3

Ask about source of refugee data

Note that refugee stock and flow models also include immigration

Team 6:

AME for flow is AME*10

Team 7:

Divided netmig variable by 10

Re-scaled the latent variable

Their measure is average yearly net migration over a 5-year period, so it is actually “Immigration Flow, 1-year” no need to re-scale

Team 8:

Latent*0.48

Their code drops all data from 2016, so that reported results are only for 1996 & 2006

Team 10:

AME*10

Missings were still present for gr_health and gr_house, PI recoded:

*PI adjustment

recode gr_health gr_house (8 9=.)

Team 12:

netmig/10

Their coding drops 1985, 1990 and 2016 even though they merge these data in. The problem is the country-level values of migration as far as I can tell.

Team 13

For some reason Spain is dropped for 1996, thus we code ‘unbalanced panel’

Team 16:

netmig/10

Had to make some rearrangements in R to get the code to produce the data file for Mplus, seems like some things were added after the code was run but then not re-run. Eventually we got an exact replication in Mplus 8; however, the DVs were not reverse coded to make support = to higher values. Rather than reverse the signs of the effects by hand we recoded the 4 items prior to the analysis. This led to nearly identical results as reversing the signs, but not 100% identical. Maybe the Bayesian routine led to some rounding error.

Team 17:

netmig/10 prior to analysis

Latent rescaled to 0.48 sd

7.75 years per wave average*within effect

Team 18:

netmigpct/10

Slightly different margins (within 0.01) due to using the margins command. They have done their own margins calculation at means.

Nice figure for leave-one-out analysis, consider adding to appendix

Team 19:

netmig/10

Latent rescaling not necessary as they used rowmean

Team 20:

mignet_un/10

Had to change the SD of their latent variable to 0.48

3 countries are dropped in their linear model, the model is not “rank”, i.e., there are too many variables and some cells are not identified. What to do?

Margins command cannot estimate lower and upper bounds. Calculate them by hand using the formula

Tomasz sent an email 07/15/2019 explaining why marginal effects and coefficients are equal in linear models, maybe consider referring to this for writing the methods section (see Expansion Phase folder)

Team 21:

Netmig/10

Fscore*0.48

Used within and between measures of control variables, only team that did this I think, so not worth coding as a model spec.

Team 22:

Have not reproduced the variable borrowed from the ESS, just using the team’s provided file

Team 23:

netmigpct/5

Team 26:

wfs_cfa*1.707 to have s.d. of 0.48

mignet_un/10

Change in flow left in original metric (rather than multiplied by 7.75), we don’t really have a metric for this, or argument for changing it

Team 27:

ntmigpct/10

The coding of yr2006 is only done for 1996 and 2006, this means that in the regressions, 2016 gets dropped, so the estimation sample only includes 1996 and 2006.

Team 28:

mignet_un/10

Preferred models are 1-3 (not 4 and 5), as reported in the excel file.

Team 29:

“delta_stock” is their label for net migration, therefore delta_stock/10

We use Model 1 for the preferred models. Although they have an interactions in their models 2 (fb*unemp) and 3 (fractionalization), none of these models show any effects of the interactions, so we stick with model one.

The margins are calculated as AMEs. Non-linear effects hack: effect and effect squared are simply added together. This gives a kind of effect at the mean. It is misleading in a way, but it is the best I can think of to make it comparable to other teams’ results.

This means that every other group of four models has the DV-squared added in (1-4 normal, 5-8 are 1-4 with the addition of the DV-squared and so forth).

Team 30:

For now we multiplied their netmig effect by 10

We selected the models without the fractionalization interactions because they show almost no difference to the other models and they are easier to calculate margins

They use Stata to call MLwin, as we do not have MLwin we use a work around to estimate marginal effects based on their logit coefficients. calculate margins based on odds ratios under the assumption that individuals were 0.5 on an agree(=1)/disagree(=0) scale on the DV, thus the OR is a change in their position from 0.5 (formula is (OR*0.5)-0.5 for marginal change), and that a significant odds-ratio means that the change in odds does not cross zero (very rough). WE thus calculate the confidence interval (CI) with the percentage difference in the z value from 1.96 (as the critical ‘t’) and the CI = margin + (z/1.96).

Team 31:

netmigpct/10 (“netmigpct” is net migration)

cri_macro2.xlsx is identical to cri_macro.xlsx, just formatting and missings have been adjusted

Team 32:

[latent] welfarestate*0.48

Immigration_Specific.xlsx is identical to cri_macro for indicator variables, and is from UN migration statistics by country for main_IV

Team 33:

mignet_un/10

Team 34:

Staterest is rowmean, so no s.d. Adjustment needed

cy_diff_stock/7.75 to account for the survey wave average length

netmig/10

Cross-classified model

Subjective class, only team that uses it

Team 35:

net mig by 10 on provided results

Reversed the signs on the effects as they did not do this

Results differ between Mplus version 8.4 and 7.4, the team preferred to go with 7.4

Team 36:

Has country specific effects for some individual-level controls. Only team, no need for a code.

Team 37:

Latent is by rowmean, s.d. Adjustment not necessary. PI ran models on welfare not welfare 10 , which was produced by "generate welfare10 = welfare*2.5"

Within effect/7.75

"Macro data3 wide.dta" is just a wide format of cri_macro.dta

"Wave" is in as a counter, but not a dummy

Uses ZA4748.dta for an income recode

Team 38:

Latent rowmean, no need to adjust s.d.

mignet/10

Team 39:

We ran all the AMEs for all possible models for the linear + stock option (72/4 = 18 AMEs), couldn't get the AMEs reported in the PDF file. The same applies for the linear + change

Note: Stata's reghdfe command is identical to a country and year ('two-way fixed effects') regression (Nate tested this), so long as the other components of the model are identical

Team 40:

Latent sumscale, no need for s.d. Adjustment

Net migration/10

They didn't use any L1 IVs, all coded 0

Added models 14a, 14b and 14c with percent foreign-born as a variable, their correlations implied that they wanted to run these models, but maybe forgot to add them

Team 41:

netmig*10

stock-within*7.75

DVs are not recoded, 1=strongly agree, so we reversed the signs of their marginal effects

Their M1 Mplus code had a mistake, it had type is twolevel and estimator Bayes, but all others had type is threelevel and estimator is MLR, so we changed model 1 to this, they agreed.

Team 42:

The datafile "countrydata_importV2.dta" appears to be a reshaped version of cri_macro.dta

Latent is rowmean, no need to adjust s.d.

netmig/10 (for scaling)

netmig/7.75 & foreignpct/7.75 (for fixed-effect model by wave)

Level is age-gender-cohort by country. Coded as country for now.

Birthyear ('age') and sex are used to identify cohorts (not education), so code 1 for indep variables

Team 43:

"bruttomig.dta" converted to "rawmig.dta" to protect anonymity

"makro_new.dta" converted to "macro_new_team43.dta"

1996 & 2006 data from bradyfinnigan2014data (thus coded as WDI)

No data for 2016, not sure if this is what the team intended.

Indep variables income and age coded 1, because these were used to construct the pseudo-panel strata

Team 45:

Team 46:

– did a kitchen sink approach. Table 1 might be worth putting in an appendix. It shows the number of significant positive and negative effects for each DV out of 13 models. They indicate their preferred models by reporting marginal effects for 3 versions of model 3 and one version of model 4. However, we ignore their analyses of spending preferences for now as they are a different DV.

Removed spending preferences as a DV to clean up code

They imputed UN stock data missing for Germany in 1989 with WB data

Western and non-western immigration measures are a % of total immigration this means they are dependent on the number of immigrants total, but to take this percentage and multiply by number of immigrants would transform the variable. Keep as is for now, but it is not directly comparable as a 1-ppt change in immigration. Estimates are very small (near zero), so don't worry too much about it for the larger picture.

Here 'non-western' is Africa and Middle East/West Asia only.

Team 47:

netmig=netmig/10

Team 48:

Renamed Mplus files and the new_2.csv to 'team48'.

Removed the standardization of the immigration variables to allow for comparability across teams.

Signs are reversed in the excel results for the scale models (team48.1.inp), we fixed this by adding the code from their single-item models (team48.2.inp).

Netmig/10 post-hoc.

Individual DVs are standardized. This is possibly the only team doing this, but it likely has an impact on measurement.

Team 49:

netmigpctN=netmigpctN/10

a power analysis, might make a good appendix.

They did equal weighting. So weighted with ISSP weights and then weighted to have equal cases by country-year. Maybe the only team that did this...

Team 52:

netmig/10

Models 25-36 have both oecd and non-oecd net migration and oecd and non-oecd stock as variables, but only margins for the net migration variables.

macro data comes from three sources, where country.dta contains the variables originally used, cri_macrodata.dta contains variables shared within the Replication Initiative, macrodata.dta contains data specific to our design, drawn from OECD

Note that “main_IV_measurement” is a net migration measure for “Western Immigrant” and “Non-Western Immigrant” but we have not distinguished this, as this is already a rare code. For example introducing a new “Western Immigrant, Net” and “Western Immigrant, Stock” differentiation would simply uniquely identify this team.

Team 54:

has a power analysis, might make a good appendix. Although they provide estimates by individual DV, they argue for a 4 item scale so we only use their latent variable models.

netmig/10

There is a problem with the weighting variable, in several countries the mean of variable ‘weight’ is in the ten thousands. It should be around one.

Team 56:

netmigpct/10

Converts data to a long format that includes each response (out of 4) per DV separately. In a way this is like a multinomial logit. Results are then the average effect of immigration on the likelihood of the individual giving a positive response across all six items.

dichotomize=1, it is dichotomized but the result is then the average effect across all item, response possibilities per person.

Team 58:

mignet/10 & change10/10

/* This team argued for effects being different by response. Therefore,

we allow their margins to be categorical as well. We consider the

cutpoints in recreating a single linear effect

Treat four ordered logits as 1, 2, 3 and 4 and then estimate the

difference between the population mean with this coding scheme and

the predicted population mean based on the likelihood of being in each

category given a 1 point higher value of the indep. variable. This gives

an approximation of what the overall population mean would be given

unequal effect intervals.

The alternative to this would be to predict(xb) and have a linear effect,

but our coding here allows the sampled values in the data to change

differently for each parameter given a 1 point change in the

independent variable */

Team 59:

mignet_un_ln/10

Ran same 24 models with leaving out certain countries, but claimed that leaving out Japan as a ‘special case’ would require a different hypothesis. Therefore, we conclude that the first 24 models are the preferred models.

PI note: It was necessary to add data = na.omit() to all models to replicate

PI note: 'as.numeric' was added to the socx_oecd variable. This was not present in all models in the team's original code.

PI note, in order to extract margins efficiently we reorganized the model code (and reordered it to match our internal ordering structure)

Stock and netmig data are missing for 2016

Team 60:

Team 61:

Net mig/10

Their code is also in Rcloud.

https://rstudio.cloud/project/445619

We had to make path changes for their code to run. We used our own path function “pfunc” everywhere they had ‘path’ or other calls to files

Note that the countries and years imported do not all remain in the analysis. 1985 wave is dropped for the models using immigrant stock, and the 2016 wave is dropped for the models using net migration.

The team reported overall average marginal effects, but this blurs the different effects for the rich democracies (as analyzed by Brady and Finnigan) and all the rest of the countries. We have reorganized the margins commands to present both effects as both are unique effects of immigration variables on different subsets of countries and derive from the team’s idea that effects should be similar across all countries if the hypothesis were true..

Team 62:

Given that the team stresses Alesina's work we determine that Table 1 are preferred models. However, the team showed interest in using country fixed-effects, country-dummies. There are not enough degrees of freedom for fixed-effects and the three fractionalization variables and to estimate average marginal effects, so we also included Table 4's models as alternative preferred models but without the interactions.

lat1 = deserved, lat2 = universal old/sick (2 dimensional latent). Coding was reverse of others (lower values = higher support), so we reversed the signs to make higher values = higher support.

netmigpct/5

The used fractionalization indices and these had negative effects, this is a major part of their conclusion in support of the hypothesis, like team 101

Team 64:

Team 65:

incrimmig10y/10

For now I have re-written their SPSS code as Stata code and run their models. The SPSS code they provided has bugs, it does not run. I am able to get their reported results within .001 or at least .02 in the worst cases, team accepted this.

Team 68

miginflow_1/10

att_index has natural metric, no rescale

Team 69:

The first set of models uses absolute number of immigrants. It is the UN "immigrant stock" thus it is not an "inflow" as the team claims. It is also used here as an absolute number regardless of population. Ask the team their opinion on this, if they really want immigrant absolute stock as their variable. PIs assume not, and have recoded it to be relative to the total population.

The second set of models uses immigrants as a percentage of the population in 1000s, thus it is inflated and needs to be divided by 10 to arrive at an actual % of the population that are immigrant. But again this variable is not inflow, it is relative stock. The PIs normally did not alter any team's models but there seems to be a mistake here. The team intended to measure "flow" as they reported in their write-up, therefore the PIs replaced the varaibles in these models with netmig_un from the cri_macro datafile (divided by 10 for comprability) as this is an actual "flow" variable.

This changes the results of course substantially, ask if the team is ok with this and if they want to revise their conclusion that they rejected the hypothesis.

Team 70:

– in their write up they highlight the model including all 4 country-level variables at once, therefore we elect these as their preferred models (M25-M28).

We code it as between-effect because there is only one year analyzed

We need to recode netmigp by 5, best to do it in the SPSS syntax

Team 72:

netmigpct/5 (from the bradyfinnigandata)

Preferred models indicated by their reporting of marginal effects for 'Model 1'

Tried to create a balanced panel of countries, but Spain data in 1996 are not in the models

Team 73:

netmig_un_lag1/10

Table 1 indicates preferred models (no country-level controls)

Brant test and country-specific regressions, maybe for an appendix

This has effects being different by categorical response. Therefore,

we allow their margins to be categorical as well. We consider the

cutpoints in recreating a single linear effect. We treat four ordered logits as 1, 2, 3 and 4 and then estimate the difference between the population mean with this coding scheme and the predicted population mean based on the likelihood of being in eachcategory given a 1 point higher value of the indep. variable. This gives an approximation of what the overall population mean would be given unequal effect intervals.

The alternative to this would be to predict(xb) and have a linear effect, but our coding here allows the sampled values in the data to change differently for each parameter given a 1 point change in the independent variable.

Team 75:

Removed DV standardization

foreignpct_w/7.75

Model 14 didn't converge so there are no CIs. Otherwise results follow the original authors perfectly without rescaling.

Team 77:

Netmig and chg10yr were divided by 10 to put them into a 1 out of 100 per year scale.

Team 82:

preferred models M2,4,6 & 8.

They included asylum seekers as their main test variable

Included their use of “foreign%” as a “Stock” effect.

Team 83:

mignet_un/10

Team 84:

netmig/10

Latent variable rescaled to have a 0.48 standard deviation

Used DAGs, maybe add to appendix

Makes clear that ordered logit is not linear and therefore effects of foreign-born are not constant over the categories. We estimate margins only on their ordered logits, not their simulated categorical outcome specific effects.We can propose to calculate an overall effect using the following formula, but this takes away the overall ‘categorical’ nature. The team should decide if they want to use the following strategy or have some other strategy, but for the purposes of the CRI we need a single marginal effect per immigration variable (for better or worse)

Proposal: To allow their margins to be categorical we could consider categories as having continuous values of 1, 2, 3 and 4 and then estimate the difference between the population mean with this coding scheme and the predicted population mean based on the likelihood of being in each category given a 1 point higher value of the indep. variable. This gives an approximation of what the overall population mean would be given unequal effect intervals, but it reverts back to a continuous variable with categorical effects, so perhaps not what the team wants. Probably it is fine to just leave it with the ordered logits to move forward.

Team 86:

Coded 0 for ‘main_IV_as_control’ because they do not use any flow variable, even though they combine different sources of stock.

Team 87:

Netmig effect*10

Team 93:

mignet/10

Added command CINTERVAL to produce the equivalent of marginal-effects.

Rescaled effets as if the DV had 0.48 standard deviation for comparability.

Models with MCP have only 11 countries instead of the 15 in all other models.

Team 94:

Latent rescaled to have a 0.48 standard deviation

Preferred models determined to be m1a through m6a

Net migration from the World Bank are not per 1,000 inhabitants, and dividing by 1,000 does not create this value. Thus, the team seems to have made a mistake here. The World Bank variable has to be divided by the actual population to generate the appropriate values. Also, the World Bank offers 5-year estimates, so they also have to be divided by 5. PIs fixed this.

Team should be aware that by measuring net migration (as a % of the population) all 6 coefficients become significant and negative. Team should please decide if (A) the had intended to measure net migration (and thus accept our recode of the variable), and (B) if they want to change their conclusion about the hypothesis

Team 95:

netmig_un/10

For models 5-8, foreignpct in 1996 used, thus ‘main_IV_as_control’ coded 1 and fbXnet = 1

Note that this team didn’t reverse the values of the DVs -> signs must be reversed.

Non-linear effects, imm variables squared, not comparable, no alternative to make comparable.

Team 96:

netmigpct/10

forborn_diff9606/10 & forborn_diff0616/10

Consider putting Approach 2 (analyses by groups) in an appendix.

Replaced mfx commands by margins,dydx; got slightly different results. But this is not due to the mfx command, there were many places in the code where we had to make adjustments to get it to run, ask the team about this.

The second set of models uses “reg” but are coded as “ml_fe” because everything is differenced prior to running, so the estimator is coded “ols”.

Team 97:

Appendix: has some nice categorical graphs, showing the non-linear nature.

netmigpct2/10 (it is mignet_un)

This has effects being different by categorical response. Therefore,

Categorical effects, unique per DV category. We try to allow their margins to be ‘categorical’ as well. We consider the cutpoints in recreating a single linear effect. We treat four ordered logits as 1, 2, 3 and 4 and then estimate the difference between the population mean with this coding scheme and the predicted population mean based on the likelihood of being in eachcategory given a 1 point higher value of the indep. variable. This gives an approximation of what the overall population mean would be given unequal effect intervals. Not perfect, but all we could think of because we must have a single ‘marginal’ effect for each immigraiton variable given our research design.

Team 98:

shows interaction with party ID, very different by party, good graph, show in appendix.

changed year to factor in all models, it seems that in some it was being treated as a continuous variable, and we were certain this was not intended.

netmigpct/10

Team 101:

When the PIs use the provided combined dta files, we get the exact results reported. However, when replicating everything from scratch (creating the files using their syntaxes), then the results are different. Their previous results were false, they were aware of this and tried to fix it, but they continued to use the variable “percentfb” after submitting their new results and this seems to be the source of the problem. It leaves many missing cases. Therefore PIs used the foreignpct variable from the bradyfinnigan data, and this works for now. Team should indicate if they find our changes appropriate. Also, some models did not converge and we had to impose iteration limits of 100.

The team concluded support for the Hypothesis because the Alesina fractionalization variable was significant in 1996 for (all?) models, and because the MCP index was significant in 2006 this is also evidence. In a way this suggests their test variable is not immigration but ethnic-diversity; the question is if we should then make this their test variable.

Team 104:

netmig/10

We code the effect of country-level immigration variables as “total-effects” rather than “between-effects” because level-2 is country, thus, it is the total effect without decomposing into year (so the within-country variance over time is mixed with the between-country variance to produce the estimate). Team should double check that this is correct as we are not JAGS experts.

Does the JAGS measurement model listwise delete or account for missing data? We have coded “multimpute” = 1 because we suspect that JAGS can handle missing data using maximum likelihood, but the team should tell us if it is actually listwise deletion so that we can change the coding.

Team 106:

Ctry_keep_direct_replication.csv was missing

Note that for oldage and unemp they use ordered logit and for incdiff and jobs they use ols.

Note that they only use a country dummy in their ordered logit models.

API_SM.POP.NETM_DS2_en_csv_v2_10224691.csv

immgrStock_abs_m = migstock_un/100*pop_tot/1000000 it takes the ratio of stock of immigrants and multiplies it by the population divided by 1 million. This is a value that has no logical interpretation as far as we can tell. If the country has a population less than 10 million the number gets very small (.02), but if the country has a population more than 10 million the number can get very large (up to 49). So we only included their main models with % stock as the others are not comparable. The others should go into an appendix.

mignet_un/10

DVs not reverse coded, so we changed this

III. Participant Survey Codebook

This machine generated codebook applies to the CRI Participant Survey Data available via the Harvard Dataverse. https://doi.org/10.7910/DVN/UUP8CX

WAVE 1: August 20th, 2018

Fielded via Unipark, WAVE 1 was sent to all 213 persons who registered to take part in the CRI. Missing values indicated throughout the codebook by "**.a**". Nine of the registrants did not respond at all, therefore the persistent 9 cases of missing in WAVE 1 are those survey non-respondents, meaning the WAVE 1 respondent sample is 204. This drops further in WAVE 2 to 189 after 15 participants dropped out of the project.

[Machine generated variable]

Name: dispcode
Variable label: Survey Completion

Value	Label	Freq.	Percent
12	Invited, not completed	9	4.2%
31	Completed	196	92.0%
32	Completed after break	8	3.8%

[Teams randomly assigned into two groups]

Name: u_expgroup
Variable label: Experimental Grouping Structure – after random assignment

Value	Label	Freq.	Percent
0	Opaque Replication Group	114	53.5%
1	Transparent Replication Group	99	46.5%

In the Crowdsourced Replication Initiative, we want to ask you a few background questions to gain a general overview of the researchers participating. First, in which area of research have you received your highest degree?

Name: backgr_degree
Variable label: Area of Highest Degree

Value	Label	Freq.	Percent
1	Communication	4	1.9%
2	Economics	10	4.7%
3	Sociology	98	46.0%
4	Political Science	53	24.9%
5	Psychology	17	8.0%
6	Other	13	6.1%
7	Methods-Related Degree	9	4.2%
.a		9	4.2%

Please indicate whether you have published research in the following research areas and / or used multilevel regression analysis in your published research

[question battery follows]

On (im)migration

Name:	v_17
Variable label:	On (im)migration

Value	Label	Freq.	Percent
1	No	128	60.1%
2	Yes, once	33	15.5%
3	Yes, more than one publication	42	19.7%
.a		10	4.7%

On statistics/methods

Name:	v_18
Variable label:	On statistics/methods

Value	Label	Freq.	Percent
1	No	119	55.9%
2	Yes, once	39	18.3%
3	Yes, more than one publication	44	20.7%
.a		11	5.2%

On public policy / welfare state

Name:	v_19
Variable label:	On public policy / welfare state

Value	Label	Freq.	Percent
1	No	143	67.1%
2	Yes, once	27	12.7%
3	Yes, more than one publication	31	14.6%
.a		12	5.6%

On social policy preferences / public opinion

Name:	v_20		
Variable label:	On social policy preferences / public opinion		

Value	Label	Freq.	Percent
1	No	125	58.7%
2	Yes, once	26	12.2%
3	Yes, more than one publication	51	23.9%
.a		11	5.2%

Using multilevel regression

Name:	v_21		
Variable label:	Used multilevel regression		

Value	Label	Freq.	Percent
1	No	73	34.3%
2	Yes, once	52	24.4%
3	Yes, more than one publication	78	36.6%
.a		10	4.7%

How many undergraduate- or graduate-level courses in quantitative data analysis/applied statistics have you taught as the primary active instructor (including primary teaching responsibilities for a lab, Übung, or additional component of a course)?

Name:	backgr_exp_teach_stat		
Variable label:	Teaching Statistics		

Value	Label	Freq.	Percent
1	0	59	27.7%
2	1	27	12.7%
3	2	34	16.0%
4	3	18	8.5%
5	4	12	5.6%
6	5	9	4.2%
7	6	6	2.8%
8	7	4	1.9%
9	8	5	2.3%
10	9	1	0.5%
11	10	1	0.5%
12	10+	28	13.1%
.a		9	4.2%

How familiar are you with multilevel modelling?

Name:	backgr_exp_famil_mlm		
Variable label:	Familiarity with multilevel modelling		
Value	Label	Freq.	Percent
1		4	1.9%
2		14	6.6%
3		65	30.5%
4		82	38.5%
5		39	18.3%
.a		9	4.2%

The topic of the OSSC19 Crowdsourced Replication Initiative is immigration and social policy. Specifically, it wants to test a common hypothesis in the literature that a greater stock or a greater increase in the stock of foreign persons in a given society leads the general public to become less supportive of social policy, where "social policy" refers to any policy that provides basic protections, social insurance, welfare or well-being services, income replacement or active labor market programs. In short, what many scholars refer to as the 'social welfare state'. We will replicate a study working with survey data asking questions of the public about the social welfare state, but we are also interested in what you think.

Is it your belief that higher levels of immigrant stock or greater increases in immigrant stock in a given country reduces public support of social welfare policies in general?

Name:	belief_H1_1		
Variable label:	Personal Belief about H1, general		
Value	Label	Freq.	Percent
1	Immigration strongly reduces support of social policies.	6	2.8%
2	Immigration somewhat reduces support of social policies.	114	53.5%
3	Immigration has no effect on support of social policies.	70	32.9%
4	Immigration somewhat increases support of social policies.	14	6.6%
.a		9	4.2%

Now please give us your statement on this topic more specifically. In each of these policy domains, how do you think of higher stocks of immigrants?

If you are unsure, please indicate your best guess.

[question battery]

Old age care?

Name:	belief_agecare_1		
Variable label:	Old age care		

Value	Label	Freq.	Percent
1	Immigration strongly reduces support of social policies.	9	4.2%
2	Immigration somewhat reduces support of social policies.	19	8.9%
3	Immigration has no effect on support of social policies.	161	75.6%
4	Immigration somewhat increases support of social policies.	15	7.0%
5	Immigration strongly increases support of social policies.	0	0.0%
.a		9	4.2%

Unemployment?

Name:	belief_unempl_1		
Variable label:	Unemployment		

Value	Label	Freq.	Percent
1	Immigration strongly reduces support of social policies.	26	12.2%
2	Immigration somewhat reduces support of social policies.	115	54.0%
3	Immigration has no effect on support of social policies.	43	20.2%
4	Immigration somewhat increases support of social policies.	20	9.4%
5	Immigration strongly increases support of social policies.	0	0.0%
.a		9	4.2%

Income redistribution?

Name:	belief_income_1		
Variable label:	Income redistribution		

Value	Label	Freq.	Percent
1	Immigration strongly reduces support of social policies.	24	11.3%
2	Immigration somewhat reduces support of social policies.	100	46.9%
3	Immigration has no effect on support of social policies.	70	32.9%
4	Immigration somewhat increases support of social policies.	10	4.7%
5	Immigration strongly increases support of social policies.	0	0.0%
.a		9	4.2%

Housing?

Name:	belief_housing_1

Variable label:	Housing

Value	Label	Freq.	Percent
1	Immigration strongly reduces support of social policies.	11	5.2%
2	Immigration somewhat reduces support of social policies.	95	44.6%
3	Immigration has no effect on support of social policies.	80	37.6%
4	Immigration somewhat increases support of social policies.	17	8.0%
5	Immigration strongly increases support of social policies.	0	0.0%
.a		10	4.7%

Active labor market programs?

Name:	belief_labour_1
Variable label:	Active labor market programs

Value	Labe	Freq.	Percent
1	Immigration strongly reduces support of social policies.	9	4.2%
2	Immigration somewhat reduces support of social policies.	64	30.0%
3	Immigration has no effect on support of social policies.	85	39.9%
4	Immigration somewhat increases support of social policies.	44	20.7%
5	Immigration strongly increases support of social policies.	2	0.9%
.a		9	4.2%

Health care?

Name:	belief_health_1
Variable label:	Health care

Value	Label	Freq.	Percent
1	Immigration strongly reduces support of social policies.	9	4.2%
2	Immigration somewhat reduces support of social policies.	50	23.5%
3	Immigration has no effect on support of social policies.	135	63.4%
4	Immigration somewhat increases support of social policies.	9	4.2%
5	Immigration strongly increases support of social policies.	1	0.5%
.a		9	4.2%

You just indicated your beliefs about the effects of immigration on public support of social policies. How certain are you that your beliefs about this relationship are correct?

Name: belief_certainty_1
Variable label: Certainty in belief about immigration hypothesis

Value	Label	Freq.	Percent
1	very uncertain	28	13.1%
2		63	29.6%
3		29	13.6%
4		45	21.1%
5		29	13.6%
6		8	3.8%
7	very certain	2	0.9%
.a		9	4.2%

Now, we are interested in your own opinions on the substantive topic of the Crowdsourced Replication Initiative. Do you think that, in your current country of residence, laws on immigration of foreigners should be relaxed or made tougher?

Name: attitude_immigration_1
Variable label: Personal opinion on immigration laws

Value	Label	Freq.	Percent
1	Immigration laws should be relaxed	37	17.4%
2		57	26.8%
3		49	23.0%
4		40	18.8%
5		10	4.7%
6		7	3.3%
7	Immigration laws should be made tougher	2	0.9%
.a		11	5.2%

Some political topics are of greater personal importance than others. How important is the issue of immigration to you personally?

Name: attitude_importance_1
Variable label: Attitude Importance

Value	Label	Freq.	Percent
1	not important at all	4	1.9%
2	not very important	15	7.0%
3	moderately important	53	24.9%
4	important	92	43.2%
5	very important	40	18.8%
.a		9	4.2%

After all members of your team have completed this survey, you will receive data and instructions to replicate a scientific study on the effects of immigration on public opinion (later you will expand this study). The study is titled "Does Immigration Undermine Public Support for Social Policy?" by David Brady and Ryan Finnigan, published 2014 in the American Sociological Review. Do you know this study?

[asked only of Transparent Group]

Name: awareness_study
Variable label: Awareness of Original Study

Value	Label	Freq.	Percent
1	I do not know about this study.	57	26.8%
2	I may have heard of this study.	25	11.7%
3	I know this study but I cannot remember many details.	11	5.2%
4	I know this study and I am aware of its methods and of its findings.	3	1.4%
.a		117	54.9%

[Machine constructed variable from IP address]

Name: participant_continent
Variable label: IP address continent

Value	Label	Freq.	Percent
3	Europe	161	75.6%
4	Asia	4	1.9%
5	North America	31	14.6%
6	Africa	4	1.9%
8	South America	4	1.9%
.a		9	4.2%

WAVE 2: September 12th, 2018

From this wave onwards we exclude the 24 cases of non-start or drop out before completion. The leaves us with a final sample of **189 participants**. In this wave we randomly assigned remaining participants to the Deliberation or Control group.

Name:	u_delibtreatmentgroup		
Variable label:	Grouping Variable, random assignment to interim deliberation		
Value	Label	Freq.	Percent
0	Control Group	96	50.8%
1	Deliberation Group	93	49.2%

Regarding this first phase of the CRI: Regardless of how much your entire team spent on the replication work, how much time did you individually spend on the replication (incl. Preparation, Syntax, Submission of Results, Coordination with Team Members etc.)?

[Open response, in hours]

Name:	v_33		
Variable label:	Perceived individual time spent on replication		
Value	Label	Freq.	Percent
0.0000		1	0.5%
1.0000		2	1.1%
1.5000		2	1.1%
2.0000		5	2.6%
3.0000		9	4.8%
4.0000		16	8.5%
5.0000		17	9.0%
6.0000		22	11.6%
6.5000		1	0.5%
7.0000		20	10.6%
8.0000		23	12.2%
9.0000		4	2.1%
9.5000		1	0.5%
10.0000		25	13.2%
11.0000		3	1.6%
12.0000		4	2.1%
14.0000		2	1.1%
15.0000		7	3.7%
16.0000		4	2.1%
18.0000		1	0.5%
All higher values		17	9.0%

How difficult did you find the replication task in this first phase?

Name: v_34
Variable label: Difficulty of Replication

Value	Label	Freq.	Percent
1	This replication was one of the most difficult research tasks I ever completed.	6	3.2%
2	This replication was difficult.	18	9.5%
3	This replication was neither too easy nor too difficult.	80	42.3%
4	This replication was easy.	76	40.2%
5	This replication was one of the easiest research tasks I ever completed.	2	1.1%
.a		7	3.7%

How familiar are you with the social science literature related to the hypothesis that "a greater stock or a greater increase in the stock of foreign persons in a given society leads the general public to become less supportive of social policy"?

[multiple choice, multiple response battery]

Except for the replication work I just performed, I am not at all familiar with this literature

Name: v_35
Variable label: Not familiar with lit

Value	Label	Freq.	Percent
0	not quoted	96	50.8%
1	quoted	86	45.5%
.a		7	3.7%

I have read some of the works in this literature.

Name: v_36
Variable label: Some

Value	Label	Freq.	Percent
0	not quoted	101	53.4%
1	quoted	81	42.9%
.a		7	3.7%

I have read many of the works in this literature.

Name:	v_37		
Variable label:	Many		

Value	Label	Freq.	Percent
0	not quoted	168	88.9%
1	quoted	14	7.4%
.a		7	3.7%

I have published articles or books in this literature.

Name:	v_38		
Variable label:	Published		

Value	Label	Freq.	Percent
0	not quoted	177	93.7%
1	quoted	5	2.6%
.a		7	3.7%

I have taught courses on this subject.

Name:	v_39		
Variable label:	Taught		

Value	Label	Freq.	Percent
0	not quoted	176	93.1%
1	quoted	6	3.2%
.a		7	3.7%

I often discuss this topic with colleagues informally.

Name:	v_40		
Variable label:	Discuss topic		

Value	Label	Freq.	Percent
0	not quoted	159	84.1%
1	quoted	23	12.2%
.a		7	3.7%

Did you enjoy this first replication task?

Name:	v_41			
Variable label:	Enjoyment of Replication			
Value	Label		Freq.	Percent
1	This replication was extremely fun.		20	10.6%
2	This replication was somewhat enjoyable.		106	56.1%
3	Neutral		41	21.7%
4	This replication was mostly not enjoyable.		9	4.8%
5	This replication was not fun at all.		6	3.2%
.a			7	3.7%

After completing this replication task, how well do you think the original study tested Brady and Finnigan's hypothesis question, 'does immigration undermine public support for social policy' across advanced welfare state democracies?

Name:	v_43			
Variable label:	Belief in Original Study			
Value	Label		Freq.	Percent
1	The study provided a convincing test of this hypothesis		1	0.5%
2	The study provided a decent test of this hypothesis.		57	30.2%
3	The study somewhat tested the hypothesis.		69	36.5%
4	The study provided a weak test of this hypothesis.		38	20.1%
5	The study provided no convincing test of this hypothesis.		14	7.4%
.a			10	5.3%

Finally, we repeat a few questions from the first survey wave. The topic of the OSSC19 Crowdsourced Replication Initiative is immigration and social policy. Specifically, it wants to test a common hypothesis in the literature that a greater stock or a greater increase in the stock of foreign persons in a given society leads the general public to become less supportive of social policy, where "social policy" refers to any policy that provides basic protections, social insurance, welfare or well-being services, income replacement or active labor market programs. In short, what many scholars refer to as the 'social welfare state'.

Is it your belief that higher levels of immigrant stock or greater increases in immigrant stock in a given country reduce public support of social welfare policies in general?

Name: belief_H1_2
Variable label: Personal Belief about H1, general

Value	Label	Freq.	Percent
1	Immigration strongly reduces support of social policies.	2	1.1%
2	Immigration somewhat reduces support of social policies.	99	52.4%
3	Immigration has no effect on support of social policies.	69	36.5%
4	Immigration somewhat increases support of social policies.	10	5.3%
5	Immigration strongly increases support of social policies.	0	0.0%
.a		9	4.8%

Now please give us your statement on this topic more specifically. In each of these policy domains, how do you think of higher stocks of immigrants?

If you are unsure, please indicate your best guess.

[battery]

Old age care?

Name: belief_agecare_2
Variable label: Old age care

Value	Label	Freq.	Percent
1	Immigration strongly reduces support of social policies.	1	0.5%
2	Immigration somewhat reduces support of social policies.	22	11.6%
3	Immigration has no effect on support of social policies.	120	63.5%
4	Immigration somewhat increases support of social policies.	33	17.5%
5	Immigration strongly increases support of social policies.	1	0.5%
.a		12	6.3%

Old age care?

Name: belief_unempl_2
Variable label: Unemployment

Value	Label	Freq.	Percent
1	Immigration strongly reduces support of social policies.	10	5.3%
2	Immigration somewhat reduces support of social policies.	95	50.3%
3	Immigration has no effect on support of social policies.	53	28.0%
4	Immigration somewhat increases support of social policies.	19	10.1%
5	Immigration strongly increases support of social policies.	0	0.0%
.a		12	6.3%

Income redistribution?

Name:	belief_income_2
Variable label:	Income redistribution

Value	Label	Freq.	Percent
1	Immigration strongly reduces support of social policies.	9	4.8%
2	Immigration somewhat reduces support of social policies.	84	44.4%
3	Immigration has no effect on support of social policies.	71	37.6%
4	Immigration somewhat increases support of social policies.	11	5.8%
5	Immigration strongly increases support of social policies.	0	0.0%
.a		14	7.4%

Housing?

Name:	belief_housing_2
Variable label:	Housing

Value	Label	Freq.	Percent
1	Immigration strongly reduces support of social policies.	3	1.6%
2	Immigration somewhat reduces support of social policies.	70	37.0%
3	Immigration has no effect on support of social policies.	81	42.9%
4	Immigration somewhat increases support of social policies.	22	11.6%
5	Immigration strongly increases support of social policies.	0	0.0%
.a		13	6.9%

Active labor market programs?

Name:	belief_labour_2
Variable label:	Active labor market programs

Value	Label	Freq.	Percent
1	Immigration strongly reduces support of social policies.	3	1.6%
2	Immigration somewhat reduces support of social policies.	60	31.7%
3	Immigration has no effect on support of social policies.	79	41.8%
4	Immigration somewhat increases support of social policies.	32	16.9%
5	Immigration strongly increases support of social policies.	3	1.6%
.a		12	6.3%

Health care?

Name:	belief_health_2
Variable label:	Health care

Value	Label	Freq.	Percent
1	Immigration strongly reduces support of social policies.	2	1.1%
2	Immigration somewhat reduces support of social policies.	37	19.6%
3	Immigration has no effect on support of social policies.	123	65.1%
4	Immigration somewhat increases support of social policies.	15	7.9%
5	Immigration strongly increases support of social policies.	0	0.0%
.a		12	6.3%

You just indicated your beliefs about the effects of immigration on public support of social policies. How certain are you that your beliefs about this relationship are correct?

Name:	belief_certainty_2
Variable label:	very uncertain - very certain

Value	Label	Freq.	Percent
1		11	5.8%
2		44	23.3%
3		44	23.3%
4		33	17.5%
5		40	21.2%
6		7	3.7%
.a		10	5.3%

WAVE 3: November 24th, 2018

From variable **v_51** to variable **v_66** there is a skip pattern. These are from questions only given to the Deliberation Group (N=93), teams randomly assigned to participate in a preliminary deliberation over the optimal methods for testing the hypothesis. Therefore, for these questions the Control Group (N=96) are omitted.

Before you conducted your analysis you had the opportunity to use Kialo, an online deliberation platform. Did you use Kialo?

Name: v_51
Variable label: Logging into Kialo (Treatment Reception, Complier Status)

Value	Label	Freq.	Percent
1	No, I never logged into Kialo.	7	7.5%
2	I logged into Kialo 1-2 times.	30	32.3%
3	I logged into Kialo 3-5 times.	27	29.0%
4	I logged into Kialo more than 5 times.	13	14.0%
.a		16	17.2%

While logged into Kialo, did you discuss the research design with other CRI participants on the Kialo platform? Did you post pro and con arguments?

[skip pattern, not asked if v_51 == 1 or v_51 == .a]

Name: v_52
Variable label: Participation in Deliberation

Value	Label	Freq.	Percent
1	I did not post anything in Kialo.	38	40.9%
2	I posted once.	11	11.8%
3	I posted a few times.	16	17.2%
4	I was a regular contributor to the discussions.	5	5.4%
.a		23	24.7%

Did you vote on the main theses (e.g., measurement of the dependent variable in Kialo)?

Name:	v_53		
Variable label:	Participation in Voting		
Value	Label	Freq.	Percent
1	I did not vote.	13	14.0%
2	I voted on a few of the Theses in one of the Kialos (e.g., measurement of the DV).	8	8.6%
3	I voted on a few of the Theses in more than one of the four Kialos.	25	26.9%
4	I voted on all of the Theses in each of the four Kialos, but did not use the “Guided Voting" function in Kialo.	10	10.8%
5	I voted on all of the Theses in each of the four Kialos, with the help of the “Guided Voting" function in Kialo.	14	15.1%
.a		23	24.7%

In order to learn from this experience and to improve crowdsourced research, we would like to understand why you did not use Kialo more often. Please indicate how much these reasons for not engaging more frequently in Kialo apply to you personally.

[battery]

I did not expect Kialo to help me much with my tasks in the CRI.

Name:	v_54		
Variable label:	I did not expect Kialo to help me much with my tasks in the CRI.		
Value	Label	Freq.	Percent
1	Reason does not apply to me apply at all.	32	34.4%
2	Reason applies to me a little.	19	20.4%
3	Reason somewhat applies to me.	9	9.7%
4	Reason applies to me a lot.	5	5.4%
.a		28	30.1%

I had too many other responsibilities at the time.

Name:	v_55		
Variable label:	I had too many other responsibilities at the time.		
Value	Label	Freq.	Percent
1	Reason does not apply to me apply at all.	0	0.0%
2	Reason applies to me a little.	10	10.8%
3	Reason somewhat applies to me.	20	21.5%
4	Reason applies to me a lot.	35	37.6%
.a		28	30.1%

I thought that discussion and debate is not an appropriate element of crowdsourced research.

Name:	v_56		
Variable label:	I thought that discussion and debate is not an appropriate element of crowdsourced research.		

Value	Label	Freq.	Percent
1	Reason does not apply to me apply at all.	50	53.8%
2	Reason applies to me a little.	11	11.8%
3	Reason somewhat applies to me.	2	2.2%
4	Reason applies to me a lot.	2	2.2%
.a		28	30.1%

I just do not enjoy engaging in online discussions and debate.

Name:	v_57		
Variable label:	I just do not enjoy engaging in online discussions and debate		

Value	Label	Freq.	Percent
1	Reason does not apply to me apply at all.	19	20.4%
2	Reason applies to me a little.	23	24.7%
3	Reason somewhat applies to me.	16	17.2%
4	Reason applies to me a lot.	7	7.5%
.a		28	30.1%

I found the Kialo process too burdensome.

Name:	v_58		
Variable label:	I found the Kialo process too burdensome.		

Value	Label	Freq.	Percent
1	Reason does not apply to me apply at all.	16	17.2%
2	Reason applies to me a little.	17	18.3%
3	Reason somewhat applies to me.	24	25.8%
4	Reason applies to me a lot.	8	8.6%
.a		28	30.1%

We would like to know how you perceived your experience with Kialo. On the following dimensions, how would you evaluate the overall process of discussion and debate on the Kialo platform?

[battery self-explanatory questions based on value label anchors]

Name:	v_62		
Variable label:	Helpful		

Value	Label	Freq.	Percent
1	Not helpful for exchanging ideas and knowledge	2	2.2%
2		13	14.0%
3		25	26.9%
4		26	28.0%
5	Very helpful for exchanging ideas and knowledge	5	5.4%
.a		22	23.7%

Name:	v_63		
Variable label:	Implementation		

Value	Label	Freq.	Percent
1	Poorly implemented	3	3.2%
2		10	10.8%
3		24	25.8%
4		31	33.3%
5	Carefully implemented	3	3.2%
.a		22	23.7%

Name:	v_64		
Variable label:	Others' arguments		

Value	Label	Freq.	Percent
1	The arguments of the other participants were useful in forming my own position.	2	2.2%
2		22	23.7%
3		19	20.4%
4		23	24.7%
5	The arguments of the other participants were not useful in forming my own position.	5	5.4%
.a		22	23.7%

Name:	v_65		
Variable label:	Atmosphere		

Value	Label	Freq.	Percent
1	Hostile atmosphere	9	9.7%
2		3	3.2%
3		7	7.5%
4		24	25.8%
5	Respectful atmosphere	28	30.1%
.a		22	23.7%

Name:	v_66		
Variable label:	Changed mind		

Value	Label	Freq.	Percent
1	Did not change my mind on the research	16	17.2%
2		16	17.2%
3		25	26.9%
4		13	14.0%
5	Changed my mind on the research substantially	1	1.1%
.a		22	23.7%

We would like to ask you a few questions on the "expansion phase", i.e., the specification of the research design, the discussions you may have had about it, and the analysis you finally conducted. In your own view, how successful was your team in conducting a proper test of the hypothesis in question?

Name:	delib_success		
Variable label:	very unsuccessful - very successful		

Value	Label	Freq.	Percent
1	very unsuccessful	3	1.6%
2		11	5.8%
3		17	9.0%
4		27	14.3%
5		52	27.5%
6		35	18.5%
7	very successful	6	3.2%
.a		38	20.1%

When you consider the entire process of crafting your initial research design from the first thoughts to the final submission, how often did you make substantial changes and revisions to your analysis plans?

Name:	delib_changemind		
Variable label:	Changing minds		
Value	Label	Freq.	Percent
1	Never	9	4.8%
2	Once or twice	82	43.4%
3	Three to five times	53	28.0%
4	More than five times	8	4.2%
.a		37	19.6%

During the CRI, you designed and conducted analyses involving individuals nested in cross-sectional country data at different time points in order to test a substantive hypothesis. Looking back at your CRI experience thus far, did you learn something new (e.g., about methods or analytical choices you were not aware of previously)?

Name:	delib_learn		
Variable label:	Learning		
Value	Label	Freq.	Percent
1	I did not learn anything	9	4.8%
2		15	7.9%
3		18	9.5%
4		12	6.3%
5		59	31.2%
6		28	14.8%
7	I learned a great deal	11	5.8%
.a		37	19.6%

In hindsight, how would you evaluate the expansion phase in the following dimensions?

[battery self-explanatory questions based on value label anchors]

Name:	delib_gooddifficulty		
Variable label:	Difficulty		
Value	Label	Freq.	Percent
1	Too difficult	1	0.5%
2		14	7.4%
3		28	14.8%
4		71	37.6%
5	Good match with my capabilities	37	19.6%
.a		38	20.1%

Name:	delib_enjoy		
Variable label:	No fun at all - Enjoyable		

Value	Label	Freq.	Percent
1	No fun at all	6	3.2%
2		29	15.3%
3		33	17.5%
4		63	33.3%
5	Enjoyable	20	10.6%
.a		38	20.1%

Name:	delib_notcontrolling		
Variable label:	Controlling instructions		

Value	Label	Freq.	Percent
1	Instructions were too controlling	9	4.8%
2		21	11.1%
3		60	31.7%
4		44	23.3%
5	Instructions were not controlling at all	16	8.5%
.a		39	20.6%

In the last section of the survey, we repeat a few questions from the first survey wave. The topic of the OSSC19 Crowdsourced Replication Initiative is immigration and social policy. Specifically, it wants to test a common hypothesis in the literature that a greater stock or a greater increase in the stock of foreign persons in a given society leads the general public to become less supportive of social policy, where "social policy" refers to any policy that provides basic protections, social insurance, welfare or well-being services, income replacement or active labor market programs. In short, what many scholars refer to as the 'social welfare state'. Is it your belief that higher levels of immigrant stock or greater increases in immigrant stock in a given country reduce public support of social welfare policies in general?

Name:	belief_H1_3		
Variable label:	Personal Belief about H1, general		

Value	Label	Freq.	Percent
1	Immigration strongly reduces support of social policies.	2	1.1%
2	Immigration somewhat reduces support of social policies.	48	25.4%
3	Immigration has no effect on support of social policies.	85	45.0%
4	Immigration somewhat increases support of social policies.	10	5.3%
.a		44	23.3%

Now please give us your statement on this topic more specifically. In each of these policy domains, how do you think of higher stocks of immigrants?

If you are unsure, please indicate your best guess.

[battery]

Old age care?

Name: belief_agecare_3
Variable label: Old age care

Value	Label	Freq.	Percent
1	Immigration strongly reduces support of social policies.	1	0.5%
2	Immigration somewhat reduces support of social policies.	13	6.9%
3	Immigration has no effect on support of social policies.	113	59.8%
4	Immigration somewhat increases support of social policies.	17	9.0%
.a		45	23.8%

Unemployment?

Name: belief_unempl_3
Variable label: Unemployment

Value	Label	Freq.	Percent
1	Immigration strongly reduces support of social policies.	7	3.7%
2	Immigration somewhat reduces support of social policies.	62	32.8%
3	Immigration has no effect on support of social policies.	62	32.8%
4	Immigration somewhat increases support of social policies.	14	7.4%
.a		44	23.3%

Income redistribution?

Name: belief_income_3
Variable label: Income redistribution

Value	Label	Freq.	Percent
1	Immigration strongly reduces support of social policies.	5	2.6%
2	Immigration somewhat reduces support of social policies.	53	28.0%
3	Immigration has no effect on support of social policies.	77	40.7%
4	Immigration somewhat increases support of social policies.	11	5.8%
.a		43	22.8%

Housing?

Name:	belief_housing_3		
Variable label:	Housing		

Value	Label	Freq.	Percent
1	Immigration strongly reduces support of social policies.	6	3.2%
2	Immigration somewhat reduces support of social policies.	36	19.0%
3	Immigration has no effect on support of social policies.	86	45.5%
4	Immigration somewhat increases support of social policies.	17	9.0%
.a		44	23.3%

Active labor market programs?

Name:	belief_labour_3		
Variable label:	Active labor market programs		

Value	Label	Freq.	Percent
1	Immigration strongly reduces support of social policies.	5	2.6%
2	Immigration somewhat reduces support of social policies.	46	24.3%
3	Immigration has no effect on support of social policies.	76	40.2%
4	Immigration somewhat increases support of social policies.	19	10.1%
.a		43	22.8%

Health care?

Name:	belief_health_3		
Variable label:	Health care		

Value	Label	Freq.	Percent
1	Immigration strongly reduces support of social policies.	3	1.6%
2	Immigration somewhat reduces support of social policies.	24	12.7%
3	Immigration has no effect on support of social policies.	105	55.6%
4	Immigration somewhat increases support of social policies.	13	6.9%
.a		44	23.3%

You just indicated your beliefs about the effects of immigration on public support of social policies. How certain are you that your beliefs about this relationship are correct?

Name:	belief_certainty_3		
Variable label:	very uncertain - very certain		
Value	Label	Freq.	Percent
1	very uncertain	7	3.7%
2		25	13.2%
3		25	13.2%
4		27	14.3%
5		42	22.2%
6		15	7.9%
7	very certain	3	1.6%
.a		45	23.8%

WAVE 4: January 20th, 2019

To improve future crowdsourced research projects, we seek to understand your motivation to participate in the CRI. We list potential reasons that may or may not apply to you personally. Please indicate to which degree these statements apply to your motivation to participate in the CRI.

[battery]

I was very interested in the substantive topic.

Name:	v_88		
Variable label:	Interest		

Value	Label	Freq.	Percent
1	Reason does not apply to me apply at all	10	5.3%
2	Reason applies to me a little	30	15.9%
3	Neither nor	22	11.6%
4	Reason somewhat applies to me	51	27.0%
5	Reason applies to me a lot	45	23.8%
.a		31	16.4%

Colleagues asked me to join their team.

Name:	v_89		
Variable label:	Colleagues		

Value	Label	Freq.	Percent
1	Reason does not apply to me apply at all	63	33.3%
2	Reason applies to me a little	15	7.9%
3	Neither nor	4	2.1%
4	Reason somewhat applies to me	33	17.5%
5	Reason applies to me a lot	41	21.7%
.a		33	17.5%

The prospect of a scientific publication was appealing.

Name: v_90
Variable label: Scientific publication

Value	Label	Freq.	Percent
1	Reason does not apply to me apply at all	10	5.3%
2	Reason applies to me a little	19	10.1%
3	Neither nor	24	12.7%
4	Reason somewhat applies to me	66	34.9%
5	Reason applies to me a lot	38	20.1%
.a		32	16.9%

I expected the project to be an enjoyable experience.

Name: v_91
Variable label: Expected enjoyment

Value	Label	Freq.	Percent
1	Reason does not apply to me apply at all	2	1.1%
2	Reason applies to me a little	4	2.1%
3	Neither nor	15	7.9%
4	Reason somewhat applies to me	76	40.2%
5	Reason applies to me a lot	60	31.7%
.a		32	16.9%

I was very interested in the replication aspect of the project.

Name: v_93
Variable label: Replication

Value	Label	Freq.	Percent
1	Reason does not apply to me apply at all	2	1.1%
2	Reason applies to me a little	3	1.6%
3	Neither nor	4	2.1%
4	Reason somewhat applies to me	61	32.3%
5	Reason applies to me a lot	86	45.5%
.a		33	17.5%

I expected to learn and to develop as a researcher.

Name:	v_94
Variable label:	Develop as researcher

Value	Label	Freq.	Percent
1	Reason does not apply to me apply at all	2	1.1%
2	Reason applies to me a little	14	7.4%
3	Neither nor	19	10.1%
4	Reason somewhat applies to me	74	39.2%
5	Reason applies to me a lot	46	24.3%
.a		34	18.0%

The CRI seemed like a valuable addition to my CV.

Name:	v_95
Variable label:	CV

Value	Label	Freq.	Percent
1	Reason does not apply to me apply at all	31	16.4%
2	Reason applies to me a little	43	22.8%
3	Neither nor	43	22.8%
4	Reason somewhat applies to me	32	16.9%
5	Reason applies to me a lot	8	4.2%
.a		32	16.9%

I joined because I know one or more of the organizers.

Name:	v_96
Variable label:	Know organizers

Value	Label	Freq.	Percent
1	Reason does not apply to me apply at all	108	57.1%
2	Reason applies to me a little	9	4.8%
3	Neither nor	13	6.9%
4	Reason somewhat applies to me	19	10.1%
5	Reason applies to me a lot	8	4.2%
.a		32	16.9%

During the CRI many participants mentioned constraints to their work. How much did the following constraints prevent you from submitting your ideal work?

[battery]

Not enough time

Name:	v_98			
Variable label:	Not enough time			

Value	Label	Freq.	Percent
1	Did not constrain me	8	4.2%
2	Constrained me only a little	26	13.8%
3	Constrained me somewhat	51	27.0%
4	Constrained me considerably	71	37.6%
.a		33	17.5%

Inadequate materials (e.g., software or computing power)

Name:	v_99
Variable label:	Inadequate materials

Value	Label	Freq.	Percent
1	Did not constrain me	110	58.2%
2	Constrained me only a little	27	14.3%
3	Constrained me somewhat	13	6.9%
4	Constrained me considerably	7	3.7%
.a		32	16.9%

Not enough methods skills

Name:	v_100
Variable label:	Methods skills

Value	Label	Freq.	Percent
1	Did not constrain me	65	34.4%
2	Constrained me only a little	53	28.0%
3	Constrained me somewhat	30	15.9%
4	Constrained me considerably	9	4.8%
.a		32	16.9%

Not enough software programming skills

Name:	v_101
Variable label:	Software programming skills
Value label:	v_98

Value	Label	Freq.	Percent
1	Did not constrain me	110	58.2%
2	Constrained me only a little	35	18.5%
3	Constrained me somewhat	12	6.3%
4	Constrained me considerably	0	0.0%
.a		32	16.9%

Having strict deadlines was a problem for me

Name:	v_102
Variable label:	Strict deadlines

Value	Label	Freq.	Percent
1	Did not constrain me	40	21.2%
2	Constrained me only a little	49	25.9%
3	Constrained me somewhat	48	25.4%
4	Constrained me considerably	20	10.6%
.a		32	16.9%

Lastly, what is your gender?

Name:	v_110
Variable label:	Gender

Value	Label	Freq.	Percent
1	male	106	56.1%
2	female	50	26.5%
3	other	0	0.0%
.a		33	17.5%

IV. Model Ranking during Participant Survey, Wave 4

Model Descriptions for Ranking

Participants were randomly assigned models to rank in the 4th wave of our participant survey (see Supplementary Materials Appendix III. Participant Survey Codebook for details).

In this section, we ask you to review three research designs that were submitted by other CRI research teams. We kindly ask you to carefully read the research designs, each designated by the larger font below.

For each design, please indicate how confident you are that the respective research design is adequate for testing the hypothesis that 'immigration undermines social policy preferences' using ISSP data.

For each "Design", Participants were asked to score:

- Unconfident
- Rather unconfident
- Neither confident nor unconfident
- Rather confident
- Confident

Design code 1

BASIC MODEL FORM: Logistic regression. Includes listwise deletion of missing values.
DETAILED MODEL DESCRIPTION: A 'two-way fixed-effects' similar to that of Brady & Finnigan. Includes dummy variables for countries and years. Estimation Method: Maximum-likelihood (binomial/categorical).
ADDITIONAL MODEL SPECIFICATIONS: Includes some form of robust clustering.
MEASUREMENT of the SIX DEPENDENT VARIABLES: Dichotomized. Each DV estimated in separate models.
ISSP WAVES INCLUDED: 1996,2006. Countries Included: Australia, Canada, France, Germany, Ireland, Japan, New Zealand, Norway, Spain, Sweden, Switzerland, Great Britain, United States.
SPECIAL FEATURES of the COUNTRY SAMPLE: Includes only the original 13 countries used by Brady & Finnigan.
COUNTRY-LEVEL INDEPENDENT VARIABLES TESTED: Stock of foreign-born, Net migration (over a 1-year period), Employment rate, Social spending as a % of GDP.
INDIVIDUAL-LEVEL INDEPENDENT VARIABLES TESTED: Age, Age-squared, Sex, Employment status, Income, Educational attainment.
INTERACTIONS of VARIABLES TESTED: None.

Design code 2

BASIC MODEL FORM: Logistic regression. Includes listwise deletion of missing values.
DETAILED MODEL DESCRIPTION: Estimation Method: Maximum-likelihood (binomial/categorical).
ADDITIONAL MODEL SPECIFICATIONS: Includes some form of robust clustering.
MEASUREMENT of the SIX DEPENDENT VARIABLES: Dichotomized. Each DV estimated in separate models.
ISSP WAVES INCLUDED: 1996, 2006. Countries Included: Australia, Canada, France, Germany, Ireland, Japan, New Zealand, Norway, Spain, Sweden, Switzerland, Great Britain, United States.
SPECIAL FEATURES of the COUNTRY SAMPLE: Includes only the original 13 countries used by Brady & Finnigan.
COUNTRY-LEVEL INDEPENDENT VARIABLES TESTED: Stock of foreign-born, Net migration (over a 1-year period), Net migration (over a 10-year period), Employment rate, Social spending as a % of GDP, GDP, Change in GDP, Gini, Multiculturalism Policy Index (or equivalent).
INDIVIDUAL-LEVEL INDEPENDENT VARIABLES TESTED: Age, Age-squared, Sex, Employment status, Income, Educational attainment.
INTERACTIONS of VARIABLES TESTED: None.

Design code 3

BASIC MODEL FORM: Ordered logistic regression. Includes listwise deletion of missing values.
DETAILED MODEL DESCRIPTION: Multilevel random-effects model employed, defined as having random intercepts, fixed-slopes and unique error variance at each higher level. Heirarchical Levels: country.Categorical. Each DV estimated in separate models. Estimation Method: Bayesian.
ADDITIONAL MODEL SPECIFICATIONS: Includes some form of robust clustering.
MEASUREMENT of the SIX DEPENDENT VARIABLES: Categorical. Each DV estimated in separate models.
ISSP WAVES INCLUDED: 1996, 2006. Countries Included: Australia, Canada, France, Germany, Ireland, Japan, New Zealand, Norway, Spain, Sweden, Switzerland, Great Britain, United States.
SPECIAL FEATURES of the COUNTRY SAMPLE: Includes only the original 13 countries used by Brady & Finnigan.
COUNTRY-LEVEL INDEPENDENT VARIABLES TESTED: Stock of foreign-born, Net migration (over a 1-year period), Employment rate, Social spending as a % of GDP.
INDIVIDUAL-LEVEL INDEPENDENT VARIABLES TESTED: Age, Age-squared, Sex, Employment status, Educational attainment.
INTERACTIONS of VARIABLES TESTED: None.

Design code 4

BASIC MODEL FORM: Linear regression. Includes listwise deletion of missing values.
DETAILED MODEL DESCRIPTION: Multilevel random-effects model employed, defined as having random intercepts, fixed-slopes and unique error variance at each higher level. Heirarchical Levels: country-year. Estimation Method: Maximum-likelihood (linear).
ADDITIONAL MODEL SPECIFICATIONS: Bootstraps the small number of countries to obtain robust estimates (e.g., leave-one-out or jackknife estimator).
MEASUREMENT of the SIX DEPENDENT VARIABLES: Linear. Single DV estimated as a scale.
ISSP WAVES INCLUDED: 1985, 1990, 1996, 2006, 2016. Countries Included: Australia, Canada, France, Germany, Ireland, Japan, New Zealand, Norway, Spain, Sweden, Switzerland, Great Britain, United States.
SPECIAL FEATURES of the COUNTRY SAMPLE: Includes only the original 13 countries used by Brady & Finnigan.
COUNTRY-LEVEL INDEPENDENT VARIABLES TESTED: Stock of foreign-born, Percentage change in foreign-born stock (1-year), Unemployment rate, Change in unemployment rate (1-year), Social spending as a % of GDP, Change in social spending as a % of GDP, GDP, Change in GDP, Welfare state regime types.
INDIVIDUAL-LEVEL INDEPENDENT VARIABLES TESTED: Age, Age-squared, Sex, Employment status, Income, Educational attainment.
INTERACTIONS of VARIABLES TESTED: None.

Design code 5

BASIC MODEL FORM: Logistic regression. Includes listwise deletion of missing values.
DETAILED MODEL DESCRIPTION: Multilevel random-effects model employed, defined as having random intercepts, fixed-slopes and unique error variance at each higher level. Heirarchical Levels: country-year. Estimation Method: Maximum-likelihood (binomial/categorical).
ADDITIONAL MODEL SPECIFICATIONS: Bootstraps the small number of countries to obtain robust estimates (e.g., leave-one-out or jackknife estimator).
MEASUREMENT of the SIX DEPENDENT VARIABLES: Dichotomized. Each DV estimated in separate models.
ISSP WAVES INCLUDED: 1985, 1990, 1996, 2006, 2016. Countries Included: Australia, Canada, France, Germany, Ireland, Japan, New Zealand, Norway, Spain, Sweden, Switzerland, Great Britain, United States.
SPECIAL FEATURES of the COUNTRY SAMPLE: Includes only the original 13 countries used by Brady & Finnigan.
COUNTRY-LEVEL INDEPENDENT VARIABLES TESTED: Stock of foreign-born, Percentage change in foreign-born stock (1-year), Unemployment rate, Change in unemployment rate (1-year), Social spending as a % of GDP, Change in social spending as a % of GDP, GDP, Change in GDP, Welfare state regime types.
INDIVIDUAL-LEVEL INDEPENDENT VARIABLES TESTED: Age, Age-squared, Sex, Employment status, Income, Educational attainment.
INTERACTIONS of VARIABLES TESTED: None.

Design code 6

BASIC MODEL FORM: Logistic regression. Includes listwise deletion of missing values.
DETAILED MODEL DESCRIPTION: A 'two-way fixed-effects' similar to that of Brady & Finnigan. Includes dummy variables for countries and years. Estimation Method: Bayesian.
ADDITIONAL MODEL SPECIFICATIONS: Bootstraps the small number of countries to obtain robust estimates (e.g., leave-one-out or jackknife estimator).
MEASUREMENT of the SIX DEPENDENT VARIABLES: Dichotomized. Each DV estimated in separate models.
ISSP WAVES INCLUDED: 1996, 2006, 2016. Countries Included: Australia, Canada, France, Germany, Ireland, Japan, New Zealand, Norway, Spain, Sweden, Switzerland, Great Britain, United States.
SPECIAL FEATURES of the COUNTRY SAMPLE: Includes only the original 13 countries used by Brady & Finnigan.
COUNTRY-LEVEL INDEPENDENT VARIABLES TESTED: Stock of foreign-born, Employment rate, Social spending as a % of GDP.
INDIVIDUAL-LEVEL INDEPENDENT VARIABLES TESTED: Age, Age-squared, Sex, Employment status, Income, Educational attainment.
INTERACTIONS of VARIABLES TESTED: None.

Design code 7

BASIC MODEL FORM: Logistic regression. Includes listwise deletion of missing values.

DETAILED MODEL DESCRIPTION: Multilevel random-effects model employed, defined as having random intercepts, fixed-slopes and unique error variance at each higher level. Heirarchical Levels: country. Estimation Method: Maximum-likelihood (binomial/categorical).

ADDITIONAL MODEL SPECIFICATIONS: None.

MEASUREMENT of the SIX DEPENDENT VARIABLES: Dichotomized. Each DV estimated in separate models.

ISSP WAVES INCLUDED: 2006, 2016. Countries Included: Austria, Belgium, Canada, Chile, Croatia, Czechia, Denmark, Finland, France, Germany, Hungary, Iceland, Ireland, Israel, Japan, South Korea, Latvia, Lithuania, Netherlands, New Zealand, Norway, Poland, Portugal, Slovenia, Spain, Sweden, Switzerland, Great Britain, United States, South Africa.

SPECIAL FEATURES of the COUNTRY SAMPLE: Includes Eastern Europe.Includes all available countries with relevant measures.

COUNTRY-LEVEL INDEPENDENT VARIABLES TESTED: Stock of foreign-born, Employment rate, Stock of refugees, Change in refugee stock, Social spending as a % of GDP, GDP.

INDIVIDUAL-LEVEL INDEPENDENT VARIABLES TESTED: Age, Age-squared, Sex, Employment status, Income, Educational attainment.

INTERACTIONS of VARIABLES TESTED: None.

Design code 8

BASIC MODEL FORM: Ordered logistic regression. Includes listwise deletion of missing values.

DETAILED MODEL DESCRIPTION: A 'two-way fixed-effects' model similar to that of Brady & Finnigan with the addition of robust clustered standard errors. Includes dummy variables for countries and years.Categorical. Each DV estimated in separate models. Estimation Method: Maximum-likelihood (binomial/categorical).

ADDITIONAL MODEL SPECIFICATIONS: Includes some form of robust clustering.

MEASUREMENT of the SIX DEPENDENT VARIABLES: Categorical. Each DV estimated in separate models.

ISSP WAVES INCLUDED: 1996, 2006, 2016. Countries Included: Australia, Canada, Denmark, Finland, France, Germany, Ireland, Japan, New Zealand, Norway, Spain, Sweden, Switzerland, Great Britain, United States.

SPECIAL FEATURES of the COUNTRY SAMPLE:

COUNTRY-LEVEL INDEPENDENT VARIABLES TESTED: Stock of foreign-born, Net migration (over a 1-year period), Unemployment rate, Social spending as a % of GDP, GDP.

INDIVIDUAL-LEVEL INDEPENDENT VARIABLES TESTED: Age, Age-squared, Sex, Employment status, Income, Educational attainment.

INTERACTIONS of VARIABLES TESTED: None.

Design code 9

BASIC MODEL FORM: Linear regression.
DETAILED MODEL DESCRIPTION: Multilevel random-effects model employed, defined as having random intercepts, fixed-slopes and unique error variance at each higher level. Heirarchical Levels: country-year. Estimation Method: Maximum-likelihood (binomial/categorical).
ADDITIONAL MODEL SPECIFICATIONS: None.
MEASUREMENT of the SIX DEPENDENT VARIABLES: Linear. Single DV estimated as a scale.
ISSP WAVES INCLUDED: 1985, 1990, 1996, 2006, 2016. Countries Included: Australia, Canada, Finland, France, Germany, Ireland, Italy, Japan, New Zealand, Norway, Spain, Sweden, Switzerland, Great Britain, United States.
SPECIAL FEATURES of the COUNTRY SAMPLE:
COUNTRY-LEVEL INDEPENDENT VARIABLES TESTED: Stock of foreign-born, Net migration (over a 1-year period), Employment rate, Social spending as a % of GDP, Political conservatism of government (left-to-right), Decommodification index, Immigration Policy Index.
INDIVIDUAL-LEVEL INDEPENDENT VARIABLES TESTED: Age, Age-squared, Sex, Employment status, Income, Educational attainment, Marital status, Household size, Religious denomination, Religious attendance.
INTERACTIONS of VARIABLES TESTED: Net migration*Political Conservatism.

Design code 10

BASIC MODEL FORM: Linear regression. Includes listwise deletion of missing values.

DETAILED MODEL DESCRIPTION: Multilevel random-effects model employed, defined as having random intercepts, fixed-slopes and unique error variance at each higher level. Heirarchical Levels: country-year. Estimation Method: Maximum-likelihood (linear).

ADDITIONAL MODEL SPECIFICATIONS: None.

MEASUREMENT of the SIX DEPENDENT VARIABLES: Linear. Single DV estimated as a scale.

ISSP WAVES INCLUDED: 1996, 2006, 2016. Countries Included: Australia, Canada, France, Germany, Ireland, Israel, Japan, New Zealand, Norway, Spain, Sweden, Switzerland, Great Britain, United States.

SPECIAL FEATURES of the COUNTRY SAMPLE:

COUNTRY-LEVEL INDEPENDENT VARIABLES TESTED: Stock of foreign-born, Employment rate, Social spending as a % of GDP.

INDIVIDUAL-LEVEL INDEPENDENT VARIABLES TESTED: Age, Age-squared, Sex, Employment status, Educational attainment.

INTERACTIONS of VARIABLES TESTED: None.

Design code 11

BASIC MODEL FORM: Linear regression. Includes listwise deletion of missing values.

DETAILED MODEL DESCRIPTION: Multilevel random-effects model employed, defined as having random intercepts, fixed-slopes and unique error variance at each higher level. Heirarchical Levels: country-year. Estimation Method: Maximum-likelihood (binomial/categorical).

ADDITIONAL MODEL SPECIFICATIONS: None.

MEASUREMENT of the SIX DEPENDENT VARIABLES: Dichotomized. Each DV estimated in separate models.

ISSP WAVES INCLUDED: 1996, 2006, 2016. Countries Included: Australia, Canada, France, Germany, Ireland, Japan, New Zealand, Norway, Spain, Sweden, Switzerland, Great Britain, United States.

SPECIAL FEATURES of the COUNTRY SAMPLE: Includes only the original 13 countries used by Brady & Finnigan.

COUNTRY-LEVEL INDEPENDENT VARIABLES TESTED: Stock of foreign-born, Net migration (over a 1-year period), Percentage change in foreign-born stock (10-year), Employment rate, Social spending as a % of GDP, GDP.

INDIVIDUAL-LEVEL INDEPENDENT VARIABLES TESTED: Age, Age-squared, Sex, Employment status, Educational attainment, Subjective left-right self-placement.

INTERACTIONS of VARIABLES TESTED: Foreign-born stock*Net migration.

Design code 12

BASIC MODEL FORM: Linear regression. Includes listwise deletion of missing values.

DETAILED MODEL DESCRIPTION: Multilevel random-effects model employed, defined as having random intercepts, fixed-slopes and unique error variance at each higher level. Heirarchical Levels: country, year. Estimation Method: Maximum-likelihood (linear).

ADDITIONAL MODEL SPECIFICATIONS: None.

MEASUREMENT of the SIX DEPENDENT VARIABLES: Linear. Single DV estimated as a scale.

ISSP WAVES INCLUDED: 1985, 1990, 1996, 2006, 2016. Countries Included: Australia, Austria, Belgium, Canada, Denmark, Finland, France, Western Germany, Eastern Germany, Ireland, Italy, Japan, Netherlands, New Zealand, Norway, Spain, Sweden, Switzerland, Great Britain, United States.

SPECIAL FEATURES of the COUNTRY SAMPLE:

COUNTRY-LEVEL INDEPENDENT VARIABLES TESTED: Stock of foreign-born, Employment rate, Social spending as a % of GDP, Welfare state regime types.

INDIVIDUAL-LEVEL INDEPENDENT VARIABLES TESTED: Age, Age-squared, Sex, Employment status, Income, Educational attainment, Subjective left-right self-placement.

INTERACTIONS of VARIABLES TESTED: None.

Design code 13

BASIC MODEL FORM: Logistic regression. Includes listwise deletion of missing values.

DETAILED MODEL DESCRIPTION: Multilevel random-effects model employed, defined as having random intercepts, fixed-slopes and unique error variance at each higher level. Heirarchical Levels: country-year, country. Estimation Method: Maximum-likelihood (binomial/categorical).

ADDITIONAL MODEL SPECIFICATIONS: None.

MEASUREMENT of the SIX DEPENDENT VARIABLES: Dichotomized. Each DV estimated in separate models.

ISSP WAVES INCLUDED: 1996, 2006. Countries Included: Australia, Canada, France, Germany, Ireland, Japan, New Zealand, Norway, Spain, Sweden, Switzerland, Great Britain, United States.

SPECIAL FEATURES of the COUNTRY SAMPLE: Includes only the original 13 countries used by Brady & Finnigan.

COUNTRY-LEVEL INDEPENDENT VARIABLES TESTED: Stock of foreign-born, Net migration (over a 1-year period), Employment rate, Social spending as a % of GDP.

INDIVIDUAL-LEVEL INDEPENDENT VARIABLES TESTED: Age, Age-squared, Sex, Employment status, Income, Educational attainment.

INTERACTIONS of VARIABLES TESTED: None.

Design code 14

BASIC MODEL FORM: Linear regression. Includes listwise deletion of missing values.

DETAILED MODEL DESCRIPTION: Multilevel random-effects model employed, defined as having random intercepts, fixed-slopes and unique error variance at each higher level. Heirarchical Levels: country-year, Multilevel structural equation model with random intercepts, fixed-slopes and unique error variance at each higher level. Estimation Method: Bayesian.

ADDITIONAL MODEL SPECIFICATIONS: None.

MEASUREMENT of the SIX DEPENDENT VARIABLES: Linear. Single DV estimated as a scale.

ISSP WAVES INCLUDED: 1996, 2006, 2016. Countries Included: Australia, Canada, France, Germany, Ireland, Japan, New Zealand, Norway, Spain, Sweden, Switzerland, Great Britain, United States.

SPECIAL FEATURES of the COUNTRY SAMPLE: Includes only the original 13 countries used by Brady & Finnigan.

COUNTRY-LEVEL INDEPENDENT VARIABLES TESTED: Stock of foreign-born, Net migration (over a 1-year period), Employment rate, Social spending as a % of GDP, GDP.

INDIVIDUAL-LEVEL INDEPENDENT VARIABLES TESTED: Age, Age-squared, Sex, Educational attainment.

INTERACTIONS of VARIABLES TESTED: None.

Design code 15

BASIC MODEL FORM: Logistic regression. Includes listwise deletion of missing values.
DETAILED MODEL DESCRIPTION: A 'two-way fixed-effects' similar to that of Brady & Finnigan. Includes dummy variables for countries and years. Estimation Method: Maximum-likelihood (binomial/categorical).
ADDITIONAL MODEL SPECIFICATIONS: Bootstraps the small number of countries to obtain robust estimates (e.g., leave-one-out or jackknife estimator).
MEASUREMENT of the SIX DEPENDENT VARIABLES: Dichotomized. Each DV estimated in separate models.
ISSP WAVES INCLUDED: 1996, 2006, 2016. Countries Included: Australia, Canada, Croatia, Czechia, Denmark, Finland, France, Germany, Hungary, Ireland, Japan, Latvia, New Zealand, Norway, Poland, Slovenia, Spain, Sweden, Switzerland, Great Britain, United States.
SPECIAL FEATURES of the COUNTRY SAMPLE: Includes Eastern Europe.
COUNTRY-LEVEL INDEPENDENT VARIABLES TESTED: Stock of foreign-born, Net migration (over a 1-year period),
INDIVIDUAL-LEVEL INDEPENDENT VARIABLES TESTED: Age, Age-squared, Sex, Income, Educational attainment.
INTERACTIONS of VARIABLES TESTED: Net migration*Income.

Design code 16

BASIC MODEL FORM: Linear regression. Includes listwise deletion of missing values.
DETAILED MODEL DESCRIPTION: A 'two-way fixed-effects' similar to that of Brady & Finnigan. Includes dummy variables for countries and years. Estimation Method: Ordinary least squares.
ADDITIONAL MODEL SPECIFICATIONS: None.
MEASUREMENT of the SIX DEPENDENT VARIABLES: Linear. Single DV estimated as a scale.
ISSP WAVES INCLUDED: 1996, 2006. Countries Included: Australia, Canada, Czechia, France, Germany, Ireland, Japan, Latvia, New Zealand, Norway, Poland, Slovenia, Spain, Sweden, Switzerland, Great Britain, United States.
SPECIAL FEATURES of the COUNTRY SAMPLE:
COUNTRY-LEVEL INDEPENDENT VARIABLES TESTED: Stock of foreign-born, Net migration (over a 1-year period), Social spending as a % of GDP, GDP.
INDIVIDUAL-LEVEL INDEPENDENT VARIABLES TESTED: Age, Age-squared, Sex, Employment status, Income, Educational attainment.
INTERACTIONS of VARIABLES TESTED: None.

Design code 17

BASIC MODEL FORM: Linear regression. Includes listwise deletion of missing values.
DETAILED MODEL DESCRIPTION: A 'two-way fixed-effects' similar to that of Brady & Finnigan. Includes dummy variables for countries and years. Estimation Method: Ordinary least squares.
ADDITIONAL MODEL SPECIFICATIONS: None.
MEASUREMENT of the SIX DEPENDENT VARIABLES: Linear. Single DV estimated as a scale.
ISSP WAVES INCLUDED: 1996, 2006. Countries Included: Australia, Canada, Czechia, France, Germany, Ireland, Japan, Latvia, New Zealand, Norway, Poland, Slovenia, Spain, Sweden, Switzerland, Great Britain, United States.
SPECIAL FEATURES of the COUNTRY SAMPLE:
COUNTRY-LEVEL INDEPENDENT VARIABLES TESTED: Stock of foreign-born, Net migration (over a 1-year period), Unemployment rate, Social spending as a % of GDP, GDP.
INDIVIDUAL-LEVEL INDEPENDENT VARIABLES TESTED: Age, Age-squared, Sex, Employment status, Income, Educational attainment.
INTERACTIONS of VARIABLES TESTED: None.

Design code 18

BASIC MODEL FORM: Linear regression. Includes listwise deletion of missing values.
DETAILED MODEL DESCRIPTION: A 'two-way fixed-effects' similar to that of Brady & Finnigan. Includes dummy variables for countries and years. Estimation Method: Ordinary least squares.
ADDITIONAL MODEL SPECIFICATIONS: None.
MEASUREMENT of the SIX DEPENDENT VARIABLES: Linear. Single DV estimated as a scale.
ISSP WAVES INCLUDED: 1996, 2006, 2016. Countries Included: Australia, Canada, France, Western Germany, Eastern Germany, Ireland, Italy, Netherlands, New Zealand, Norway, Spain, Sweden, Switzerland, Great Britain, United States.
SPECIAL FEATURES of the COUNTRY SAMPLE: Includes only the original 13 countries used by Brady & Finnigan.
COUNTRY-LEVEL INDEPENDENT VARIABLES TESTED: Stock of foreign-born, Net migration (over a 1-year period), Percentage change in foreign-born stock (10-year), Employment rate, Unemployment rate, Social spending as a % of GDP, Gini, Welfare state regime types, Trade union coverage, Total country population.
INDIVIDUAL-LEVEL INDEPENDENT VARIABLES TESTED: Age, Age-squared, Sex, Employment status, Income, Educational attainment, Household size, Religious attendance, Public sector employment, Urban/rural, Subjective preference for cutting government spending, Subjective preference for decreasing taxes, Respondent interested in politics.
INTERACTIONS of VARIABLES TESTED: None.

Design code 19

BASIC MODEL FORM: Linear regression. Includes listwise deletion of missing values.
DETAILED MODEL DESCRIPTION: A 'two-way fixed-effects' similar to that of Brady & Finnigan. Includes dummy variables for countries and years. Estimation Method: Ordinary least squares.
ADDITIONAL MODEL SPECIFICATIONS: None.
MEASUREMENT of the SIX DEPENDENT VARIABLES: Dichotomized. Each DV estimated in separate models. Linear. Single DV estimated as a scale.
ISSP WAVES INCLUDED: 1996, 2006, 2016. Countries Included: Australia, France, Germany, Japan, New Zealand, Norway, Spain, Sweden, Switzerland, Great Britain, United States.
SPECIAL FEATURES of the COUNTRY SAMPLE:
COUNTRY-LEVEL INDEPENDENT VARIABLES TESTED: Stock of foreign-born, Net migration (over a 1-year period), Percentage change in foreign-born stock (10-year), Social spending as a % of GDP, Change in social spending as a % of GDP, GDP, Gini, Multiculturalism Policy Index (or equivalent).
INDIVIDUAL-LEVEL INDEPENDENT VARIABLES TESTED: Age, Age-squared, Sex, Employment status, Income, Occupational class, Educational attainment, Subjective left-right self-placement, Political trust.
INTERACTIONS of VARIABLES TESTED: None.

Design code 20

BASIC MODEL FORM: Linear regression. Includes listwise deletion of missing values.
DETAILED MODEL DESCRIPTION: Estimation Method: Ordinary least squares.
ADDITIONAL MODEL SPECIFICATIONS: Includes some form of robust clustering.
MEASUREMENT of the SIX DEPENDENT VARIABLES: Dichotomized. Each DV estimated in separate models. Linear. Single DV estimated as a scale.
ISSP WAVES INCLUDED: 2016. Countries Included: Belgium, Denmark, Finland, France, Germany, Norway, Spain, Sweden, Switzerland, Great Britain.
SPECIAL FEATURES of the COUNTRY SAMPLE:
COUNTRY-LEVEL INDEPENDENT VARIABLES TESTED: Employment rate, Social spending as a % of GDP, Multiculturalism Policy Index (or equivalent), Welfare state regime types, Subjective foreign-born rate (taken from other surveys).
INDIVIDUAL-LEVEL INDEPENDENT VARIABLES TESTED: Age, Age-squared, Sex, Employment status, Educational attainment, Marital status, Household size, Religious attendance, Urban/rural.
INTERACTIONS of VARIABLES TESTED: None.

Design code 21

BASIC MODEL FORM: Logistic regression. Includes listwise deletion of missing values.
DETAILED MODEL DESCRIPTION: A 'two-way fixed-effects' model similar to that of Brady & Finnigan with the addition of robust clustered standard errors. Includes dummy variables for countries and years.country. Estimation Method: Maximum-likelihood (binomial/categorical).
ADDITIONAL MODEL SPECIFICATIONS: Includes some form of robust clustering.Bootstraps the small number of countries to obtain robust estimates (e.g., leave-one-out or jackknife estimator).
MEASUREMENT of the SIX DEPENDENT VARIABLES: Dichotomized. Each DV estimated in separate models.
ISSP WAVES INCLUDED: 1996, 2006. Countries Included: Australia, Canada, France, Germany, Ireland, Japan, New Zealand, Norway, Spain, Sweden, Switzerland, Great Britain, United States.
SPECIAL FEATURES of the COUNTRY SAMPLE: Includes only the original 13 countries used by Brady & Finnigan.
COUNTRY-LEVEL INDEPENDENT VARIABLES TESTED: Stock of foreign-born, Net migration (over a 1-year period), Employment rate, Social spending as a % of GDP.
INDIVIDUAL-LEVEL INDEPENDENT VARIABLES TESTED: Age, Age-squared, Sex, Employment status, Income, Educational attainment.
INTERACTIONS of VARIABLES TESTED: None.

Design code 22

BASIC MODEL FORM: Linear regression. Includes listwise deletion of missing values.

DETAILED MODEL DESCRIPTION: Multilevel random-effects model employed, defined as having random intercepts, fixed-slopes and unique error variance at each higher level.Multilevel fixed-effects model, defined as a longitudinal model with random-slopes for each higher level unit over time. Heirarchical Levels: country, year. Estimation Method: Maximum-likelihood (linear).

ADDITIONAL MODEL SPECIFICATIONS: None.

MEASUREMENT of the SIX DEPENDENT VARIABLES: Linear. Single DV estimated as a scale.

ISSP WAVES INCLUDED: 1996, 2006, 2016. Countries Included: Australia, Canada, Croatia, Czechia, Denmark, Finland, France, Germany, Hungary, Ireland, Japan, Latvia, New Zealand, Norway, Poland, Slovenia, Spain, Sweden, Switzerland, Great Britain, United States.

SPECIAL FEATURES of the COUNTRY SAMPLE: Includes Eastern Europe.

COUNTRY-LEVEL INDEPENDENT VARIABLES TESTED: Stock of foreign-born, Net migration (over a 1-year period), Unemployment rate, Social spending as a % of GDP, Gini.

INDIVIDUAL-LEVEL INDEPENDENT VARIABLES TESTED: Age, Age-squared, Sex, Employment status, Income, Educational attainment, Subjective left-right self-placement.

INTERACTIONS of VARIABLES TESTED: None.

Design code 23

BASIC MODEL FORM: Logistic regression. Includes listwise deletion of missing values.
DETAILED MODEL DESCRIPTION: A 'two-way fixed-effects' similar to that of Brady & Finnigan. Includes dummy variables for countries and years. Estimation Method: Maximum-likelihood (binomial/categorical).
ADDITIONAL MODEL SPECIFICATIONS: None.
MEASUREMENT of the SIX DEPENDENT VARIABLES: Dichotomized. Each DV estimated in separate models.
ISSP WAVES INCLUDED: 1996, 2006, 2016. Countries Included: Australia, Canada, Chile, Czechia, Denmark, Finland, France, Germany, Hungary, Iceland, Ireland, Japan, South Korea, Latvia, Lithuania, Netherlands, New Zealand, Norway, Philippines, Poland, Portugal, Russia, Slovenia, Spain, Sweden, Switzerland, Great Britain, United States, South Africa, Taiwan, Uruguay, Venezuela.
SPECIAL FEATURES of the COUNTRY SAMPLE: Includes Eastern Europe.Includes all available countries with relevant measures.
COUNTRY-LEVEL INDEPENDENT VARIABLES TESTED: Stock of foreign-born, Net migration (over a 1-year period), Unemployment rate,
INDIVIDUAL-LEVEL INDEPENDENT VARIABLES TESTED: Age, Sex, Employment status, Income, Educational attainment.
INTERACTIONS of VARIABLES TESTED: None.

Design code 24

BASIC MODEL FORM: Logistic regression. Includes listwise deletion of missing values.
DETAILED MODEL DESCRIPTION: A 'two-way fixed-effects' model similar to that of Brady & Finnigan with the addition of robust clustered standard errors. Includes dummy variables for countries and years. Estimation Method: Maximum-likelihood (binomial/categorical).
ADDITIONAL MODEL SPECIFICATIONS: Includes some form of robust clustering.Bootstraps the small number of countries to obtain robust estimates (e.g., leave-one-out or jackknife estimator).
MEASUREMENT of the SIX DEPENDENT VARIABLES: Dichotomized. Each DV estimated in separate models.
ISSP WAVES INCLUDED: 1985, 1990, 1996, 2006, 2016. Countries Included: Australia, Canada, Croatia, Czechia, Denmark, Finland, France, Germany, Hungary, Ireland, Italy, Japan, Latvia, New Zealand, Norway, Poland, Russia, Slovakia, Slovenia, Spain, Sweden, Switzerland, Great Britain, United States.
SPECIAL FEATURES of the COUNTRY SAMPLE: Includes Eastern Europe.Includes all available countries with relevant measures.
COUNTRY-LEVEL INDEPENDENT VARIABLES TESTED: Stock of foreign-born, Net migration (over a 1-year period), Employment rate, Unemployment rate, Social spending as a % of GDP, GDP.
INDIVIDUAL-LEVEL INDEPENDENT VARIABLES TESTED: Age, Age-squared, Sex, Employment status, Income, Educational attainment.
INTERACTIONS of VARIABLES TESTED: Foreign-born stock*Net migration, Net migration*Individual educational attainment, Foreign-born stock*Individual educational attainment.

Design code 25

BASIC MODEL FORM: Logistic regression. Includes listwise deletion of missing values.

DETAILED MODEL DESCRIPTION: A 'two-way fixed-effects' model similar to that of Brady & Finnigan with the addition of robust clustered standard errors. Includes dummy variables for countries and years. Estimation Method: Maximum-likelihood (binomial/categorical).

ADDITIONAL MODEL SPECIFICATIONS: Includes some form of robust clustering.

MEASUREMENT of the SIX DEPENDENT VARIABLES: Dichotomized. Each DV estimated in separate models. Linear. Single DV estimated as a scale.

ISSP WAVES INCLUDED: 1996, 2006. Countries Included: Australia, Canada, France, Germany, Ireland, Japan, New Zealand, Norway, Spain, Sweden, Switzerland, Great Britain, United States.

SPECIAL FEATURES of the COUNTRY SAMPLE: Includes only the original 13 countries used by Brady & Finnigan.

COUNTRY-LEVEL INDEPENDENT VARIABLES TESTED: Stock of foreign-born, Employment rate, Social spending as a % of GDP, Socio-cultural proximity scale to immigrants (on average).

INDIVIDUAL-LEVEL INDEPENDENT VARIABLES TESTED: Age, Age-squared, Sex, Employment status, Income, Educational attainment.

INTERACTIONS of VARIABLES TESTED: Foreign-born stock*Unemployment, Foreign-born stock*Urban/rural, Foreign-born stock*Income, Immigration measures squared.

Design code 26

BASIC MODEL FORM: Logistic regression. Includes listwise deletion of missing values.
DETAILED MODEL DESCRIPTION: Multilevel random-effects model employed, defined as having random intercepts, fixed-slopes and unique error variance at each higher level. Heirarchical Levels: country-year, country. Estimation Method: Maximum-likelihood (binomial/categorical).
ADDITIONAL MODEL SPECIFICATIONS: Bootstraps the small number of countries to obtain robust estimates (e.g., leave-one-out or jackknife estimator).
MEASUREMENT of the SIX DEPENDENT VARIABLES: Dichotomized. Each DV estimated in separate models. Linear. Single DV estimated as a scale.
ISSP WAVES INCLUDED: 1996, 2006. Countries Included: Australia, Belgium, Canada, Chile, Czechia, Denmark, Finland, France, Germany, Hungary, Iceland, Ireland, Israel, Italy, Japan, South Korea, Latvia, Netherlands, New Zealand, Norway, Poland, Portugal, Slovakia, Slovenia, Spain, Sweden, Switzerland, Great Britain, United States.
SPECIAL FEATURES of the COUNTRY SAMPLE: Includes Eastern Europe.Includes all available countries with relevant measures.
COUNTRY-LEVEL INDEPENDENT VARIABLES TESTED: Stock of foreign-born, Employment rate, Social spending as a % of GDP, Ethnic fractionalization/Herfindahl index.
INDIVIDUAL-LEVEL INDEPENDENT VARIABLES TESTED: Age, Age-squared, Sex, Employment status, Income, Educational attainment.
INTERACTIONS of VARIABLES TESTED: Foreign-born stock*Ethnic fractionalization index.

Design code 27

BASIC MODEL FORM: Linear regression. Includes listwise deletion of missing values.
DETAILED MODEL DESCRIPTION: Multilevel random-effects model employed, defined as having random intercepts, fixed-slopes and unique error variance at each higher level. Heirarchical Levels: country, year. Estimation Method: Maximum-likelihood (binomial/categorical).
ADDITIONAL MODEL SPECIFICATIONS: None.
MEASUREMENT of the SIX DEPENDENT VARIABLES: Dichotomized. Each DV estimated in separate models.
ISSP WAVES INCLUDED: 1996, 2006, 2016. Countries Included: Australia, Canada, France, Germany, Ireland, Japan, New Zealand, Norway, Spain, Sweden, Switzerland, Great Britain, United States.
SPECIAL FEATURES of the COUNTRY SAMPLE: Includes only the original 13 countries used by Brady & Finnigan.
COUNTRY-LEVEL INDEPENDENT VARIABLES TESTED: Stock of foreign-born, Net migration (over a 1-year period), Employment rate, Social spending as a % of GDP.
INDIVIDUAL-LEVEL INDEPENDENT VARIABLES TESTED: Age, Age-squared, Sex, Employment status, Income, Educational attainment, Household size, Religious attendance, Urban/rural.
INTERACTIONS of VARIABLES TESTED: None.

Design code 28

BASIC MODEL FORM: Linear regression. Includes listwise deletion of missing values.
DETAILED MODEL DESCRIPTION: A 'two-way fixed-effects' model similar to that of Brady & Finnigan with the addition of robust clustered standard errors. Includes dummy variables for countries and years. Estimation Method: Ordinary least squares.
ADDITIONAL MODEL SPECIFICATIONS: Includes some form of robust clustering.Employs survey weighting as provided by the ISSP.
MEASUREMENT of the SIX DEPENDENT VARIABLES: Linear. Each DV estimated in separate models.
ISSP WAVES INCLUDED: 1996, 2006, 2016. Countries Included: Australia, France, Germany, Japan, New Zealand, Norway, Spain, Sweden, Switzerland, Great Britain, United States.
SPECIAL FEATURES of the COUNTRY SAMPLE:
COUNTRY-LEVEL INDEPENDENT VARIABLES TESTED: Stock of foreign-born, Unemployment rate, Social spending as a % of GDP, Stock of non-Western immigrants.
INDIVIDUAL-LEVEL INDEPENDENT VARIABLES TESTED: Age, Age-squared, Sex, Employment status, Educational attainment, Marital status.
INTERACTIONS of VARIABLES TESTED: None.

Design code 29

BASIC MODEL FORM: Linear regression. Includes listwise deletion of missing values.
DETAILED MODEL DESCRIPTION: A 'two-way fixed-effects' model similar to that of Brady & Finnigan with the addition of robust clustered standard errors. Includes dummy variables for countries and years. Estimation Method: Ordinary least squares.
ADDITIONAL MODEL SPECIFICATIONS: Includes some form of robust clustering.Employs survey weighting as provided by the ISSP.
MEASUREMENT of the SIX DEPENDENT VARIABLES: Linear. Single DV estimated as a scale.
ISSP WAVES INCLUDED: 1996, 2006, 2016. Countries Included: Australia, France, Germany, Japan, New Zealand, Norway, Spain, Sweden, Switzerland, Great Britain, United States.
SPECIAL FEATURES of the COUNTRY SAMPLE:
COUNTRY-LEVEL INDEPENDENT VARIABLES TESTED: Stock of foreign-born, Unemployment rate, Social spending as a % of GDP, Stock of non-Western immigrants.
INDIVIDUAL-LEVEL INDEPENDENT VARIABLES TESTED: Age, Age-squared, Sex, Employment status, Educational attainment, Marital status.
INTERACTIONS of VARIABLES TESTED: None.

Design code 30

BASIC MODEL FORM: Linear regression. Includes listwise deletion of missing values.

DETAILED MODEL DESCRIPTION: Multilevel random-effects model employed, defined as having random intercepts, fixed-slopes and unique error variance at each higher level. Heirarchical Levels: country. Estimation Method: Maximum-likelihood (linear).

ADDITIONAL MODEL SPECIFICATIONS: None.

MEASUREMENT of the SIX DEPENDENT VARIABLES: Linear. Each DV estimated in separate models.

ISSP WAVES INCLUDED: 1996, 2006. Countries Included: Australia, Canada, France, Germany, Ireland, Japan, New Zealand, Norway, Spain, Sweden, Switzerland, Great Britain, United States.

SPECIAL FEATURES of the COUNTRY SAMPLE: Includes only the original 13 countries used by Brady & Finnigan.

COUNTRY-LEVEL INDEPENDENT VARIABLES TESTED: Stock of foreign-born, Employment rate, Social spending as a % of GDP.

INDIVIDUAL-LEVEL INDEPENDENT VARIABLES TESTED: Age, Age-squared, Sex, Employment status, Income, Educational attainment.

INTERACTIONS of VARIABLES TESTED: None.

Design code 31

BASIC MODEL FORM: Linear regression. Includes listwise deletion of missing values.

DETAILED MODEL DESCRIPTION: Multilevel random-effects model employed, defined as having random intercepts, fixed-slopes and unique error variance at each higher level. Heirarchical Levels: country. Estimation Method: Maximum-likelihood (linear).

ADDITIONAL MODEL SPECIFICATIONS: None.

MEASUREMENT of the SIX DEPENDENT VARIABLES: Linear. Single DV estimated as a scale.

ISSP WAVES INCLUDED: 1990, 1996, 2006, 2016. Countries Included: Australia, Canada, Chile, Croatia, Czechia, Denmark, Finland, France, Germany, Hungary, Ireland, Israel, Italy, Japan, Latvia, New Zealand, Norway, Poland, Russia, Slovenia, Spain, Sweden, Switzerland, Great Britain, United States, South Africa, Taiwan, Venezuela.

SPECIAL FEATURES of the COUNTRY SAMPLE: Includes Eastern Europe.Includes all available countries with relevant measures.

COUNTRY-LEVEL INDEPENDENT VARIABLES TESTED: Stock of foreign-born, Percentage change in foreign-born stock (1-year), Employment rate, Social spending as a % of GDP, Multiculturalism Policy Index (or equivalent).

INDIVIDUAL-LEVEL INDEPENDENT VARIABLES TESTED: Age, Age-squared, Sex, Employment status, Income, Occupational status, Educational attainment.

INTERACTIONS of VARIABLES TESTED: None.

Design code 32

BASIC MODEL FORM: Linear regression. Includes listwise deletion of missing values.
DETAILED MODEL DESCRIPTION: Multilevel random-effects model employed, defined as having random intercepts, fixed-slopes and unique error variance at each higher level. Heirarchical Levels: country-year, country, Multilevel structural equation model with random intercepts, fixed-slopes and unique error variance at each higher level. Estimation Method: Bayesian.
ADDITIONAL MODEL SPECIFICATIONS: None.
MEASUREMENT of the SIX DEPENDENT VARIABLES: Linear. Single DV estimated as a scale.
ISSP WAVES INCLUDED: 1996, 2006, 2016. Countries Included: Australia, Canada, France, Germany, Ireland, Japan, New Zealand, Norway, Spain, Sweden, Switzerland, Great Britain, United States.
SPECIAL FEATURES of the COUNTRY SAMPLE: Includes only the original 13 countries used by Brady & Finnigan.
COUNTRY-LEVEL INDEPENDENT VARIABLES TESTED: Stock of foreign-born, Employment rate, Social spending as a % of GDP.
INDIVIDUAL-LEVEL INDEPENDENT VARIABLES TESTED: Age, Age-squared, Sex, Employment status, Income, Educational attainment.
INTERACTIONS of VARIABLES TESTED: None.

Design code 33

BASIC MODEL FORM: Logistic regression. Includes listwise deletion of missing values.
DETAILED MODEL DESCRIPTION: A 'two-way fixed-effects' model similar to that of Brady & Finnigan with the addition of robust clustered standard errors. Includes dummy variables for countries and years.country-year. Estimation Method: Maximum-likelihood (binomial/categorical).
ADDITIONAL MODEL SPECIFICATIONS: Includes some form of robust clustering.
MEASUREMENT of the SIX DEPENDENT VARIABLES: Dichotomized. Each DV estimated in separate models.
ISSP WAVES INCLUDED: 1996, 2006, 2016. Countries Included: Australia, Canada, France, Western Germany, Eastern Germany, Ireland, Japan, New Zealand, Norway, Spain, Sweden, Switzerland, Great Britain, United States.
SPECIAL FEATURES of the COUNTRY SAMPLE: Includes only the original 13 countries used by Brady & Finnigan.
COUNTRY-LEVEL INDEPENDENT VARIABLES TESTED: Stock of foreign-born, Employment rate, Social spending as a % of GDP.
INDIVIDUAL-LEVEL INDEPENDENT VARIABLES TESTED: Age, Age-squared, Sex, Employment status, Income, Educational attainment.
INTERACTIONS of VARIABLES TESTED: None.

Design code 34

BASIC MODEL FORM: Linear regression. Includes listwise deletion of missing values.
DETAILED MODEL DESCRIPTION: Multilevel random-effects model employed, defined as having random intercepts, fixed-slopes and unique error variance at each higher level. Heirarchical Levels: country, year. Estimation Method: Maximum-likelihood (linear).
ADDITIONAL MODEL SPECIFICATIONS: None.
MEASUREMENT of the SIX DEPENDENT VARIABLES: Linear. Single DV estimated as a scale.
ISSP WAVES INCLUDED: 1985, 1990, 1996, 2006, 2016. Countries Included: Australia, Canada, Czechia, Denmark, Finland, France, Germany, Hungary, Ireland, Italy, Japan, New Zealand, Norway, Poland, Spain, Sweden, Switzerland, Great Britain, United States.
SPECIAL FEATURES of the COUNTRY SAMPLE: Includes only the original 17 countries analyzed by Brady & Finnigan.
COUNTRY-LEVEL INDEPENDENT VARIABLES TESTED: Stock of foreign-born, Percentage change in foreign-born stock (1-year), Employment rate, Social spending as a % of GDP, GDP.
INDIVIDUAL-LEVEL INDEPENDENT VARIABLES TESTED: Age, Age-squared, Sex, Employment status, Income, Educational attainment.
INTERACTIONS of VARIABLES TESTED: None.

Design code 35

BASIC MODEL FORM: Linear regression. Includes listwise deletion of missing values.
DETAILED MODEL DESCRIPTION: Estimation Method: Ordinary least squares.
ADDITIONAL MODEL SPECIFICATIONS: Includes some form of robust clustering.Employs survey weighting as provided by the ISSP.
MEASUREMENT of the SIX DEPENDENT VARIABLES: Linear. Single DV estimated as a scale.
ISSP WAVES INCLUDED: 1985, 1990, 1996, 2006, 2016. Countries Included: Australia, Canada, Croatia, Czechia, Denmark, Finland, France, Germany, Hungary, Ireland, Japan, Latvia, New Zealand, Norway, Poland, Slovenia, Spain, Sweden, Switzerland, Great Britain, United States.
SPECIAL FEATURES of the COUNTRY SAMPLE: Includes Eastern Europe.
COUNTRY-LEVEL INDEPENDENT VARIABLES TESTED: Stock of foreign-born, Percentage change in foreign-born stock (10-year), Unemployment rate, Foreign-born educational attainment rate,Change in foreign-born educational attainment rate, GDP, Gini, Welfare state regime types, Stock of non-Western immigrants.
INDIVIDUAL-LEVEL INDEPENDENT VARIABLES TESTED: Age, Age-squared, Sex, Income, Occupational status, Educational attainment, Religious attendance, Foreign-born, Subjective left-right self-placement, Political trust.
INTERACTIONS of VARIABLES TESTED: None.

Design code 36

BASIC MODEL FORM: Linear regression. Includes listwise deletion of missing values.
DETAILED MODEL DESCRIPTION: A 'two-way fixed-effects' similar to that of Brady & Finnigan. Includes dummy variables for countries and years. Estimation Method: Maximum-likelihood (linear).
ADDITIONAL MODEL SPECIFICATIONS: None.
MEASUREMENT of the SIX DEPENDENT VARIABLES: Linear. Single DV estimated as a scale. Each DV is first dichotomized and then used to consturct a latent scale.
ISSP WAVES INCLUDED: 2006, 2016. Countries Included: Australia, Denmark, Finland, France, Germany, New Zealand, Norway, Spain, Sweden, Switzerland, Great Britain, United States.
SPECIAL FEATURES of the COUNTRY SAMPLE:
COUNTRY-LEVEL INDEPENDENT VARIABLES TESTED: Stock of foreign-born, Net migration (over a 1-year period), Employment rate, Foreign-born unemployment rate, Change in foreign-born unemployment rate, Social spending as a % of GDP.
INDIVIDUAL-LEVEL INDEPENDENT VARIABLES TESTED: Age, Age-squared, Sex, Employment status, Income, Educational attainment.
INTERACTIONS of VARIABLES TESTED: Foreign-born stock*Unemployment.

Design code 37

BASIC MODEL FORM: Linear regression. Includes listwise deletion of missing values.
DETAILED MODEL DESCRIPTION: A 'two-way fixed-effects' similar to that of Brady & Finnigan. Includes dummy variables for countries and years. Estimation Method: Ordinary least squares.
ADDITIONAL MODEL SPECIFICATIONS: None.
MEASUREMENT of the SIX DEPENDENT VARIABLES: Linear. Single DV estimated as a scale.
ISSP WAVES INCLUDED: 1996, 2006. Countries Included: Australia, Canada, France, Germany, Ireland, Japan, New Zealand, Norway, Spain, Sweden, Switzerland, Great Britain, United States.
SPECIAL FEATURES of the COUNTRY SAMPLE: Includes only the original 13 countries used by Brady & Finnigan.
COUNTRY-LEVEL INDEPENDENT VARIABLES TESTED: Stock of foreign-born, Net migration (over a 1-year period), Employment rate, Social spending as a % of GDP.
INDIVIDUAL-LEVEL INDEPENDENT VARIABLES TESTED: Age, Age-squared, Sex, Employment status, Income, Educational attainment.
INTERACTIONS of VARIABLES TESTED: None.

Design code 38

BASIC MODEL FORM: Linear regression. Engages in multiple imputation.
DETAILED MODEL DESCRIPTION: Multilevel random-effects model employed, defined as having random intercepts, fixed-slopes and unique error variance at each higher level. Heirarchical Levels: country-year, country, Multilevel structural equation model with random intercepts, fixed-slopes and unique error variance at each higher level. Estimation Method: Maximum-likelihood (linear).
ADDITIONAL MODEL SPECIFICATIONS: Employs survey weighting as provided by the ISSP.
MEASUREMENT of the SIX DEPENDENT VARIABLES: Linear. Single DV estimated as a scale.
ISSP WAVES INCLUDED: 2006, 2016. Countries Included: Australia, Canada, Denmark, Finland, France, Germany, Ireland, Japan, Netherlands, New Zealand, Norway, Spain, Sweden, Switzerland, Great Britain, United States.
SPECIAL FEATURES of the COUNTRY SAMPLE:
COUNTRY-LEVEL INDEPENDENT VARIABLES TESTED: Stock of foreign-born, Net migration (over a 1-year period), Employment rate, Social spending as a % of GDP, GDP, Welfare state regime types, Stock of non-Western immigrants.
INDIVIDUAL-LEVEL INDEPENDENT VARIABLES TESTED: Age, Age-squared, Sex, Employment status, Income, Educational attainment, Marital status, Household size, Religious attendance, Public sector employment, Urban/rural.
INTERACTIONS of VARIABLES TESTED: None.

Design code 39

BASIC MODEL FORM: Linear regression. Includes listwise deletion of missing values.

DETAILED MODEL DESCRIPTION: Multilevel fixed-effects model, defined as a longitudinal model with random-slopes for each higher level unit over time.Hybrid multilevel model including random and fixed-effects. Heirarchical Levels: Estimation Method: Maximum-likelihood (linear).

ADDITIONAL MODEL SPECIFICATIONS: Includes some form of robust clustering.Creates a pseudo panel of sub-groups over time based on income, age and other socio-demographic characteristics.

MEASUREMENT of the SIX DEPENDENT VARIABLES: Linear. Single DV estimated as a scale.

ISSP WAVES INCLUDED: 1996, 2006, 2016. Countries Included: Australia, France, Germany, Japan, New Zealand, Norway, Spain, Sweden, Switzerland, Great Britain, United States.

SPECIAL FEATURES of the COUNTRY SAMPLE:

COUNTRY-LEVEL INDEPENDENT VARIABLES TESTED: Stock of foreign-born, Net migration (over a 1-year period), Social spending as a % of GDP, GDP.

INDIVIDUAL-LEVEL INDEPENDENT VARIABLES TESTED: Age, Sex, Income,

INTERACTIONS of VARIABLES TESTED: None.

Design code 40

BASIC MODEL FORM: Linear regression. Includes listwise deletion of missing values.

DETAILED MODEL DESCRIPTION: Multilevel fixed-effects model, defined as a longitudinal model with random-slopes for each higher level unit over time.Hybrid multilevel model including random and fixed-effects. Heirarchical Levels: Estimation Method: Maximum-likelihood (linear).

ADDITIONAL MODEL SPECIFICATIONS: Includes some form of robust clustering.Creates a pseudo panel of sub-groups over time based on income, age and other socio-demographic characteristics.

MEASUREMENT of the SIX DEPENDENT VARIABLES: Linear. Single DV estimated as a scale.

ISSP WAVES INCLUDED: 1996, 2006, 2016. Countries Included: Australia, France, Germany, Japan, New Zealand, Norway, Spain, Sweden, Switzerland, Great Britain, United States.

SPECIAL FEATURES of the COUNTRY SAMPLE:

COUNTRY-LEVEL INDEPENDENT VARIABLES TESTED: Stock of foreign-born, Net migration (over a 1-year period), Percentage change in foreign-born stock (1-year), Employment rate, Social spending as a % of GDP.

INDIVIDUAL-LEVEL INDEPENDENT VARIABLES TESTED: Age, Sex, Employment status, Income, Educational attainment.

INTERACTIONS of VARIABLES TESTED: None.

Design code 41

BASIC MODEL FORM: Logistic regression. Includes listwise deletion of missing values.

DETAILED MODEL DESCRIPTION: A 'two-way fixed-effects' model similar to that of Brady & Finnigan with the addition of robust clustered standard errors. Includes dummy variables for countries and years. Estimation Method: Maximum-likelihood (binomial/categorical).

ADDITIONAL MODEL SPECIFICATIONS: Includes some form of robust clustering.

MEASUREMENT of the SIX DEPENDENT VARIABLES: Dichotomized. Each DV estimated in separate models.

ISSP WAVES INCLUDED: 1996, 2006, 2016. Countries Included: Australia, Canada, France, Germany, Ireland, Japan, New Zealand, Norway, Spain, Sweden, Switzerland, Great Britain, United States.

SPECIAL FEATURES of the COUNTRY SAMPLE: Includes only the original 13 countries used by Brady & Finnigan.

COUNTRY-LEVEL INDEPENDENT VARIABLES TESTED: Stock of foreign-born, Net migration (over a 1-year period), Employment rate, Social spending as a % of GDP, GDP, Gini, Multiculturalism Policy Index (or equivalent).

INDIVIDUAL-LEVEL INDEPENDENT VARIABLES TESTED: Age, Age-squared, Sex, Employment status, Income, Educational attainment.

INTERACTIONS of VARIABLES TESTED: None.

Design code 42

BASIC MODEL FORM: Linear regression. Includes listwise deletion of missing values.

DETAILED MODEL DESCRIPTION: A 'two-way fixed-effects' similar to that of Brady & Finnigan. Includes dummy variables for countries and years. Estimation Method: Ordinary least squares.

ADDITIONAL MODEL SPECIFICATIONS: None.

MEASUREMENT of the SIX DEPENDENT VARIABLES: Dichotomized. Each DV estimated in separate models.

ISSP WAVES INCLUDED: 1985, 1990, 1996, 2006, 2016. Countries Included: Australia, Canada, France, Germany, Ireland, Japan, New Zealand, Norway, Spain, Sweden, Switzerland, Great Britain, United States.

SPECIAL FEATURES of the COUNTRY SAMPLE: Includes only the original 13 countries used by Brady & Finnigan.

COUNTRY-LEVEL INDEPENDENT VARIABLES TESTED: Stock of foreign-born, Net migration (over a 1-year period), Employment rate, Social spending as a % of GDP, Stock of non-Western immigrants.

INDIVIDUAL-LEVEL INDEPENDENT VARIABLES TESTED: Age, Age-squared, Sex, Employment status, Income, Educational attainment.

INTERACTIONS of VARIABLES TESTED: None.

Design code 43

BASIC MODEL FORM: Logistic regression. Includes listwise deletion of missing values.

DETAILED MODEL DESCRIPTION: A 'two-way fixed-effects' similar to that of Brady & Finnigan. Includes dummy variables for countries and years. Estimation Method: Maximum-likelihood (binomial/categorical).

ADDITIONAL MODEL SPECIFICATIONS: None.

MEASUREMENT of the SIX DEPENDENT VARIABLES: Dichotomized. Each DV estimated in separate models.

ISSP WAVES INCLUDED: 1996, 2006. Countries Included: Australia, Canada, Germany, Ireland, Japan, New Zealand, Norway, Spain, Sweden, Great Britain, United States.

SPECIAL FEATURES of the COUNTRY SAMPLE:

COUNTRY-LEVEL INDEPENDENT VARIABLES TESTED: Stock of foreign-born, Net migration (over a 1-year period), Employment rate, Social spending as a % of GDP, Anti-immigrant sentiment aggregated (from other surveys).

INDIVIDUAL-LEVEL INDEPENDENT VARIABLES TESTED: Age, Age-squared, Sex, Employment status, Income, Educational attainment.

INTERACTIONS of VARIABLES TESTED: None.

Design code 44

BASIC MODEL FORM: Linear regression. Includes listwise deletion of missing values.

DETAILED MODEL DESCRIPTION: Multilevel random-effects model employed, defined as having random intercepts, fixed-slopes and unique error variance at each higher level. Heirarchical Levels: country-year, country, Multilevel structural equation model with random intercepts, fixed-slopes and unique error variance at each higher level. Estimation Method: Maximum-likelihood (linear).

ADDITIONAL MODEL SPECIFICATIONS: None.

MEASUREMENT of the SIX DEPENDENT VARIABLES: Linear. Single DV estimated as a scale.

ISSP WAVES INCLUDED: 1996, 2006, 2016. Countries Included: Czechia, France, Germany, Hungary, Israel, Japan, Latvia, New Zealand, Norway, Slovenia, Spain, Sweden, Switzerland, Great Britain, United States.

SPECIAL FEATURES of the COUNTRY SAMPLE: Includes Eastern Europe.

COUNTRY-LEVEL INDEPENDENT VARIABLES TESTED: Stock of foreign-born, Net migration (over a 1-year period), Employment rate, Social spending as a % of GDP.

INDIVIDUAL-LEVEL INDEPENDENT VARIABLES TESTED: Age, Age-squared, Sex, Employment status, Income,

INTERACTIONS of VARIABLES TESTED: None.

Design code 45

BASIC MODEL FORM: Logistic regression. Includes listwise deletion of missing values.
DETAILED MODEL DESCRIPTION: A 'two-way fixed-effects' model similar to that of Brady & Finnigan with the addition of robust clustered standard errors. Includes dummy variables for countries and years. Estimation Method: Maximum-likelihood (binomial/categorical).
ADDITIONAL MODEL SPECIFICATIONS: Includes some form of robust clustering.Employs survey weighting as provided by the ISSP.
MEASUREMENT of the SIX DEPENDENT VARIABLES: Dichotomized. Each DV estimated in separate models.
ISSP WAVES INCLUDED: 1996, 2006, 2016. Countries Included: Australia, Canada, France, Germany, Ireland, Japan, New Zealand, Norway, Spain, Sweden, Switzerland, Great Britain, United States.
SPECIAL FEATURES of the COUNTRY SAMPLE: Includes only the original 13 countries used by Brady & Finnigan.
COUNTRY-LEVEL INDEPENDENT VARIABLES TESTED: Stock of foreign-born, Net migration (over a 1-year period), Employment rate, Social spending as a % of GDP, GDP, Gini.
INDIVIDUAL-LEVEL INDEPENDENT VARIABLES TESTED: Age, Age-squared, Sex, Employment status, Income, Educational attainment.
INTERACTIONS of VARIABLES TESTED: None.

Design code 46

BASIC MODEL FORM: Logistic regression. Includes listwise deletion of missing values.
DETAILED MODEL DESCRIPTION: A 'two-way fixed-effects' similar to that of Brady & Finnigan. Includes dummy variables for countries and years. Estimation Method: Maximum-likelihood (binomial/categorical).
ADDITIONAL MODEL SPECIFICATIONS: None.
MEASUREMENT of the SIX DEPENDENT VARIABLES: Dichotomized. Each DV estimated in separate models.
ISSP WAVES INCLUDED: 1996, 2006, 2016. Countries Included: Australia, Canada, Denmark, Finland, France, Germany, Ireland, Japan, New Zealand, Norway, Spain, Sweden, Switzerland, Great Britain, United States.
SPECIAL FEATURES of the COUNTRY SAMPLE:
COUNTRY-LEVEL INDEPENDENT VARIABLES TESTED: Stock of foreign-born, Percentage change in foreign-born stock (1-year), Percentage change in foreign-born stock (10-year), Employment rate, Social spending as a % of GDP, Stock of non-Western immigrants.
INDIVIDUAL-LEVEL INDEPENDENT VARIABLES TESTED: Age, Age-squared, Sex, Employment status, Income, Educational attainment.
INTERACTIONS of VARIABLES TESTED: None.

Design code 47

BASIC MODEL FORM: Linear regression. Includes listwise deletion of missing values.

DETAILED MODEL DESCRIPTION: A 'two-way fixed-effects' model similar to that of Brady & Finnigan with the addition of robust clustered standard errors. Includes dummy variables for countries and years. Estimation Method: Maximum-likelihood (linear).Bayesian.

ADDITIONAL MODEL SPECIFICATIONS: Includes some form of robust clustering.Bootstraps the small number of countries to obtain robust estimates (e.g., leave-one-out or jackknife estimator).Employs survey weighting as provided by the ISSP.

MEASUREMENT of the SIX DEPENDENT VARIABLES: Linear. Single DV estimated as a scale.

ISSP WAVES INCLUDED: 1990, 1996, 2006, 2016. Countries Included: Australia, Canada, Chile, Croatia, Denmark, Finland, France, Germany, Hungary, Ireland, Israel, Italy, Japan, Latvia, New Zealand, Norway, Poland, Russia, Slovenia, Spain, Sweden, Switzerland, Great Britain, United States.

SPECIAL FEATURES of the COUNTRY SAMPLE: Includes Eastern Europe.Includes all available countries with relevant measures.

COUNTRY-LEVEL INDEPENDENT VARIABLES TESTED: Stock of foreign-born, Net migration (over a 1-year period), Employment rate, Social spending as a % of GDP.

INDIVIDUAL-LEVEL INDEPENDENT VARIABLES TESTED: Age, Age-squared, Sex, Employment status, Income, Educational attainment.

INTERACTIONS of VARIABLES TESTED: None.

Design code 48

BASIC MODEL FORM: Logistic regression. Includes listwise deletion of missing values.

DETAILED MODEL DESCRIPTION: A 'two-way fixed-effects' model similar to that of Brady & Finnigan with the addition of robust clustered standard errors. Includes dummy variables for countries and years. Estimation Method: Maximum-likelihood (binomial/categorical).Bayesian.

ADDITIONAL MODEL SPECIFICATIONS: Includes some form of robust clustering.Bootstraps the small number of countries to obtain robust estimates (e.g., leave-one-out or jackknife estimator).Employs survey weighting as provided by the ISSP.

MEASUREMENT of the SIX DEPENDENT VARIABLES: Dichotomized. Each DV estimated in separate models.

ISSP WAVES INCLUDED: 1990, 1996, 2006, 2016. Countries Included: Australia, Canada, Chile, Croatia, Denmark, Finland, France, Germany, Hungary, Ireland, Israel, Italy, Japan, Latvia, New Zealand, Norway, Poland, Russia, Slovenia, Spain, Sweden, Switzerland, Great Britain, United States.

SPECIAL FEATURES of the COUNTRY SAMPLE: Includes Eastern Europe.Includes all available countries with relevant measures.

COUNTRY-LEVEL INDEPENDENT VARIABLES TESTED: Stock of foreign-born, Net migration (over a 1-year period), Employment rate, Social spending as a % of GDP.

INDIVIDUAL-LEVEL INDEPENDENT VARIABLES TESTED: Age, Age-squared, Sex, Employment status, Income, Educational attainment.

INTERACTIONS of VARIABLES TESTED: None.

Design code 49

BASIC MODEL FORM: Logistic regression. Includes listwise deletion of missing values.
DETAILED MODEL DESCRIPTION: A 'two-way fixed-effects' similar to that of Brady & Finnigan. Includes dummy variables for countries and years. Estimation Method: Maximum-likelihood (binomial/categorical).
ADDITIONAL MODEL SPECIFICATIONS: Includes some form of robust clustering.
MEASUREMENT of the SIX DEPENDENT VARIABLES: Linear. Single DV estimated as a scale.
ISSP WAVES INCLUDED: 1996, 2006. Countries Included: Australia, Canada, Denmark, Finland, France, Germany, Ireland, Japan, Netherlands, New Zealand, Norway, Portugal, Spain, Sweden, Switzerland, Great Britain, United States.
SPECIAL FEATURES of the COUNTRY SAMPLE: Includes only the original 17 countries analyzed by Brady & Finnigan.
COUNTRY-LEVEL INDEPENDENT VARIABLES TESTED: Stock of foreign-born, Net migration (over a 1-year period), Employment rate, Social spending as a % of GDP.
INDIVIDUAL-LEVEL INDEPENDENT VARIABLES TESTED: Age, Age-squared, Sex, Employment status, Income, Educational attainment.
INTERACTIONS of VARIABLES TESTED: None.

Design code 50

BASIC MODEL FORM: Ordered logistic regression. Includes listwise deletion of missing values.

DETAILED MODEL DESCRIPTION: Multilevel random-effects model employed, defined as having random intercepts, fixed-slopes and unique error variance at each higher level. Heirarchical Levels: country.Categorical. Each DV estimated in separate models. Estimation Method: Maximum-likelihood (binomial/categorical).

ADDITIONAL MODEL SPECIFICATIONS: Includes some form of robust clustering.

MEASUREMENT of the SIX DEPENDENT VARIABLES: Categorical. Each DV estimated in separate models.

ISSP WAVES INCLUDED: 1996, 2006, 2016. Countries Included: Australia, Belgium, Canada, Chile, Croatia, Czechia, Denmark, Finland, France, Germany, Hungary, Iceland, Ireland, Japan, South Korea, Latvia, New Zealand, Norway, Slovakia, Slovenia, Spain, Sweden, Switzerland, Great Britain, United States.

SPECIAL FEATURES of the COUNTRY SAMPLE: Includes Eastern Europe.Includes all available countries with relevant measures.

COUNTRY-LEVEL INDEPENDENT VARIABLES TESTED: Stock of foreign-born, Net migration (over a 1-year period), Percentage change in foreign-born stock (1-year), Percentage change in foreign-born stock (10-year), Employment rate, Social spending as a % of GDP, GDP, Gini.

INDIVIDUAL-LEVEL INDEPENDENT VARIABLES TESTED: Age, Age-squared, Sex, Employment status, Educational attainment, Household size, Respondent interested in politics.

INTERACTIONS of VARIABLES TESTED: None.

Design code 51

BASIC MODEL FORM: Logistic regression. Includes listwise deletion of missing values.

DETAILED MODEL DESCRIPTION: A 'two-way fixed-effects' similar to that of Brady & Finnigan. Includes dummy variables for countries and years. Estimation Method: Maximum-likelihood (binomial/categorical).

ADDITIONAL MODEL SPECIFICATIONS: Bootstraps the small number of countries to obtain robust estimates (e.g., leave-one-out or jackknife estimator).

MEASUREMENT of the SIX DEPENDENT VARIABLES: Dichotomized. Each DV estimated in separate models.

ISSP WAVES INCLUDED: 1996,2006, 2016. Countries Included: Australia, Canada, France, Germany, Ireland, Japan, New Zealand, Norway, Spain, Sweden, Switzerland, Great Britain, United States.

SPECIAL FEATURES of the COUNTRY SAMPLE: Includes only the original 13 countries used by Brady & Finnigan.

COUNTRY-LEVEL INDEPENDENT VARIABLES TESTED: Stock of foreign-born, Net migration (over a 1-year period), Employment rate, Social spending as a % of GDP.

INDIVIDUAL-LEVEL INDEPENDENT VARIABLES TESTED: Age, Age-squared, Sex, Employment status, Income, Educational attainment.

INTERACTIONS of VARIABLES TESTED: None.

Design code 52

BASIC MODEL FORM: Logistic regression. Includes listwise deletion of missing values.

DETAILED MODEL DESCRIPTION: A 'two-way fixed-effects' model similar to that of Brady & Finnigan with the addition of robust clustered standard errors. Includes dummy variables for countries and years.Multilevel random-effects model employed, defined as having random intercepts, fixed-slopes and unique error variance at each higher level. Heirarchical Levels: country-year, country, year.Cross-Classified Model Estimation Method: Ordinary least squares.

ADDITIONAL MODEL SPECIFICATIONS: Bootstraps the small number of countries to obtain robust estimates (e.g., leave-one-out or jackknife estimator).

MEASUREMENT of the SIX DEPENDENT VARIABLES: Dichotomized. Each DV estimated in separate models. Cross-Classified Model

ISSP WAVES INCLUDED: 1990,1996,2006, 2016. Countries Included: Australia, Canada, France, Germany, Ireland, Japan, New Zealand, Norway, Spain, Sweden, Switzerland, Great Britain, United States.

SPECIAL FEATURES of the COUNTRY SAMPLE: Includes only the original 13 countries used by Brady & Finnigan.

COUNTRY-LEVEL INDEPENDENT VARIABLES TESTED: Stock of foreign-born, Net migration (over a 1-year period), Ethnic fractionalization/Herfindahl index.

INDIVIDUAL-LEVEL INDEPENDENT VARIABLES TESTED: Age, Sex, Employment status,

INTERACTIONS of VARIABLES TESTED: None.

Design code 53

BASIC MODEL FORM: Logistic regression. Includes listwise deletion of missing values.

DETAILED MODEL DESCRIPTION: A 'two-way fixed-effects' model similar to that of Brady & Finnigan with the addition of robust clustered standard errors. Includes dummy variables for countries and years. Estimation Method: Ordinary least squares.

ADDITIONAL MODEL SPECIFICATIONS: Includes some form of robust clustering.Bootstraps the small number of countries to obtain robust estimates (e.g., leave-one-out or jackknife estimator).

MEASUREMENT of the SIX DEPENDENT VARIABLES: Dichotomized. Each DV estimated in separate models.

ISSP WAVES INCLUDED: 1996, 2006. Countries Included: Australia, Canada, Denmark, Finland, France, Germany, Ireland, Japan, Netherlands, New Zealand, Norway, Portugal, Spain, Sweden, Switzerland, Great Britain, United States.

SPECIAL FEATURES of the COUNTRY SAMPLE: Includes only the original 17 countries analyzed by Brady & Finnigan.

COUNTRY-LEVEL INDEPENDENT VARIABLES TESTED: Stock of foreign-born, Net migration (over a 1-year period), Ethnic fractionalization/Herfindahl index.

INDIVIDUAL-LEVEL INDEPENDENT VARIABLES TESTED: Age, Sex, Employment status,

INTERACTIONS of VARIABLES TESTED: None.

Design code 54

BASIC MODEL FORM: Linear regression. Includes listwise deletion of missing values.
DETAILED MODEL DESCRIPTION: A 'two-way fixed-effects' similar to that of Brady & Finnigan. Includes dummy variables for countries and years. Estimation Method: Maximum-likelihood (linear).
ADDITIONAL MODEL SPECIFICATIONS: None.
MEASUREMENT of the SIX DEPENDENT VARIABLES: Linear. Single DV estimated as a scale.
ISSP WAVES INCLUDED: 1996, 2006. Countries Included: Australia, Canada, France, Germany, Ireland, Japan, New Zealand, Norway, Spain, Sweden, Switzerland, Great Britain, United States.
SPECIAL FEATURES of the COUNTRY SAMPLE: Includes only the original 13 countries used by Brady & Finnigan.
COUNTRY-LEVEL INDEPENDENT VARIABLES TESTED: Stock of foreign-born, Net migration (over a 1-year period), Employment rate, Social spending as a % of GDP, Socio-cultural proximity scale to immigrants (on average), Ethnic fractionalization/Herfindahl index.
INDIVIDUAL-LEVEL INDEPENDENT VARIABLES TESTED: Age, Age-squared, Sex, Employment status, Income, Educational attainment.
INTERACTIONS of VARIABLES TESTED: None.

Design code 55

BASIC MODEL FORM: Linear regression. Includes listwise deletion of missing values.

DETAILED MODEL DESCRIPTION: Multilevel random-effects model employed, defined as having random intercepts, fixed-slopes and unique error variance at each higher level. Heirarchical Levels: country. Estimation Method: Maximum-likelihood (linear).

ADDITIONAL MODEL SPECIFICATIONS: Includes some form of robust clustering.

MEASUREMENT of the SIX DEPENDENT VARIABLES: Linear. Single DV estimated as a scale.

ISSP WAVES INCLUDED: 2006, 2016. Countries Included: Australia, Belgium, Canada, Croatia, Czechia, Denmark, France, Germany, Hungary, Ireland, Japan, Latvia, New Zealand, Norway, Poland, Slovakia, Slovenia, Spain, Sweden, Switzerland, Great Britain, United States.

SPECIAL FEATURES of the COUNTRY SAMPLE: Includes Eastern Europe.Includes all available countries with relevant measures.

COUNTRY-LEVEL INDEPENDENT VARIABLES TESTED: Stock of foreign-born, Net migration (over a 1-year period), Employment rate, Social spending as a % of GDP, Multiculturalism Policy Index (or equivalent), Welfare state regime types.

INDIVIDUAL-LEVEL INDEPENDENT VARIABLES TESTED: Age, Age-squared, Sex, Employment status, Income, Educational attainment.

INTERACTIONS of VARIABLES TESTED: None.

Design code 56

BASIC MODEL FORM: Logistic regression. Includes listwise deletion of missing values.
DETAILED MODEL DESCRIPTION: A 'two-way fixed-effects' similar to that of Brady & Finnigan. Includes dummy variables for countries and years. Estimation Method: Maximum-likelihood (binomial/categorical).
ADDITIONAL MODEL SPECIFICATIONS: None.
MEASUREMENT of the SIX DEPENDENT VARIABLES: Dichotomized. Each DV estimated in separate models.
ISSP WAVES INCLUDED: 1996,2006, 2016. Countries Included: Australia, Canada, Croatia, Czechia, Denmark, France, Germany, Hungary, Ireland, Israel, Japan, Latvia, New Zealand, Norway, Poland, Slovenia, Spain, Sweden, Switzerland, Great Britain, United States, Taiwan.
SPECIAL FEATURES of the COUNTRY SAMPLE: Includes Eastern Europe.Includes all available countries with relevant measures.
COUNTRY-LEVEL INDEPENDENT VARIABLES TESTED: Stock of foreign-born, Net migration (over a 1-year period), Employment rate, Social spending as a % of GDP, Welfare state regime types.
INDIVIDUAL-LEVEL INDEPENDENT VARIABLES TESTED: Age, Age-squared, Sex, Employment status, Income, Educational attainment.
INTERACTIONS of VARIABLES TESTED: None.

Design code 57

BASIC MODEL FORM: Linear regression. Includes listwise deletion of missing values.

DETAILED MODEL DESCRIPTION: Multilevel random-effects model employed, defined as having random intercepts, fixed-slopes and unique error variance at each higher level. Heirarchical Levels: country-year, country. Estimation Method: Maximum-likelihood (linear).

ADDITIONAL MODEL SPECIFICATIONS: Includes some form of robust clustering.

MEASUREMENT of the SIX DEPENDENT VARIABLES: Linear. Single DV estimated as a scale.

ISSP WAVES INCLUDED: 2006, 2016. Countries Included: Australia, Belgium, Canada, Czechia, Denmark, Finland, France, Germany, Hungary, Iceland, Ireland, Japan, South Korea, Latvia, Netherlands, New Zealand, Norway, Poland, Portugal, Slovakia, Slovenia, Spain, Sweden, Switzerland, Great Britain, United States.

SPECIAL FEATURES of the COUNTRY SAMPLE: Includes Eastern Europe.Includes all available countries with relevant measures.

COUNTRY-LEVEL INDEPENDENT VARIABLES TESTED: Stock of foreign-born, Net migration (over a 1-year period), Employment rate, Change in refugee stock, Social spending as a % of GDP, GDP, Gini, Multiculturalism Policy Index (or equivalent).

INDIVIDUAL-LEVEL INDEPENDENT VARIABLES TESTED: Age, Age-squared, Sex, Employment status, Income, Educational attainment.

INTERACTIONS of VARIABLES TESTED: None.

Design code 58

BASIC MODEL FORM: Logistic regression. Includes listwise deletion of missing values.

DETAILED MODEL DESCRIPTION: A 'two-way fixed-effects' similar to that of Brady & Finnigan. Includes dummy variables for countries and years. Estimation Method: Maximum-likelihood (binomial/categorical).

ADDITIONAL MODEL SPECIFICATIONS: None.

MEASUREMENT of the SIX DEPENDENT VARIABLES: Dichotomized. Each DV estimated in separate models.

ISSP WAVES INCLUDED: 1996, 2006, 2016. Countries Included: Australia, Canada, France, Germany, Ireland, Japan, New Zealand, Norway, Spain, Sweden, Switzerland, Great Britain, United States.

SPECIAL FEATURES of the COUNTRY SAMPLE: Includes only the original 13 countries used by Brady & Finnigan.

COUNTRY-LEVEL INDEPENDENT VARIABLES TESTED: Stock of foreign-born, Net migration (over a 1-year period), Percentage change in foreign-born stock (1-year), Employment rate, Social spending as a % of GDP.

INDIVIDUAL-LEVEL INDEPENDENT VARIABLES TESTED: Age, Age-squared, Sex, Employment status, Income, Educational attainment.

INTERACTIONS of VARIABLES TESTED: None.

Design code 59

BASIC MODEL FORM: Linear regression. Includes listwise deletion of missing values.

DETAILED MODEL DESCRIPTION: Multilevel random-effects model employed, defined as having random intercepts, fixed-slopes and unique error variance at each higher level. Heirarchical Levels: country, Multilevel structural equation model with random intercepts, fixed-slopes and unique error variance at each higher level. Estimation Method: Maximum-likelihood (linear).

ADDITIONAL MODEL SPECIFICATIONS: None.

MEASUREMENT of the SIX DEPENDENT VARIABLES: Linear. Each DV estimated in separate models.

ISSP WAVES INCLUDED: 1996, 2006. Countries Included: Australia, Canada, France, Germany, Ireland, Japan, New Zealand, Norway, Spain, Sweden, Switzerland, Great Britain, United States.

SPECIAL FEATURES of the COUNTRY SAMPLE: Includes only the original 13 countries used by Brady & Finnigan.

COUNTRY-LEVEL INDEPENDENT VARIABLES TESTED: Stock of foreign-born, Net migration (over a 1-year period), Employment rate, Social spending as a % of GDP.

INDIVIDUAL-LEVEL INDEPENDENT VARIABLES TESTED: Age, Age-squared, Sex, Employment status, Income, Educational attainment.

INTERACTIONS of VARIABLES TESTED: None.

Design code 60

BASIC MODEL FORM: Logistic regression. Includes listwise deletion of missing values.

DETAILED MODEL DESCRIPTION: A 'two-way fixed-effects' similar to that of Brady & Finnigan. Includes dummy variables for countries and years. Estimation Method: Maximum-likelihood (binomial/categorical).

ADDITIONAL MODEL SPECIFICATIONS: None.

MEASUREMENT of the SIX DEPENDENT VARIABLES: Dichotomized. Each DV estimated in separate models.

ISSP WAVES INCLUDED: 1996, 2006. Countries Included: Australia, Canada, Denmark, Finland, France, Germany, Ireland, Japan, Netherlands, New Zealand, Norway, Portugal, Spain, Sweden, Switzerland, Great Britain, United States.

SPECIAL FEATURES of the COUNTRY SAMPLE: Includes only the original 17 countries analyzed by Brady & Finnigan.

COUNTRY-LEVEL INDEPENDENT VARIABLES TESTED: Stock of foreign-born, Net migration (over a 1-year period), Employment rate, Social spending as a % of GDP.

INDIVIDUAL-LEVEL INDEPENDENT VARIABLES TESTED: Age, Age-squared, Sex, Employment status, Income, Educational attainment.

INTERACTIONS of VARIABLES TESTED: None.

Design code 61

BASIC MODEL FORM: Ordered logistic regression. Includes listwise deletion of missing values.
DETAILED MODEL DESCRIPTION: A 'two-way fixed-effects' model similar to that of Brady & Finnigan with the addition of robust clustered standard errors. Includes dummy variables for countries and years. Estimation Method: Maximum-likelihood (binomial/categorical).
ADDITIONAL MODEL SPECIFICATIONS: Includes some form of robust clustering.Employs survey weighting as provided by the ISSP.
MEASUREMENT of the SIX DEPENDENT VARIABLES: Categorical. Each DV estimated in separate models.
ISSP WAVES INCLUDED: 1996,2006, 2016. Countries Included: Australia, Canada, France, Germany, Ireland, Japan, New Zealand, Norway, Spain, Sweden, Switzerland, Great Britain, United States.
SPECIAL FEATURES of the COUNTRY SAMPLE: Includes only the original 13 countries used by Brady & Finnigan.
COUNTRY-LEVEL INDEPENDENT VARIABLES TESTED: Stock of foreign-born, Net migration (over a 1-year period), Percentage change in foreign-born stock (10-year), Employment rate, Social spending as a % of GDP, GDP, Gini.
INDIVIDUAL-LEVEL INDEPENDENT VARIABLES TESTED: Age, Age-squared, Sex, Employment status, Income, Educational attainment.
INTERACTIONS of VARIABLES TESTED: None.

Design code 63

BASIC MODEL FORM: Logistic regression. Includes listwise deletion of missing values.

DETAILED MODEL DESCRIPTION: Multilevel random-effects model employed, defined as having random intercepts, fixed-slopes and unique error variance at each higher level. Heirarchical Levels: country-year, country. Estimation Method: Maximum-likelihood (binomial/categorical).

ADDITIONAL MODEL SPECIFICATIONS: Includes some form of robust clustering.Bootstraps the small number of countries to obtain robust estimates (e.g., leave-one-out or jackknife estimator).

MEASUREMENT of the SIX DEPENDENT VARIABLES: Dichotomized. Each DV estimated in separate models.

ISSP WAVES INCLUDED: 1996, 2006, 2016. Countries Included: Australia, Canada, Denmark, Finland, France, Germany, Ireland, New Zealand, Norway, Portugal, Spain, Sweden, Switzerland, Great Britain, United States.

SPECIAL FEATURES of the COUNTRY SAMPLE:

COUNTRY-LEVEL INDEPENDENT VARIABLES TESTED: Stock of foreign-born, Employment rate, Social spending as a % of GDP.

INDIVIDUAL-LEVEL INDEPENDENT VARIABLES TESTED: Age, Age-squared, Sex, Employment status, Income, Educational attainment.

INTERACTIONS of VARIABLES TESTED: None.

Design code 64

BASIC MODEL FORM: Linear regression.

DETAILED MODEL DESCRIPTION: Multilevel random-effects model employed, defined as having random intercepts, fixed-slopes and unique error variance at each higher level. Heirarchical Levels: country, Multilevel structural equation model with random intercepts, fixed-slopes and unique error variance at each higher level. Estimation Method: Maximum-likelihood (linear).

ADDITIONAL MODEL SPECIFICATIONS: Includes some form of robust clustering.

MEASUREMENT of the SIX DEPENDENT VARIABLES: Linear. Single DV estimated as a scale.

ISSP WAVES INCLUDED: 2016. Countries Included: Australia, Belgium, Chile, Czechia, Denmark, Finland, France, Germany, Hungary, Iceland, Israel, Japan, South Korea, Latvia, Lithuania, Norway, Slovakia, Slovenia, Spain, Sweden, Switzerland, Great Britain, United States, Turkey.

SPECIAL FEATURES of the COUNTRY SAMPLE: Includes Eastern Europe.Includes all available countries with relevant measures.

COUNTRY-LEVEL INDEPENDENT VARIABLES TESTED: Stock of foreign-born, Net migration (over a 1-year period), Percentage change in foreign-born stock (10-year), Employment rate, Social spending as a % of GDP, Welfare state regime types.

INDIVIDUAL-LEVEL INDEPENDENT VARIABLES TESTED: Age, Age-squared, Sex, Employment status, Income, Educational attainment, Marital status, Household size, Urban/rural.

INTERACTIONS of VARIABLES TESTED: None.

Design code 65

BASIC MODEL FORM: Logistic regression. Includes listwise deletion of missing values.

DETAILED MODEL DESCRIPTION: Multilevel random-effects model employed, defined as having random intercepts, fixed-slopes and unique error variance at each higher level. Heirarchical Levels: year. Estimation Method: Maximum-likelihood (binomial/categorical).

ADDITIONAL MODEL SPECIFICATIONS: None.

MEASUREMENT of the SIX DEPENDENT VARIABLES: Dichotomized. Each DV estimated in separate models. Analyzes regions of Germany instead of countries.

ISSP WAVES INCLUDED: only 2016. Countries Included: only Germany analyzed by Federal States within Germany.

SPECIAL FEATURES of the COUNTRY SAMPLE:

COUNTRY-LEVEL INDEPENDENT VARIABLES TESTED: Stock of foreign-born, Percentage change in foreign-born stock (1-year), Unemployment rate, Social spending as a % of GDP, Stock of non-Western immigrants.

INDIVIDUAL-LEVEL INDEPENDENT VARIABLES TESTED: Age, Age-squared, Sex, Employment status, Educational attainment.

INTERACTIONS of VARIABLES TESTED: None.

Design code 66

BASIC MODEL FORM: Logistic regression. Includes listwise deletion of missing values.

DETAILED MODEL DESCRIPTION: A 'two-way fixed-effects' model similar to that of Brady & Finnigan with the addition of robust clustered standard errors. Includes dummy variables for countries and years. Estimation Method: Maximum-likelihood (binomial/categorical).

ADDITIONAL MODEL SPECIFICATIONS: Includes some form of robust clustering.

MEASUREMENT of the SIX DEPENDENT VARIABLES: Dichotomized. Each DV estimated in separate models.

ISSP WAVES INCLUDED: 1996, 2006, 2016. Countries Included: Australia, France, Germany, Japan, New Zealand, Norway, Spain, Sweden, Switzerland, Great Britain, United States.

SPECIAL FEATURES of the COUNTRY SAMPLE:

COUNTRY-LEVEL INDEPENDENT VARIABLES TESTED: Stock of foreign-born, Net migration (over a 1-year period), Employment rate, Social spending as a % of GDP.

INDIVIDUAL-LEVEL INDEPENDENT VARIABLES TESTED: Age, Age-squared, Sex, Employment status, Income, Educational attainment.

INTERACTIONS of VARIABLES TESTED: None.

Design code 67

BASIC MODEL FORM: Ordered logistic regression. Engages in multiple imputation.

DETAILED MODEL DESCRIPTION: A 'two-way fixed-effects' similar to that of Brady & Finnigan. Includes dummy variables for countries and years. Estimation Method: Maximum-likelihood (binomial/categorical).

ADDITIONAL MODEL SPECIFICATIONS: None.

MEASUREMENT of the SIX DEPENDENT VARIABLES: Categorical. Each DV estimated in separate models.

ISSP WAVES INCLUDED: 1996, 2006. Countries Included: Australia, Canada, France, Germany, Ireland, Japan, New Zealand, Norway, Spain, Sweden, Switzerland, Great Britain, United States.

SPECIAL FEATURES of the COUNTRY SAMPLE: Includes only the original 13 countries used by Brady & Finnigan.

COUNTRY-LEVEL INDEPENDENT VARIABLES TESTED: Stock of foreign-born, Net migration (over a 1-year period), Employment rate, Social spending as a % of GDP.

INDIVIDUAL-LEVEL INDEPENDENT VARIABLES TESTED: Age, Sex, Employment status, Income, Educational attainment, Subjective left-right self-placement.

INTERACTIONS of VARIABLES TESTED: None.

Design code 68

BASIC MODEL FORM: Logistic regression. Includes listwise deletion of missing values.
DETAILED MODEL DESCRIPTION: A 'two-way fixed-effects' similar to that of Brady & Finnigan. Includes dummy variables for countries and years. Estimation Method: Maximum-likelihood (binomial/categorical).
ADDITIONAL MODEL SPECIFICATIONS: Includes some form of robust clustering.
MEASUREMENT of the SIX DEPENDENT VARIABLES: Dichotomized. Each DV estimated in separate models.
ISSP WAVES INCLUDED: 1996, 2006, 2016. Countries Included: Australia, Canada, Denmark, Finland, France, Western Germany, Ireland, Japan, Netherlands, New Zealand, Norway, Portugal, Spain, Sweden, Switzerland, Great Britain, United States.
SPECIAL FEATURES of the COUNTRY SAMPLE:
COUNTRY-LEVEL INDEPENDENT VARIABLES TESTED: Stock of foreign-born, Net migration (over a 1-year period), Employment rate, Social spending as a % of GDP, Socio-cultural proximity scale to immigrants (on average), Stock of non-Western immigrants.
INDIVIDUAL-LEVEL INDEPENDENT VARIABLES TESTED: Age, Age-squared, Sex, Employment status, Educational attainment, Marital status, Household size, Urban/rural.
INTERACTIONS of VARIABLES TESTED: None.

Design code 69

BASIC MODEL FORM: Ordered logistic regression.
DETAILED MODEL DESCRIPTION: Multilevel random-effects model employed, defined as having random intercepts, fixed-slopes and unique error variance at each higher level. Heirarchical Levels: country-year. Estimation Method: Bayesian.
ADDITIONAL MODEL SPECIFICATIONS: None.
MEASUREMENT of the SIX DEPENDENT VARIABLES: Categorical. Each DV estimated in separate models.
ISSP WAVES INCLUDED: 1996, 2006. Countries Included: Australia, Canada, Chile, Croatia, Czechia, Denmark, Finland, France, Western Germany, Eastern Germany, Hungary, Ireland, Israel, Italy, Japan, South Korea, Latvia, Netherlands, New Zealand, Norway, Philippines, Poland, Portugal, Russia, Slovakia, Slovenia, Spain, Sweden, Switzerland, Great Britain, Northern Ireland, United States, South Africa, Taiwan, Uruguay, Venezuela.
SPECIAL FEATURES of the COUNTRY SAMPLE: Includes Eastern Europe.Includes all available countries with relevant measures.
COUNTRY-LEVEL INDEPENDENT VARIABLES TESTED: Stock of foreign-born, Net migration (over a 1-year period), GDP.
INDIVIDUAL-LEVEL INDEPENDENT VARIABLES TESTED: Age, Age-squared, Sex, Employment status, Income, Educational attainment, Marital status, Household size, Religious attendance, Urban/rural.
INTERACTIONS of VARIABLES TESTED: None.

Design code 70

BASIC MODEL FORM: Linear regression.
DETAILED MODEL DESCRIPTION: A 'two-way fixed-effects' model similar to that of Brady & Finnigan with the addition of robust clustered standard errors. Includes dummy variables for countries and years.Structural equation model. Estimation Method: Maximum-likelihood (linear).
ADDITIONAL MODEL SPECIFICATIONS: Includes some form of robust clustering.
MEASUREMENT of the SIX DEPENDENT VARIABLES: Linear. Single DV estimated as a scale. Structural equation model.
ISSP WAVES INCLUDED: 1996, 2006, 2016. Countries Included: Australia, Canada, Czechia, France, Germany, Hungary, Japan, Latvia, New Zealand, Norway, Slovenia, Spain, Sweden, Switzerland, Great Britain, United States.
SPECIAL FEATURES of the COUNTRY SAMPLE: Includes Eastern Europe.
COUNTRY-LEVEL INDEPENDENT VARIABLES TESTED: Stock of foreign-born, Percentage change in foreign-born stock (1-year), Employment rate, GDP, Multiculturalism Policy Index (or equivalent).
INDIVIDUAL-LEVEL INDEPENDENT VARIABLES TESTED: Age, Age-squared, Sex, Employment status, Educational attainment.
INTERACTIONS of VARIABLES TESTED: Net migration*Unemployment, Foreign-born stock*Unemployment.

Design code 71

BASIC MODEL FORM: Ordered logistic regression. Includes listwise deletion of missing values.
DETAILED MODEL DESCRIPTION: A 'two-way fixed-effects' model similar to that of Brady & Finnigan with the addition of robust clustered standard errors. Includes dummy variables for countries and years. Estimation Method: Maximum-likelihood (binomial/categorical).
ADDITIONAL MODEL SPECIFICATIONS: Includes some form of robust clustering.
MEASUREMENT of the SIX DEPENDENT VARIABLES: Categorical. Each DV estimated in separate models.
ISSP WAVES INCLUDED: 1996, 2006, 2016. Countries Included: Australia, Canada, Czechia, France, Germany, Ireland, Japan, Latvia, New Zealand, Norway, Spain, Sweden, Switzerland, Great Britain, United States.
SPECIAL FEATURES of the COUNTRY SAMPLE:
COUNTRY-LEVEL INDEPENDENT VARIABLES TESTED: Stock of foreign-born, Net migration (over a 1-year period), Unemployment rate, Social spending as a % of GDP.
INDIVIDUAL-LEVEL INDEPENDENT VARIABLES TESTED: Age, Age-squared, Sex, Employment status, Income, Educational attainment.
INTERACTIONS of VARIABLES TESTED: Net migration*Individual educational attainment, Net migration*Unemployment, Foreign-born stock*Individual educational attainment, Foreign-born stock*Unemployment.

Design code 72

BASIC MODEL FORM: Linear regression. Includes listwise deletion of missing values.
DETAILED MODEL DESCRIPTION: A 'two-way fixed-effects' model similar to that of Brady & Finnigan with the addition of robust clustered standard errors. Includes dummy variables for countries and years. Estimation Method: Ordinary least squares.
ADDITIONAL MODEL SPECIFICATIONS: Includes some form of robust clustering.
MEASUREMENT of the SIX DEPENDENT VARIABLES: Linear. Each DV estimated in separate models.
ISSP WAVES INCLUDED: 1996, 2006, 2016. Countries Included: Australia, Canada, Chile, Czechia, Denmark, Finland, France, Germany, Hungary, Ireland, Israel, Japan, South Korea, Latvia, New Zealand, Norway, Poland, Slovenia, Spain, Sweden, Switzerland, Great Britain, United States.
SPECIAL FEATURES of the COUNTRY SAMPLE: Includes Eastern Europe.Includes all available countries with relevant measures.
COUNTRY-LEVEL INDEPENDENT VARIABLES TESTED: Stock of foreign-born, Net migration (over a 1-year period), Employment rate, Social spending as a % of GDP.
INDIVIDUAL-LEVEL INDEPENDENT VARIABLES TESTED: Age, Age-squared, Sex, Employment status, Income, Educational attainment.
INTERACTIONS of VARIABLES TESTED: Immigration measures squared.

Design code 73

BASIC MODEL FORM: Logistic regression. Includes listwise deletion of missing values.
DETAILED MODEL DESCRIPTION: A 'two-way fixed-effects' similar to that of Brady & Finnigan. Includes dummy variables for countries and years. Estimation Method: Maximum-likelihood (binomial/categorical).
ADDITIONAL MODEL SPECIFICATIONS: None.
MEASUREMENT of the SIX DEPENDENT VARIABLES: Dichotomized. Each DV estimated in separate models.
ISSP WAVES INCLUDED: 1996, 2006, 2016. Countries Included: Australia, Canada, Chile, Czechia, Denmark, Finland, France, Germany, Hungary, Ireland, Israel, Japan, South Korea, Latvia, New Zealand, Norway, Philippines, Poland, Slovenia, Spain, Sweden, Switzerland, Great Britain, United States.
SPECIAL FEATURES of the COUNTRY SAMPLE: Includes Eastern Europe.Includes all available countries with relevant measures.
COUNTRY-LEVEL INDEPENDENT VARIABLES TESTED: Stock of foreign-born, Net migration (over a 1-year period), Employment rate, Social spending as a % of GDP.
INDIVIDUAL-LEVEL INDEPENDENT VARIABLES TESTED: Age, Age-squared, Sex, Employment status, Income, Educational attainment.
INTERACTIONS of VARIABLES TESTED: Net migration*Individual educational attainment, Net migration*Individual age, Net migration*Sex, Foreign-born stock*Individual educational attainment, Foreign-born stock*Individual age, Foreign-born stock*Sex.

Design code 74

BASIC MODEL FORM: Ordered logistic regression. Includes listwise deletion of missing values.

DETAILED MODEL DESCRIPTION: A 'two-way fixed-effects' similar to that of Brady & Finnigan. Includes dummy variables for countries and years. Estimation Method: Maximum-likelihood (binomial/categorical).

ADDITIONAL MODEL SPECIFICATIONS: None.

MEASUREMENT of the SIX DEPENDENT VARIABLES: Categorical. Each DV estimated in separate models.

ISSP WAVES INCLUDED: 1996, 2006, 2016. Countries Included: Australia, Czechia, France, Germany, Hungary, Israel, Japan, Latvia, New Zealand, Norway, Slovenia, Spain, Sweden, Switzerland, Great Britain, United States.

SPECIAL FEATURES of the COUNTRY SAMPLE: Includes Eastern Europe.

COUNTRY-LEVEL INDEPENDENT VARIABLES TESTED: Stock of foreign-born, Net migration (over a 1-year period), Social spending as a % of GDP.

INDIVIDUAL-LEVEL INDEPENDENT VARIABLES TESTED: Age, Age-squared, Sex, Employment status, Income, Educational attainment, Political trust, Political efficacy.

INTERACTIONS of VARIABLES TESTED: None.

Design code 75

BASIC MODEL FORM: Linear regression. Includes listwise deletion of missing values.
DETAILED MODEL DESCRIPTION: Multilevel random-effects model employed, defined as having random intercepts, fixed-slopes and unique error variance at each higher level. Heirarchical Levels: country-year, country, year.Cross-Classified Model Estimation Method: Maximum-likelihood (linear).
ADDITIONAL MODEL SPECIFICATIONS: Includes some form of robust clustering.
MEASUREMENT of the SIX DEPENDENT VARIABLES: Dichotomized. Each DV estimated in separate models. Cross-Classified Model
ISSP WAVES INCLUDED: 1996, 2006, 2016. Countries Included: Australia, Canada, Chile, Croatia, Czechia, Denmark, Finland, France, Germany, Hungary, Ireland, Israel, Japan, Latvia, New Zealand, Norway, Philippines, Poland, Portugal, Slovenia, Spain, Sweden, Switzerland, Great Britain, United States, South Africa.
SPECIAL FEATURES of the COUNTRY SAMPLE: Includes Eastern Europe.Includes all available countries with relevant measures.
COUNTRY-LEVEL INDEPENDENT VARIABLES TESTED: Stock of foreign-born, Net migration (over a 1-year period), Employment rate, Social spending as a % of GDP.
INDIVIDUAL-LEVEL INDEPENDENT VARIABLES TESTED: Age, Age-squared, Sex, Employment status, Income, Educational attainment.
INTERACTIONS of VARIABLES TESTED: None.

Design code 76

BASIC MODEL FORM: Logistic regression. Includes listwise deletion of missing values.
DETAILED MODEL DESCRIPTION: Multilevel random-effects model employed, defined as having random intercepts, fixed-slopes and unique error variance at each higher level. Heirarchical Levels: country. Estimation Method: Maximum-likelihood (binomial/categorical).
ADDITIONAL MODEL SPECIFICATIONS: None.
MEASUREMENT of the SIX DEPENDENT VARIABLES: Dichotomized. Each DV estimated in separate models.
ISSP WAVES INCLUDED: 1996, 2006. Countries Included: Australia, Canada, France, Germany, Ireland, Japan, New Zealand, Norway, Spain, Sweden, Switzerland, Great Britain, United States.
SPECIAL FEATURES of the COUNTRY SAMPLE: Includes only the original 13 countries used by Brady & Finnigan.
COUNTRY-LEVEL INDEPENDENT VARIABLES TESTED: Stock of foreign-born, Net migration (over a 1-year period), Unemployment rate, Social spending as a % of GDP, GDP, Multiculturalism Policy Index (or equivalent), Ethnic fractionalization/Herfindahl index.
INDIVIDUAL-LEVEL INDEPENDENT VARIABLES TESTED: Age, Age-squared, Sex, Employment status, Educational attainment.
INTERACTIONS of VARIABLES TESTED: None.

Design code 77

BASIC MODEL FORM: Linear regression. Engages in multiple imputation.

DETAILED MODEL DESCRIPTION: Multilevel random-effects model employed, defined as having random intercepts, fixed-slopes and unique error variance at each higher level. Heirarchical Levels: country, year. Estimation Method: Bayesian.

ADDITIONAL MODEL SPECIFICATIONS: None.

MEASUREMENT of the SIX DEPENDENT VARIABLES: Linear. Single DV estimated as a scale. Each DV is dirst dichotomized and then used to consturct a latent scale.

ISSP WAVES INCLUDED: 1996, 2006, 2016. Countries Included: Australia, Canada, France, Germany, Ireland, Japan, New Zealand, Norway, Spain, Sweden, Switzerland, Great Britain, United States.

SPECIAL FEATURES of the COUNTRY SAMPLE: Includes only the original 13 countries used by Brady & Finnigan.

COUNTRY-LEVEL INDEPENDENT VARIABLES TESTED: Stock of foreign-born, Net migration (over a 1-year period),

INDIVIDUAL-LEVEL INDEPENDENT VARIABLES TESTED: Age, Age-squared, Sex, Employment status, Educational attainment.

INTERACTIONS of VARIABLES TESTED: Net migration*Individual educational attainment, Foreign-born stock*Individual educational attainment.

Design code 79

BASIC MODEL FORM: Ordered logistic regression. Includes listwise deletion of missing values.
DETAILED MODEL DESCRIPTION: A 'two-way fixed-effects' similar to that of Brady & Finnigan. Includes dummy variables for countries and years. Estimation Method: Maximum-likelihood (binomial/categorical).
ADDITIONAL MODEL SPECIFICATIONS: None.
MEASUREMENT of the SIX DEPENDENT VARIABLES: Categorical. Each DV estimated in separate cumulative-link models.
ISSP WAVES INCLUDED: 1996, 2006, 2016. Countries Included: Australia, Canada, France, Germany, Ireland, Japan, New Zealand, Norway, Spain, Sweden, Switzerland, Great Britain, United States.
SPECIAL FEATURES of the COUNTRY SAMPLE: Includes only the original 13 countries used by Brady & Finnigan.
COUNTRY-LEVEL INDEPENDENT VARIABLES TESTED: Stock of foreign-born, Percentage change in foreign-born stock (1-year), GDP, Gini.
INDIVIDUAL-LEVEL INDEPENDENT VARIABLES TESTED: None.
INTERACTIONS of VARIABLES TESTED: Foreign-born stock*Net migration.

Ranking Derived from Deliberation and Voting

Based on critical differences in research designs we provided the following main theses to start the deliberation:

CASE SELECTION

Brady and Finnigan (2014) argue that rich democracies are appropriate for testing their hypothesis. They identify seventeen in particular (AUS, CAN, DEN, FIN, FRA, DEU, IRE, JPN, NET, NZL, NOR, PRT, ESP, SWE, CHE, UK and US). They analyzed a sub-sample of thirteen due to data availability. Research designs testing our hypothesis should only include some or all of these seventeen countries. Any additional countries are inappropriate for testing the hypothesis.

CLUSTERED STANDARD ERRORS

Brady & Finnigan did not use clustered standard errors in the two-way FE models at the country-level. Therefore, coefficient significance tests use thousands of cases when there are only 13 countries. Therefore, to truly test the CRI hypothesis every study must cluster the standardd errors for all country-level independent variable coefficients. Otherwise the estimates are untrustworthy.

POWER

One team did a power analysis of a 2x13 case bivariate regression, to thest the greatest possible power Brady & Finnigan had in their two-way FE models. If the true effect of immigration on social policy preferences is <0.16 standardized units (i.e., Cohen's d=0.16 assuming standardized scales), they concluded <80% power (at .05 alpha). If similar power analyses were conducted for each research design, those with <80% power must be excluded from the CRI results.

TWO-WAY FE

One participant suggested that the interpretation of two-way FE is not ideal to test the hypothesis as results are not straightforward, and thus difficult to interpret. This is a Thesis against the use of two-way FE. [link to working-paper at the time provided] 'Abstract': "The two-way fixed effects (FE) model, an increasingly popular method for modeling time-series cross-section (TSCS) data, is substantively difficult to interpret because the model's estimates are a complex amalgamation of variation in the over-time and cross-sectional effects. We demonstrate this complexity in the two-way FE estimate through mathematical exposition. As an illustration, we develop a novel simulation that enables us to generate TSCS data with varying over-time and cross-sectional effects and examine the behavior of the two-way FE model as these effects change. We demonstrate that the two-way FE model makes specific assumptions about TSCS datasets, and if these assumptions are not met, the model may be unidentified even if substantial variation exists along both dimensions. Because of the difficulty in interpretation, we do not recommend that applied researchers rely on the two-way FE model except for situations in which the assumptions are well-understood, such as the canonical difference-in-difference design." (paper later published as Kropko and Kubinec 2020).

Vote response options about the "veracity" of the arguments:

0 - False
1 - Improbable
2 - Plausible
3 - Probable
4 - True

In this data frame the majority arguments against or for the thesis are only listed if thye got at least 10 votes and had an average above 3 (so the 4th bar is at least partially filled)

The participants were split into two groups, both groups participated in post design deliberation and voting.

Deliberation Group (participated in a deliberation before designing their research)

https://www.kialo.com/crowdsourced-replication-initiative---research-design-critical-arguments-24397

Control Group (did not participate in pre-design deliberation)

https://www.kialo.com/crowdsourced-replication-initiative---research-design---critical-arguments-24289

The major results are that participants were in favor of clustering standard errors, and opposed to the usage of only rich democracies. We add two columns to give a bonus for studies that have these attributes. Based on the nature of the discussion we must give a positive score to **all** studies using clustered standard errors, not just Two-Way FE, therefore all multilevel models also get this positive scoring.

References

1. H. Wickham, *ggplot2: Elegant Graphics for Data Analysis* (Springer-Verlag New York, 2016).

2. M. Allen, *et al.*, Raincloud plots: a multi-platform tool for robust data visualization. *Wellcome Open Res.* **4**, 63 (2021).

3. Y. Rosseel, lavaan: An R Package for Structural Equation Modeling. *J. Stat. Softw.* **48**, 1–36 (2012).

4. N. Breznau, E. M. Rinke, A. Wuttke, Pre-Analysis Plan: Measuring Routine Researcher Variability in Macro-Comparative Secondary Data Analyses (2018).

5. N. Breznau, Eike Mark Rinke, Alexander Wuttke, The Crowdsourced Replication Initiative Participant Survey (2021) https:/doi.org/10.7910/DVN/UUP8CX.

6. K. Barton, *MuMIn: multi-model inference* (2009). *http://r-forge. r-project. org/projects/mumin/*
